# Supplementary material for: Directed evolution of CRISPR-Cas9 to increase its specificity
Source: Nat Commun. 2018 Aug 6;9:3048. doi: 10.1038/s41467-018-05477-x (PMC6078992; doi:10.1038/s41467-018-05477-x)
Supplement: Supplementary file 1 — Supplementary Information [file 41467_2018_5477_MOESM1_ESM.pdf]

Supplementary Figures for Lee et al.

Directed evolution of CRISPR-Cas9 to increase its specificity

Supplementary Figure 1.

The successful integration of *EMX1* into the genomic DNA of the BW25141 strain was confirmed by colony PCR amplification of the Tn7 attachment site in the glmS gene. A 678 bp product was observed in the absence of an *EMX1* insertion whereas a 1404 bp product was observed in the presence of an *EMX1* insertion. Eight different colonies were picked; amplification of DNA from #1 and #6 resulted in single bands of 1404 bp. #1 was selected for plasmid removal by incubation at 42°C overnight.

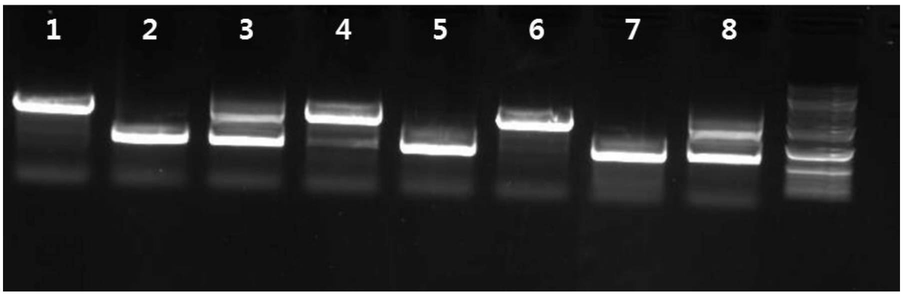

Supplementary Figure 2.

Control reactions for four different Sniper-screens were performed with plasmids containing WT-Cas9 or no insert (null). E. coli death was measured by comparing colony forming units (CFU) on plates with inducers (chloramphenicol + kanamycin + arabinose + ATC; CKA + ATC) and without inducers (chloramphenicol + kanamycin; CK). In both cases, under inducing conditions 100~10,000-fold more E. coli death was observed, caused by the formation of DSBs in genomic DNA (WT-Cas9) or expression of the *ccdB* gene (null).

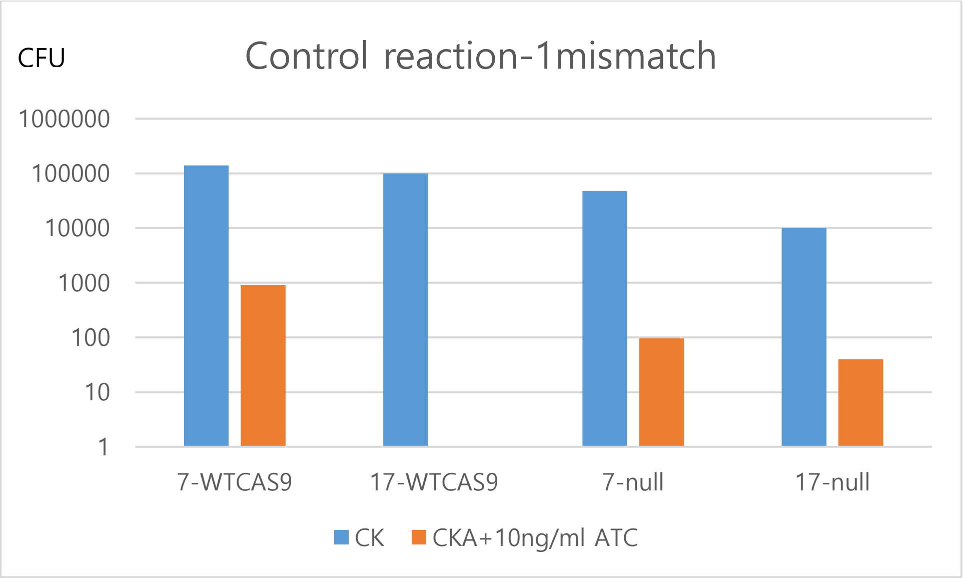

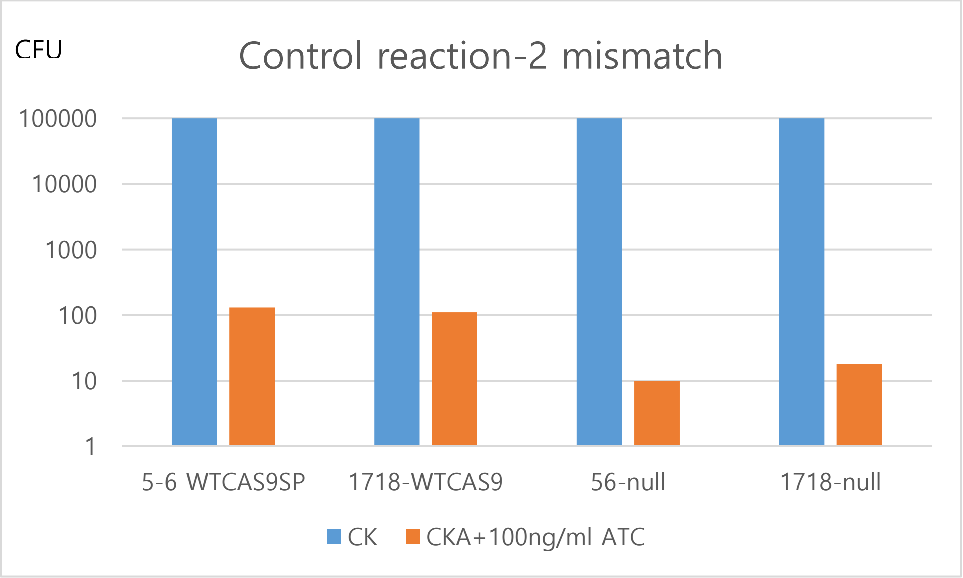

Supplementary Figure 3.

EMX1 sgRNAs mismatched relative to the WT *EMX1* sequence (top row). 20mer *EMX1* target sequence 56: 5th and 6th positions were substituted as designated in upper case characters; 1718: 17th and 18th positions were substituted as designated in upper case characters; 7: 7th position was substituted as designated in upper case characters; 17: 17th position was substituted as designated in upper case characters. The associated indel frequencies are shown in the last column.

	Guide sequence of sgRNA	Target sequence	Indel %
EMX1	gGAGTCCGAGCAGAAGAAGAA	tGAGTCCGAGCAGAAGAAGAA GGG	35
56	gGAGTCCGAGCAGAAagAGAA	tGAGTCCGAGCAGAAagAGAA GGG	5
1718	gGAacCCGAGCAGAAGAAGAA	tGAacCCGAGCAGAAGAAGAA GGG	28
7	gGAGTCCGAGCAGAgGAAGAA	tGAGTCCGAGCAGAgGAAGAA GGG	18
17	gGAGcCCGAGCAGAAGAAGAA	tGAGcCCGAGCAGAAGAAGAA GGG	33

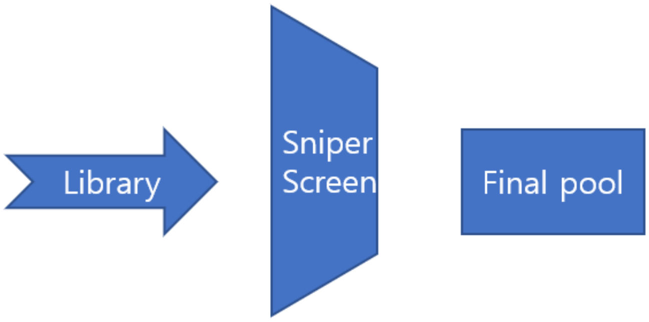

Screening A: Three types of libraries (XL-1 Red, derived from XL-1 Red competent cells from Agilent; Aligent, derived using the Genemorph II error prone PCR kit from Agilent; Clontech, derived using the Diversify PCR random mutagenesis kit from Clontech) were subjected to the Sniper-screen in BW251414-EMX1(56) cells with sgRNA (2 mismatches, at positions 5 and 6) and *ccdB* plasmids. 1G1S(1718): Screening of the results of the library screen in BW25141-EMX1 (1718). 1G2S(1718): Screening of the results from the 1G1S(1718) screen in BW25141-EMX1 (1718). 1G3S(1718): Screening of the results from the 1G2S(1718) screen in BW25141-EMX1 (1718). 2G1S(7): Screening of the shuffled library generated from the results of the 1G3S(17) screen in BW25141-EMX1 (7). 2G2S(7): Screening of the results from the 2G1S(7) screen in BW25141-EMX1 (7). 2G3S(17): Screening of the results from the 2G2S(7) screen in BW25141-EMX1 (17). 2G4S(7): Screening of the results from the 2G3S(7) screen in BW25141-EMX1 (7). 2G5S(17): Screening of the results from the 2G4S(7) screen in BW25141-EMX1 (17). DNA shuffling reaction was performed with 1G4S.

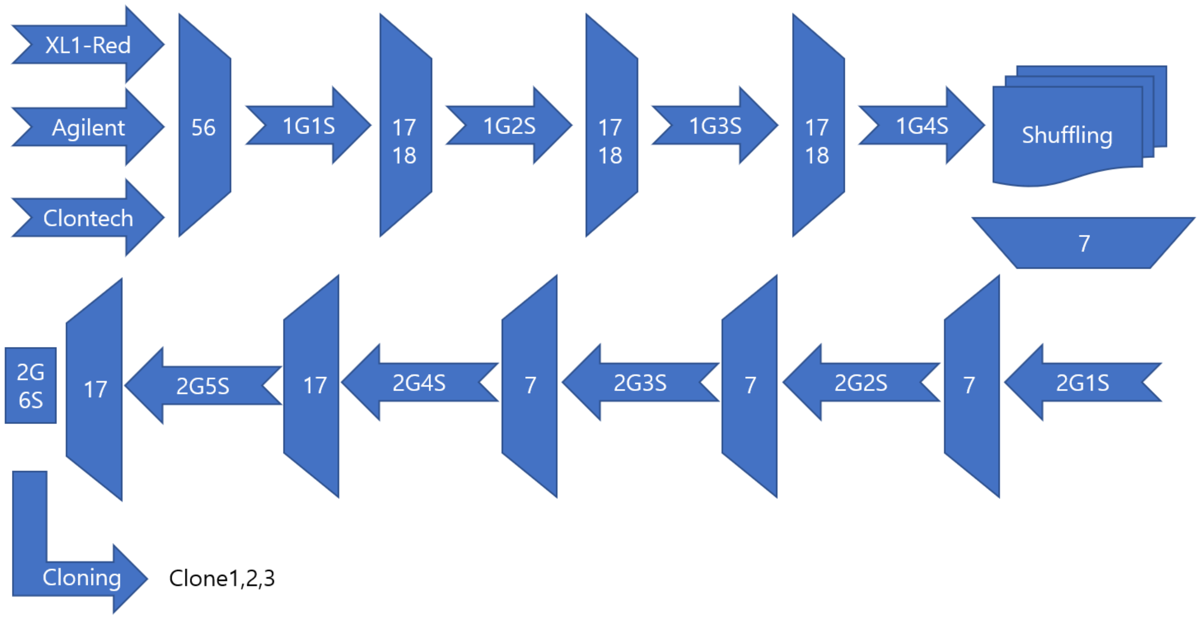

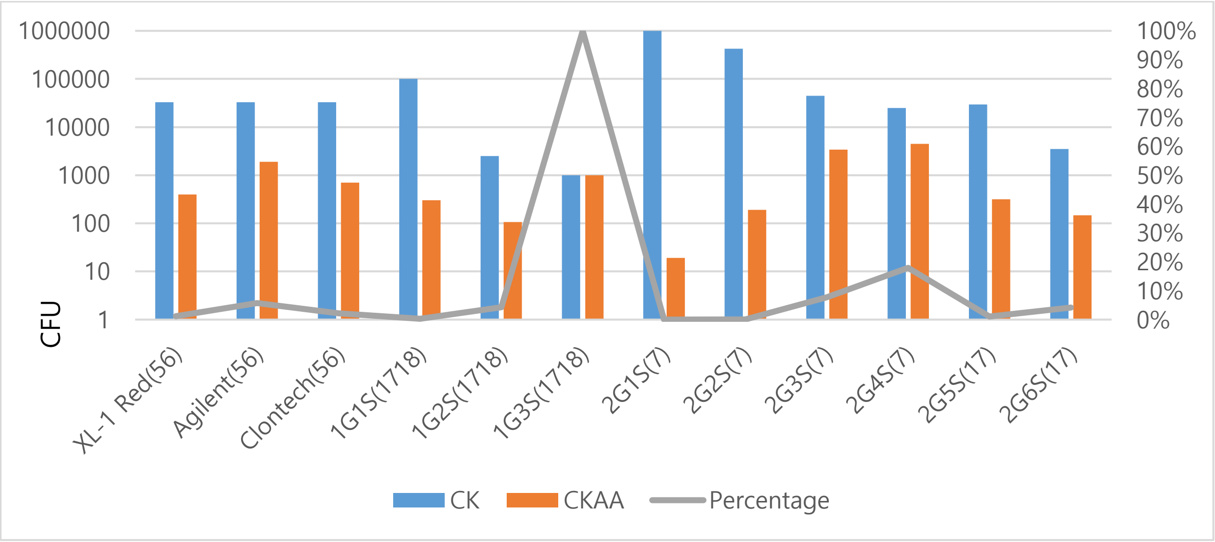

Screening B: Three types of libraries (XL-1 Red, derived from XL-1 Red competent cells from Agilent; Aligent, derived using the Genemorph II error prone PCR kit from Agilent; Clontech, derived using the Diversify PCR random mutagenesis kit from Clontech) were subjected to the Sniper-screen with sgRNA (1 mismatch at position 7) and *ccdB* plasmids. 1G1S(7) (1Generation1Series): Screening of the results from the first library screen in BW25141-EMX1 (7). 1G2S(7): Screening of the results from the 1G1S screen in BW25141-EMX1 (7). 1G3S(7): Screening of the results from the 1G2S(7) screen in BW25141-EMX1 (7). 1G4S(17): Screening of the results from the 1G3S(7) screen in BW25141-EMX1 (17). 1G5S(17): Screening of the results from the 1G4S(17) screen in BW25141-EMX1 (17).

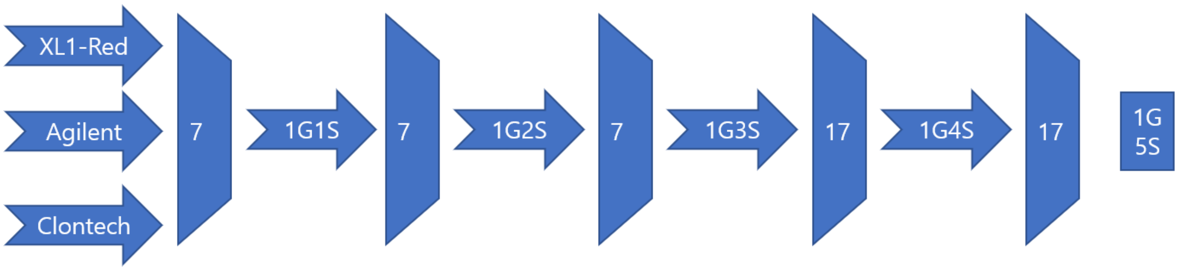

XL1-Red
Agilent
Clontech
7
1G1S
7
1G2S
7
1G3S
17
1G4S
17
1G
5S

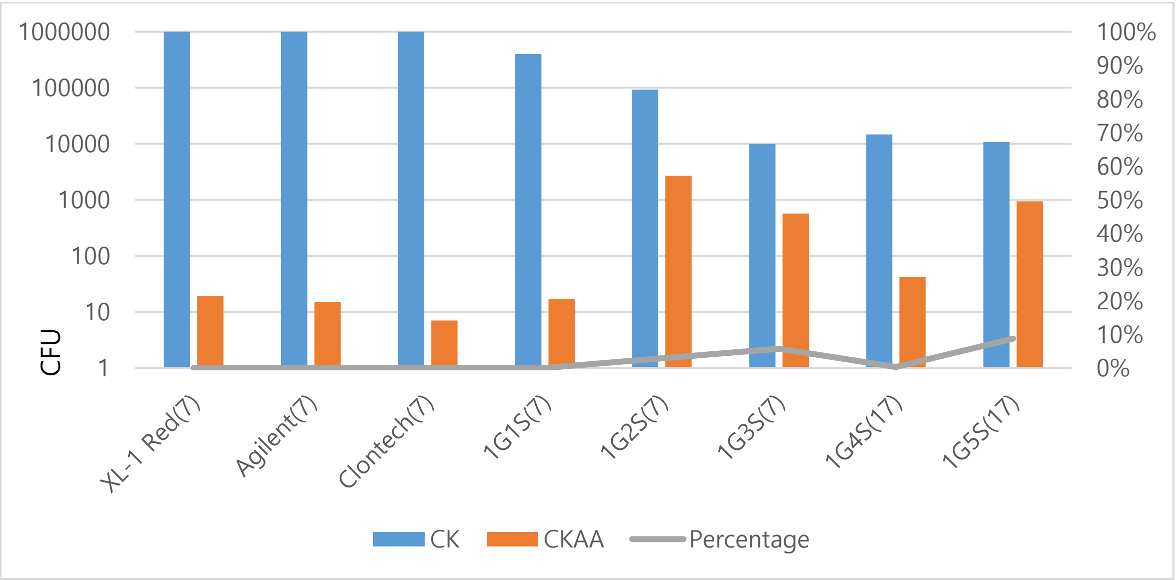

1000000
100000
10000
1000
100
10
1
CFU
100%
90%
80%
70%
60%
50%
40%
30%
20%
10%
0%
XL-1 Red(7)
Agilent(7)
Clontech(7)
1G1S(7)
1G2S(7)
1G3S(7)
1G4S(17)
1G5S(17)
CK
CKAA
Percentage

Supplementary Figure 4.

1) *DMD*

On-target and off-target activities of WT-Cas9 and the pooled library from two different screenings at a target in the human *DMD* gene in HEK293T cells.

DMD **CTTTCTACCTACTGAGTCTG** GGG
CTTTCTACCTACCGAGTCTG GGG

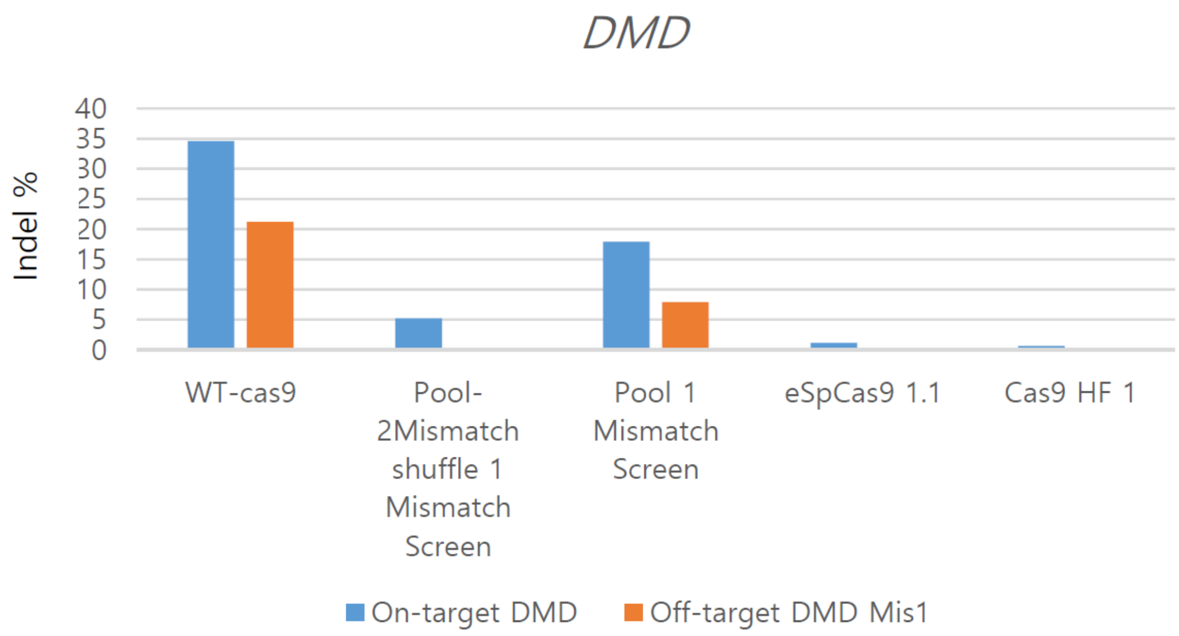

On-target and off-target activities of three different Cas9 variants (Clone-1, 2 and 3) at a target in the human *DMD* gene in HEK293T cells.

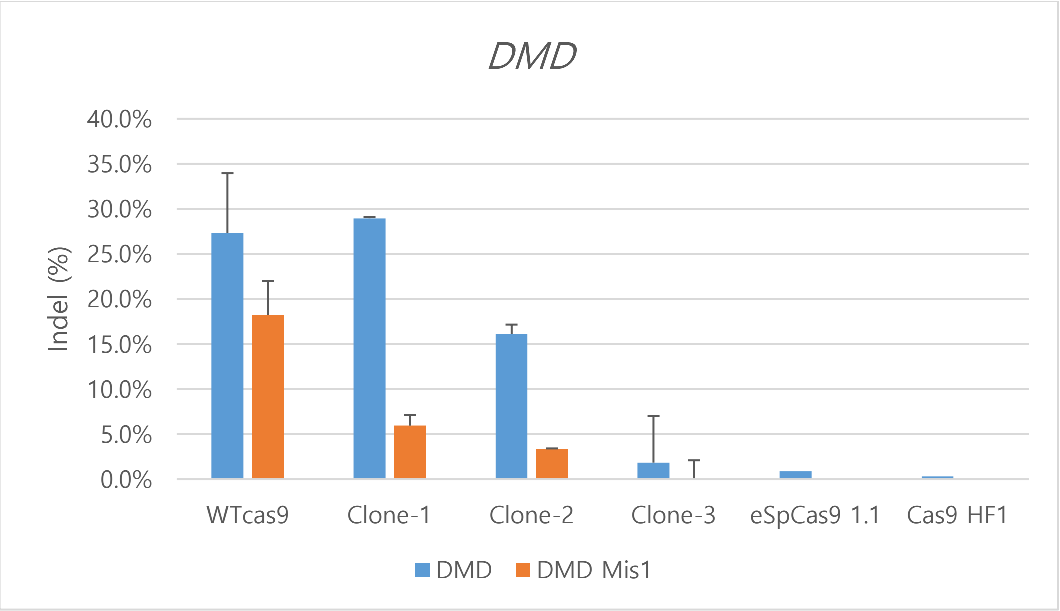

2) *EMX1*

On-target and off-target activities of WT-Cas9 and the pooled library from two different screenings at a target in the human *EMX1* gene in HEK293T cells.

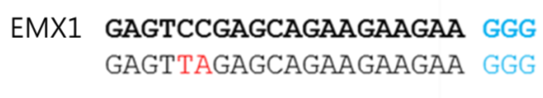

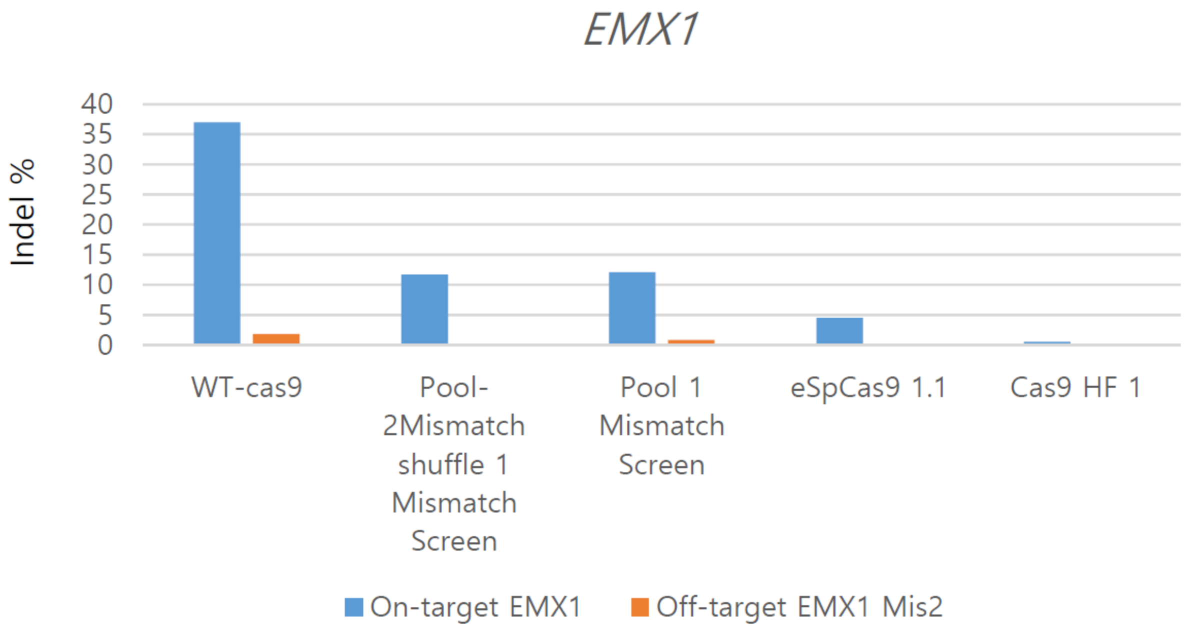

On-target and off-target activities of three different Cas9 variants (Clone-1, 2 and 3) at a target in the human *EMX1* gene in HEK293T cells.

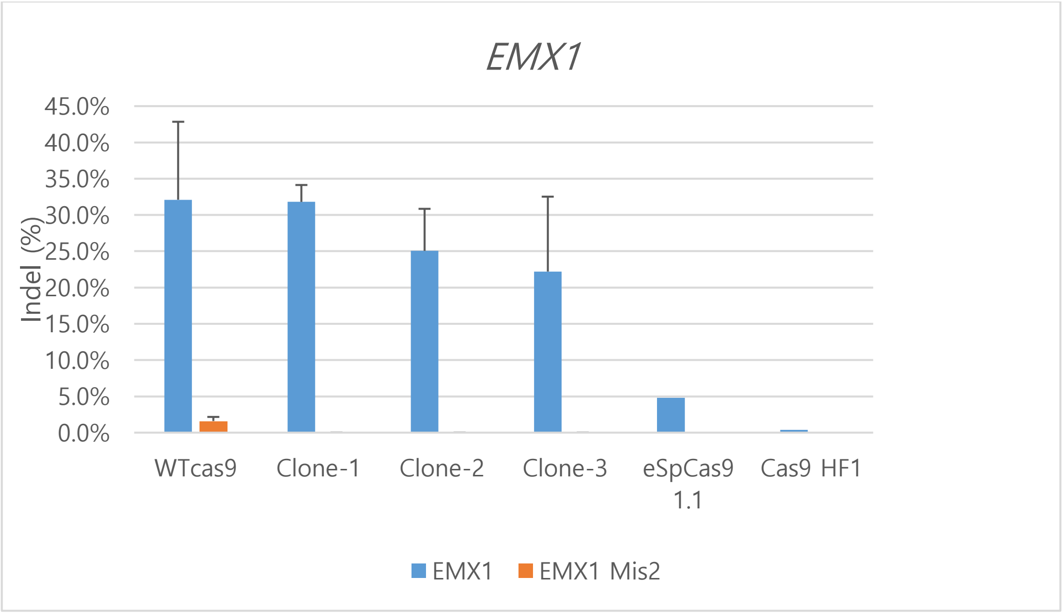

Supplementary Figure 5.

The amino acid mutations in Clone-1, Clone-2 and Clone-3 are listed below.

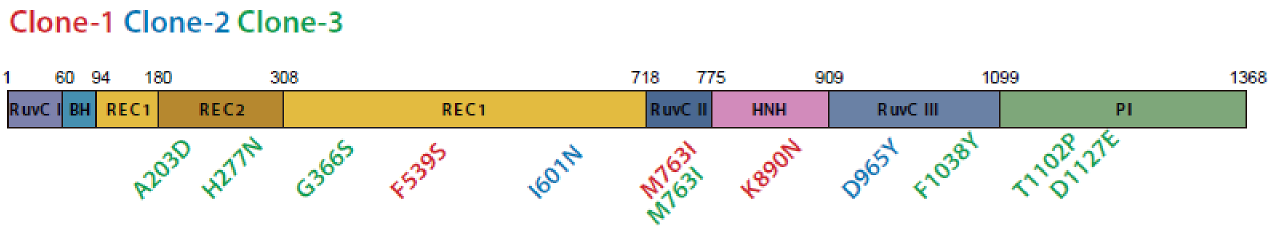

Supplementary Figure 6.

Various mutant forms of Cas9 were made by introducing site directed mutations into WT-Cas9. The indel frequencies were measured in HEK293T cells at the *DMD* and *EMX1* targets.

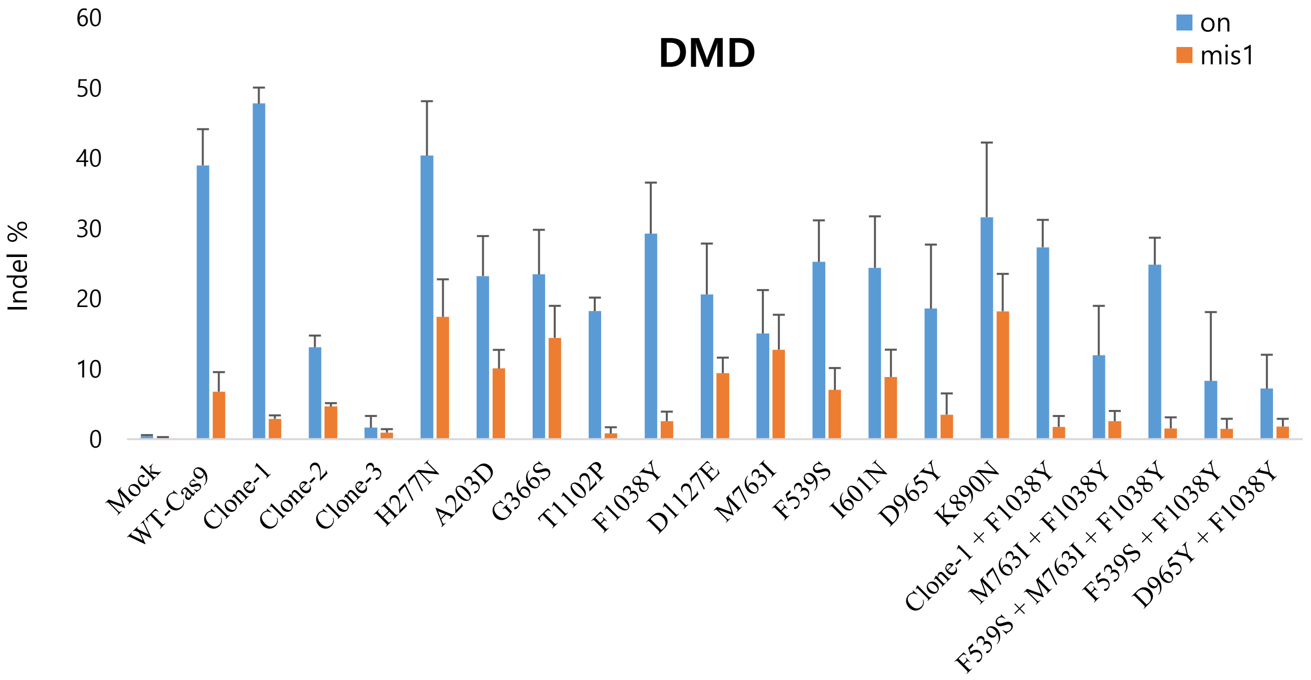

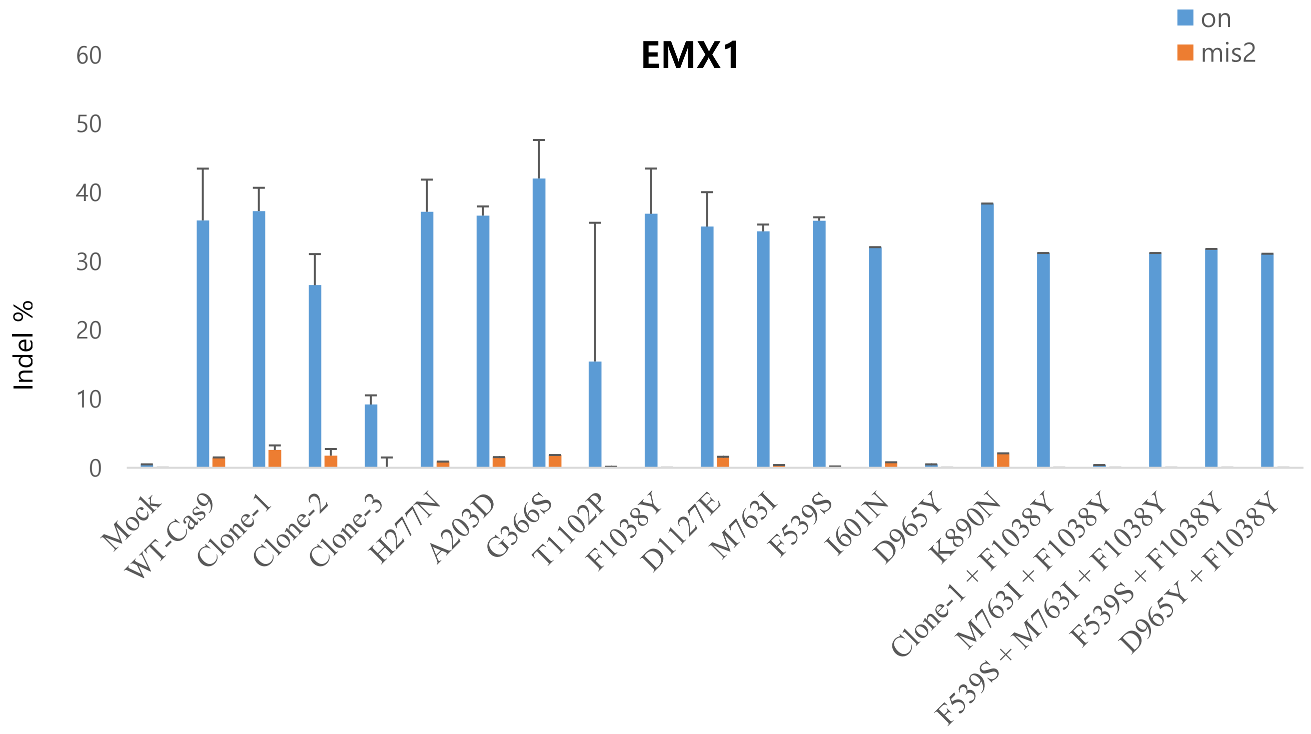

Supplementary Figure 7.

sgRNAs were transcribed by the U6 promoter and thus must start with a guanine at the 5' end, which may (GX19) or may not (gX19 or gX20) complement the on-target sequences. We tested six sgRNAs with mismatched 5' guanines (gX19) and six with matching 5' guanines (GX19).

DMD gX19	DMD gX20	EMX1 GX19	EMX1 gX20
gTTTCTACCTACTGAGTCTG	gCTTTCTACCTACTGAGTCTG	GAGTCCGAGCAGAAGAAGAA	gGAGTCCGAGCAGAAGAAGAA
GAAAGATGGATGACTCAGAC	GGAAAGATGGATGACTCAGAC	CTCAGGCTCGTCTTCTTCTT	ACTCAGGCTCGTCTTCTTCTT

Target	Seqeunce
DMD On	tccCTTTCTACCTACTGAGTCTGGGG
DMD OT	tcaCTTTCTACCTACcGAGTCTGGGG
AAVS On	gctCTCCCTCCCAGGATCCTCTCTGG
AAVS OT	tctCTCCCTCCCAGGATCCTCcCAGG
HBB02 On	cacCTTGCCCCACAGGGCAGTAACGG
HBB02 OT	tgctcaGCCCCACAGGGCAGTAAGGG
HBB03 On	atcCACGTTCACCTTGCCCCACAGGG
HBB03 OT	atcCACGTTCACtTTGCCCCACAGGG
HBB04 On	catCCACGTTCACCTTGCCCCACAGG
HBB04 OT	cttCCACaTTCACCTTGCCCCACAGG
HPRT On	tgcTCGAGATGTGATGAAGGAGATGG
HPRT OT	tttcaGAGATGTGATGtAGGAGAAGG

Target	Seqeunce
FANCF01 On	gatGGAATCCCTTCTGCAGCACCTGG
FANCF01 OT	ccgGGAAcCCCgTCTGCAGCACCAGG
FANCF02 On	ggtGCTGCAGAAGGGATTCCATGAGG
FANCF02 OT	tatGCTGCAGAAGGGATTCCAaGGGG
EMX1 On	cctGAGTCCGAGCAGAAGAAGAAGGG
EMX1 OT	tcaGAGTtaGAGCAGAAGAAGAAGGG
RUNX1 On	ggtGCATTTTCAGGAGGAAGCGATGG
RUNX1 OT	gaaGCATTTTCAGaAGGAAGCaAGGG
ZSCAN2 On	gtaGTGCGGCAAGAGCTTCAGCCGGG
ZSCAN2 OT	ggaGTGtGGCAAGgGCTTCAGCCAGG
HEK4 On	ggtGGCACTGCGGCTGGAGGTGGGGG
HEK4 OT	gtttGCACTGCGGCcGGAGGaGGTGG

Supplementary Figure 8.

On-target and off-target activities of Cas9 variants compared to WT-Cas9 using sgRNAs variable lengths targeting rest of 10 target sites out of 12 targets tested. Results for *FANCF01* and *AAVS* were shown in Figure 2a as examples. Specificity ratios were determined by dividing indel frequencies at on-target sites by those at respective off-target sites. sgRNAs with a matched guanine at the 5' terminus (GX18 or GX19) and those with a mismatched guanine (gX17, gX18, gX19 or gX20) are indicated. N/A (Not Available): Specificity ratios were not calculated when on-target activities were less than 70% of the WT plus 20mer guide sequence. Error bars indicate s.e.m. (*n*=3)

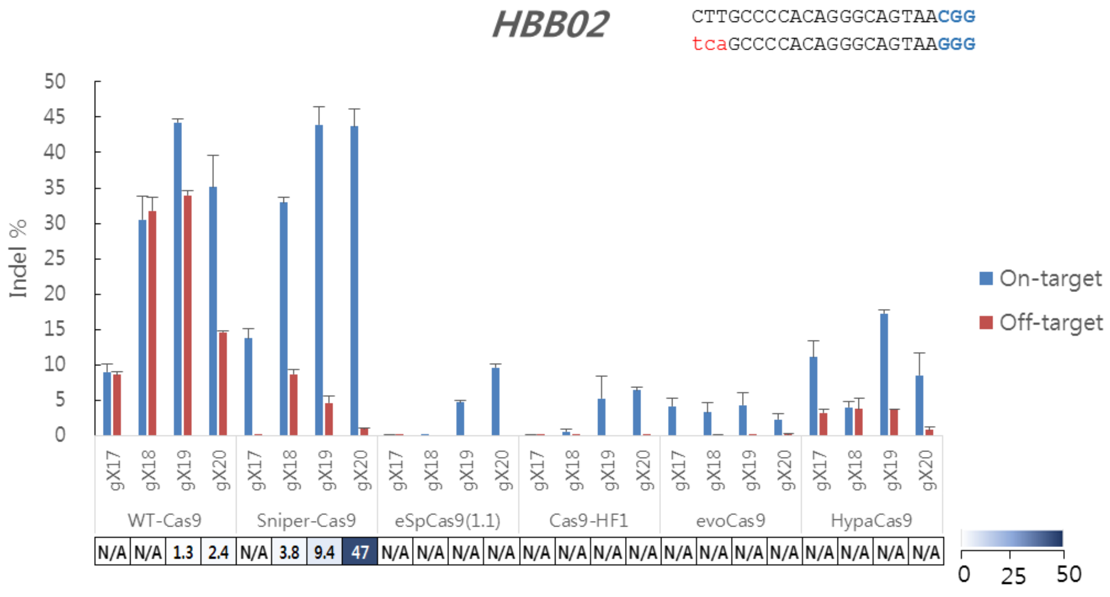

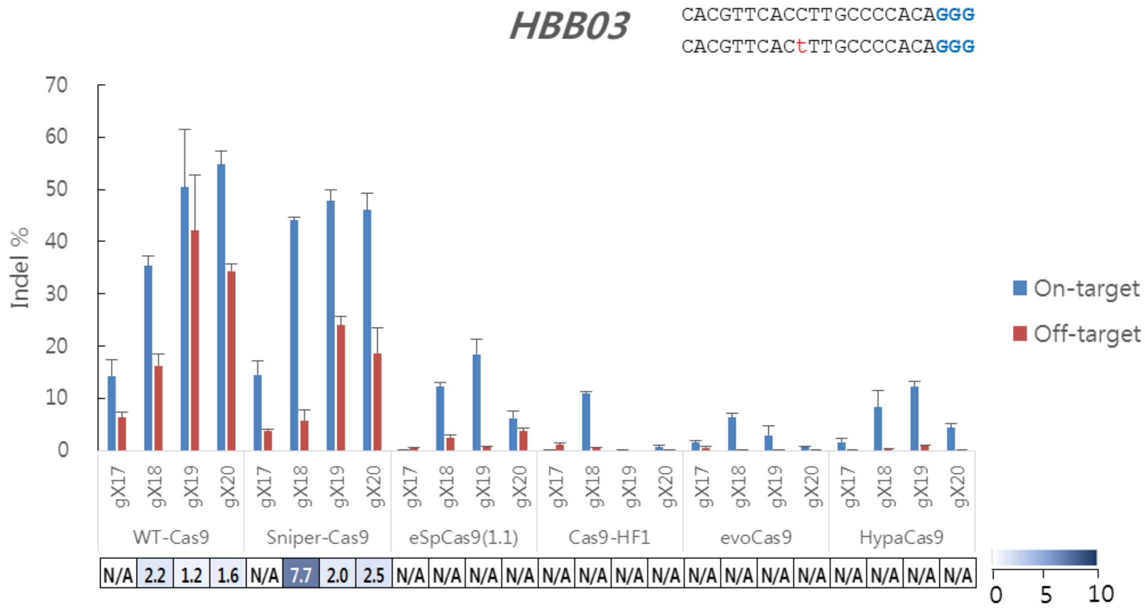

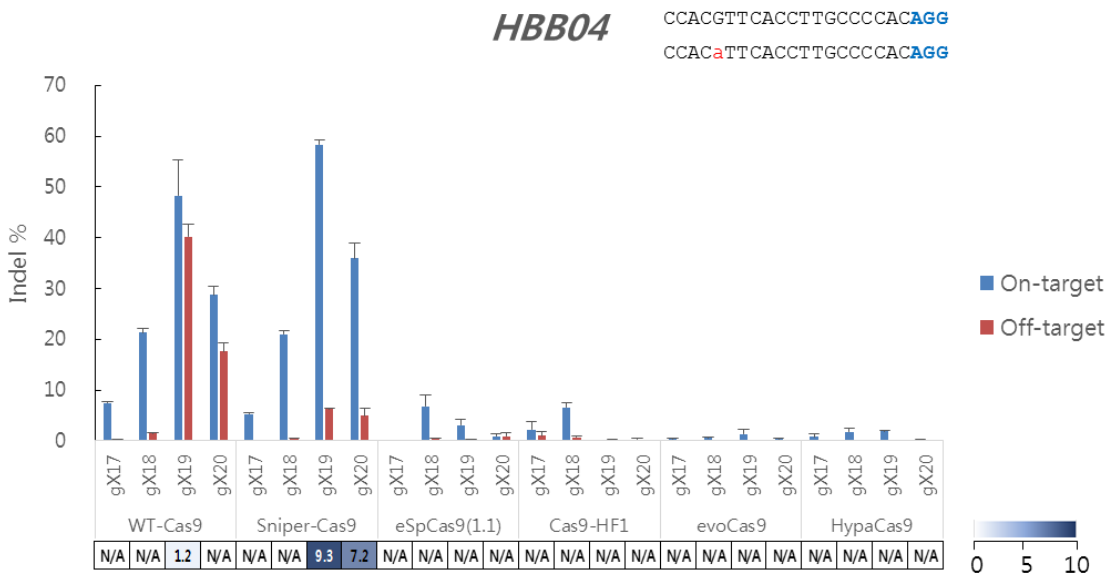

HBB04
CCACGTTCACCTTGCCCCACAGG
CCACaTTCACCTTGCCCCACAGG
Indel %
On-target
Off-target
WT-Cas9
Sniper-Cas9
eSpCas9(1.1)
Cas9-HF1
evoCas9
HypaCas9
N/A N/A 1.2 N/A N/A N/A 9.3 7.2 N/A N/A N/A N/A N/A N/A N/A N/A N/A N/A N/A N/A N/A N/A N/A N/A
0 5 10

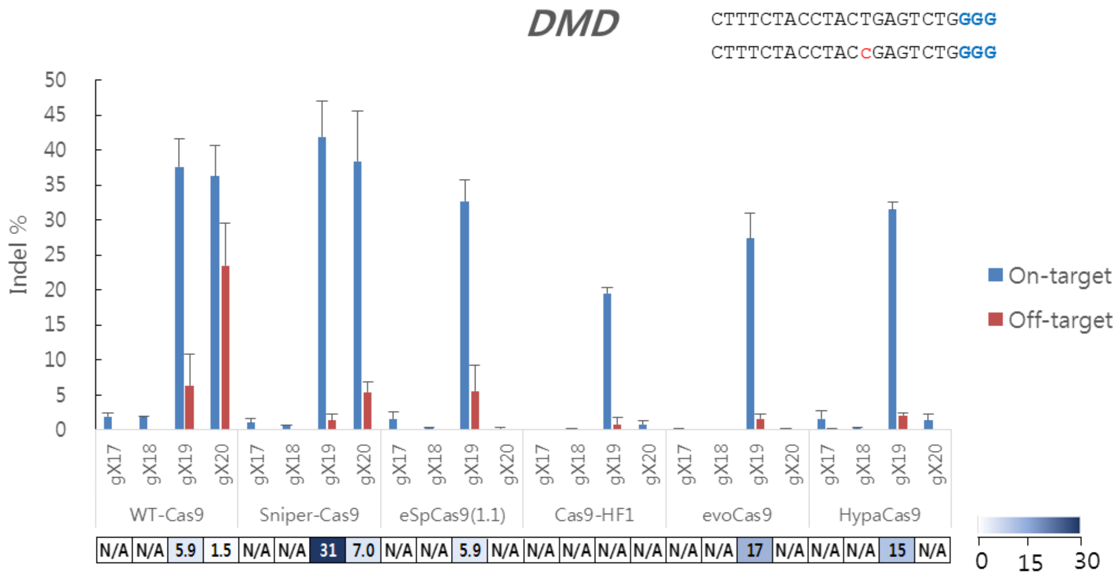

DMD
CTTTCTACCTACTGAGTCTGGGG
CTTTCTACCTACcGAGTCTGGGG
Indel %
On-target
Off-target
WT-Cas9
Sniper-Cas9
eSpCas9(1.1)
Cas9-HF1
evoCas9
HypaCas9
N/A N/A 5.9 1.5 N/A N/A 31 7.0 N/A N/A 5.9 N/A N/A N/A N/A N/A N/A N/A 17 N/A N/A N/A 15 N/A
0 15 30

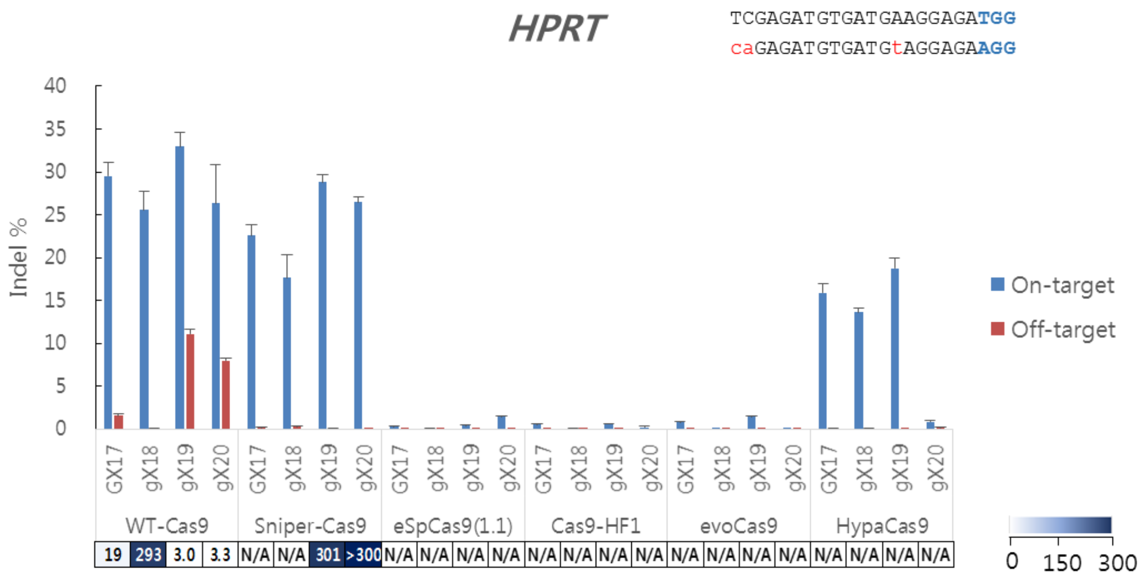
HPRT
TCGAGATGTGATGAAGGAGATGG
caGAGATGTGATGtAGGAGAAGG
Indel %
0
5
10
15
20
25
30
35
40
On-target
Off-target
GX17 gX18 gX19 gX20 GX17 gX18 gX19 gX20 GX17 gX18 gX19 gX20 GX17 gX18 gX19 gX20 GX17 gX18 gX19 gX20 GX17 gX18 gX19 gX20
WT-Cas9
Sniper-Cas9
eSpCas9(1.1)
Cas9-HF1
evoCas9
HypaCas9
19 293 3.0 3.3 N/A N/A 301 >300 N/A N/A N/A N/A N/A N/A N/A N/A N/A N/A N/A N/A N/A N/A N/A N/A
0 150 300

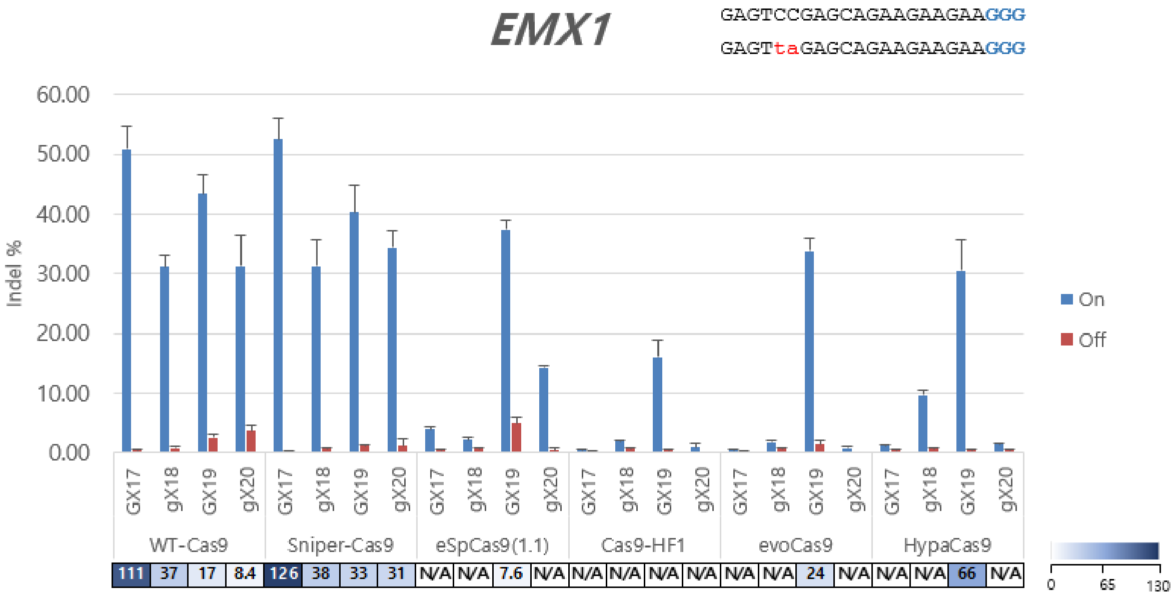
EMX1
GAGTCCGAGCAGAAGAAGAAGGG
GAGTtaGAGCAGAAGAAGAAGGG
Indel %
0.00
10.00
20.00
30.00
40.00
50.00
60.00
On
Off
GX17 gX18 GX19 gX20 GX17 gX18 GX19 gX20 GX17 gX18 GX19 gX20 GX17 gX18 GX19 gX20 GX17 gX18 GX19 gX20 GX17 gX18 GX19 gX20
WT-Cas9
Sniper-Cas9
eSpCas9(1.1)
Cas9-HF1
evoCas9
HypaCas9
111 37 17 8.4 126 38 33 31 N/A N/A 7.6 N/A N/A N/A N/A N/A N/A N/A 24 N/A N/A N/A 66 N/A
0 65 130

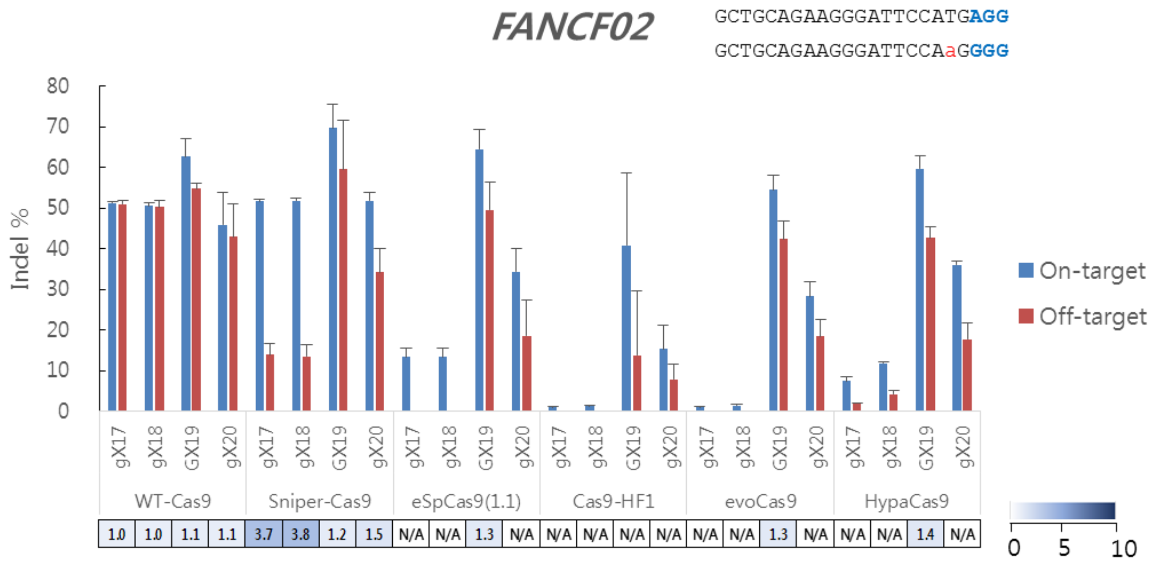
FANCF02
GCTGCAGAAGGGATTCCATGAGG
GCTGCAGAAGGGATTCCAaGGGG
Indel %
0 10 20 30 40 50 60 70 80
On-target
Off-target
gX17 gX18 GX19 gX20 gX17 gX18 GX19 gX20 gX17 gX18 GX19 gX20 gX17 gX18 GX19 gX20 gX17 gX18 GX19 gX20 gX17 gX18 GX19 gX20
WT-Cas9
Sniper-Cas9
eSpCas9(1.1)
Cas9-HF1
evoCas9
HypaCas9
1.0 1.0 1.1 1.1 3.7 3.8 1.2 1.5 N/A N/A 1.3 N/A N/A N/A N/A N/A N/A N/A 1.3 N/A N/A N/A 1.4 N/A
0 5 10

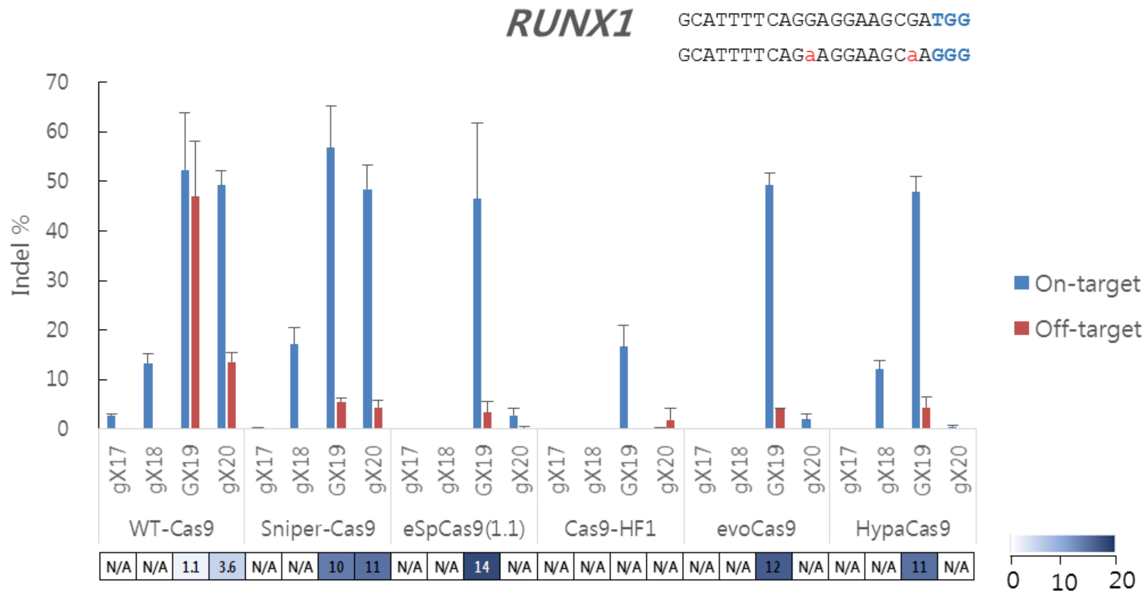
RUNX1
GCATTTTCAGGAGGAAGCGATGG
GCATTTTCAGaAGGAAGCaAGGG
Indel %
0 10 20 30 40 50 60 70
On-target
Off-target
gX17 gX18 GX19 gX20 gX17 gX18 GX19 gX20 gX17 gX18 GX19 gX20 gX17 gX18 GX19 gX20 gX17 gX18 GX19 gX20 gX17 gX18 GX19 gX20
WT-Cas9
Sniper-Cas9
eSpCas9(1.1)
Cas9-HF1
evoCas9
HypaCas9
N/A N/A 1.1 3.6 N/A N/A 10 11 N/A N/A 14 N/A N/A N/A N/A N/A N/A N/A 12 N/A N/A N/A 11 N/A
0 10 20

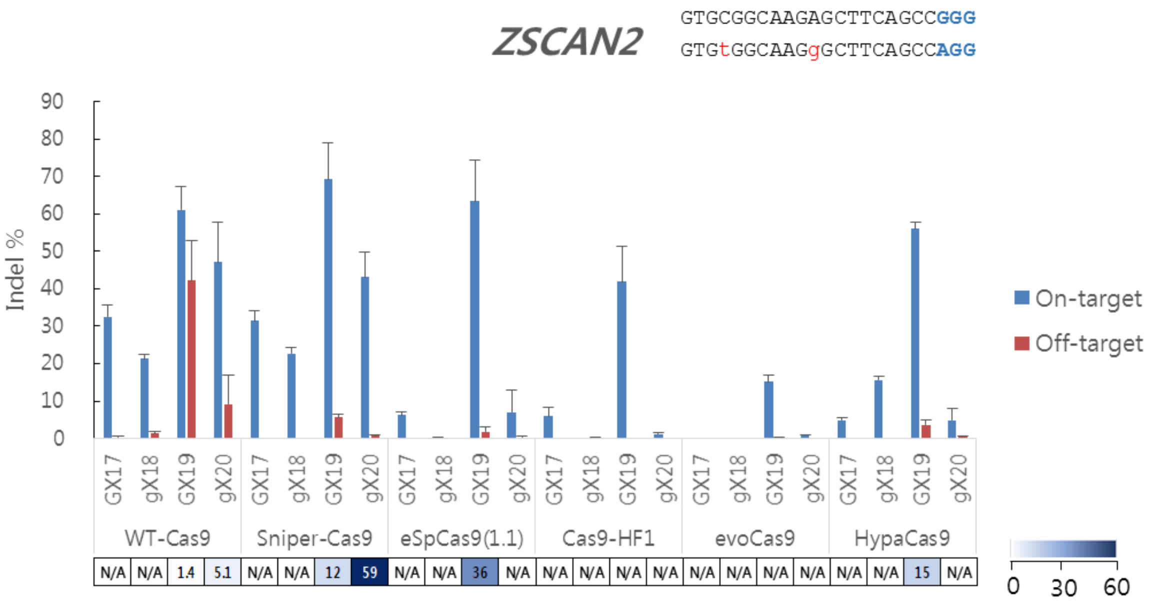
ZSCAN2
GTGCGGCAAGAGCTTCAGCCGGG
GTGtGGCAAGgGCTTCAGCCAGG
Indel %
On-target
Off-target
GX17 gX18 GX19 gX20
WT-Cas9
Sniper-Cas9
eSpCas9(1.1)
Cas9-HF1
evoCas9
HypaCas9
N/A N/A 1.4 5.1 N/A N/A 12 59 N/A N/A 36 N/A N/A N/A N/A N/A N/A N/A N/A N/A N/A N/A 15 N/A
0 30 60

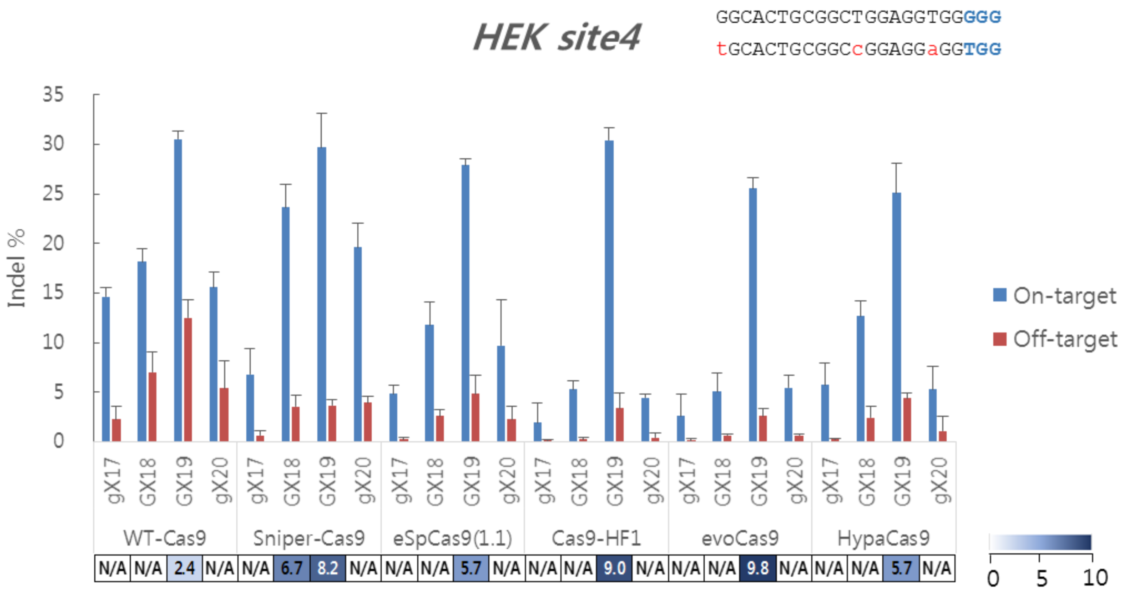
HEK site4
GGCACTGCGGCTGGAGGTGGGGG
tGCACTGCGGCcGGAGGaGGTGG
Indel %
On-target
Off-target
gX17 GX18 GX19 gX20
WT-Cas9
Sniper-Cas9
eSpCas9(1.1)
Cas9-HF1
evoCas9
HypaCas9
N/A N/A 2.4 N/A N/A 6.7 8.2 N/A N/A N/A 5.7 N/A N/A N/A 9.0 N/A N/A N/A 9.8 N/A N/A N/A 5.7 N/A
0 5 10

Supplementary Figure 9.

Western blot was performed to check the protein expression level of Cas9 variants in HEK293T cells. Cas9 proteins were detected by HA tag. GAPDH were control.

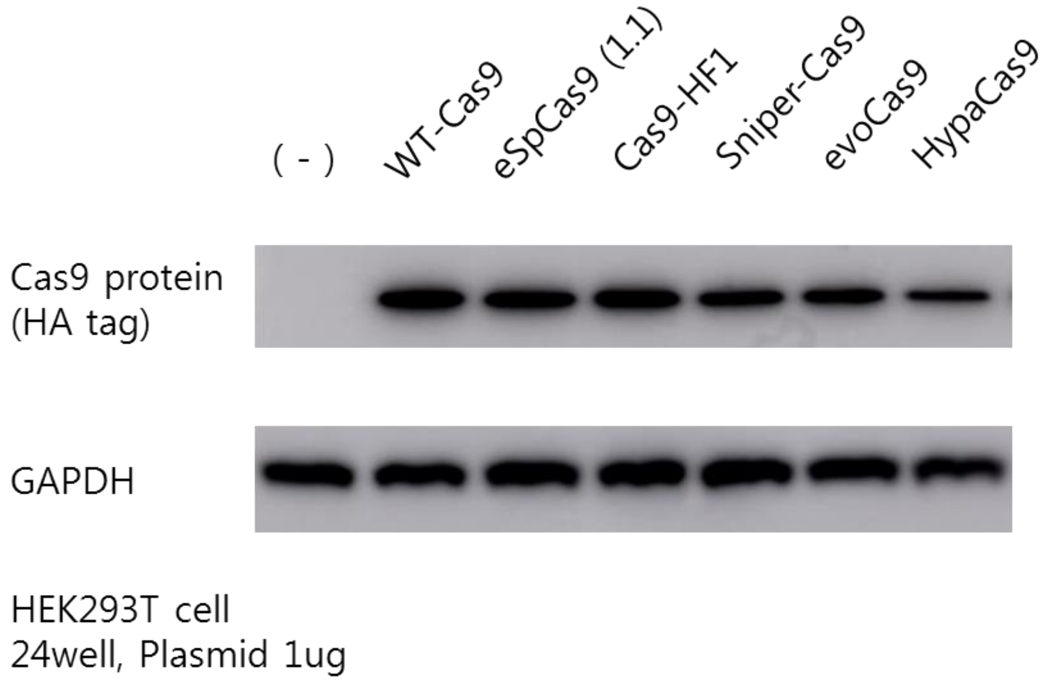

Supplementary Figure 10.

On-target and off-target activities were measured in HeLa cells using the same sites tested in HEK293T cells. Four different targets were tested. Indel frequencies were measured using targeted deep sequencing. Error bars indicate s.e.m. (n = 3)

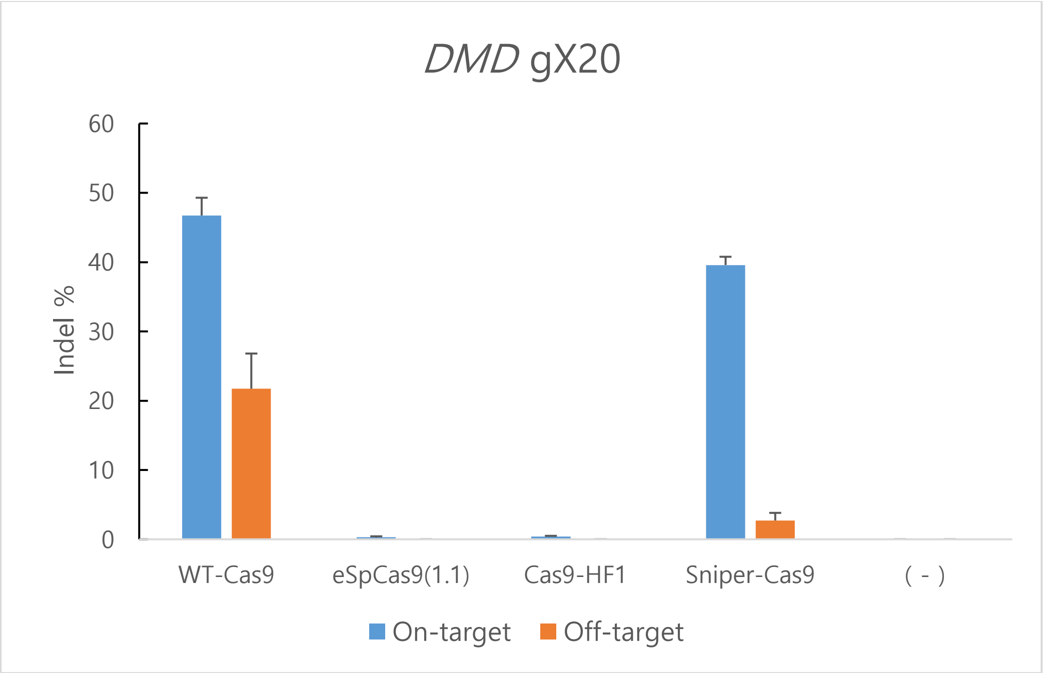

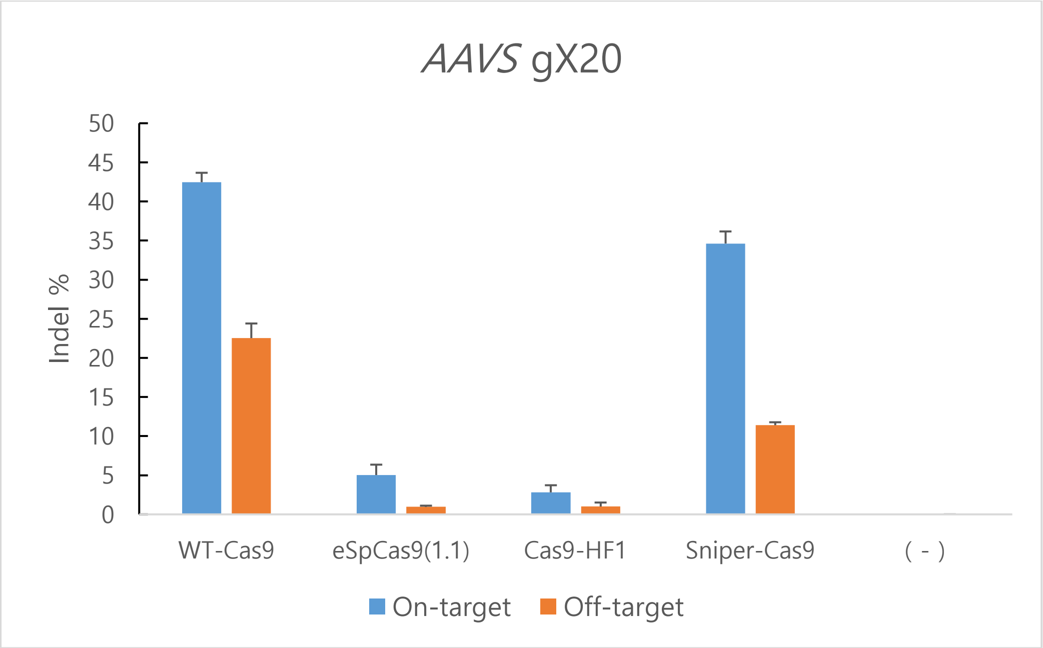

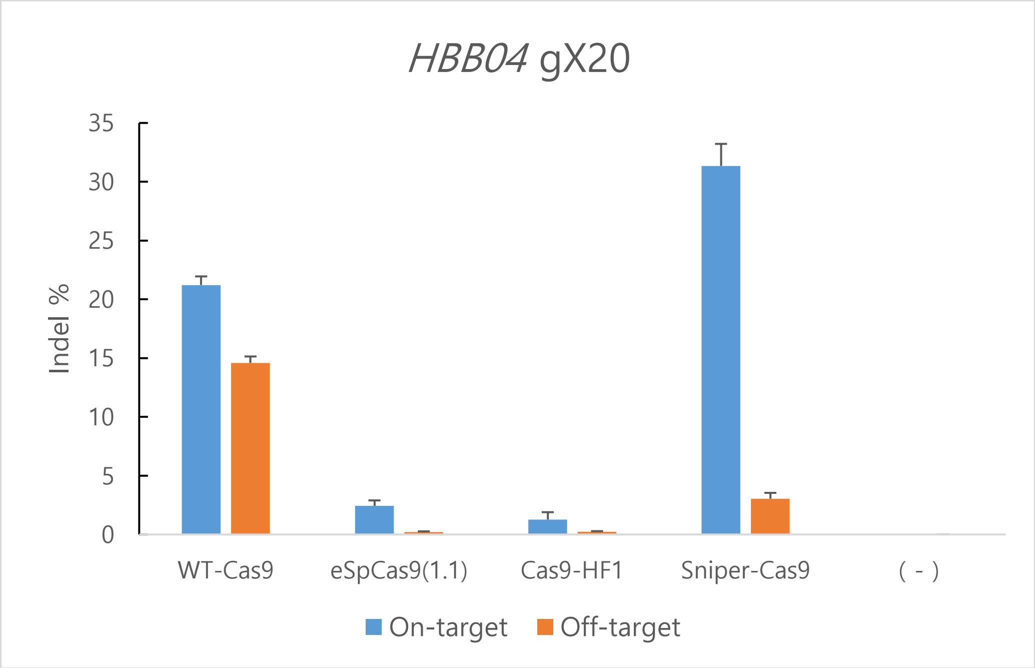
HBB04 gX20
35
30
25
20
15
10
5
0
Indel %
WT-Cas9
eSpCas9(1.1)
Cas9-HF1
Sniper-Cas9
(-)
On-target
Off-target

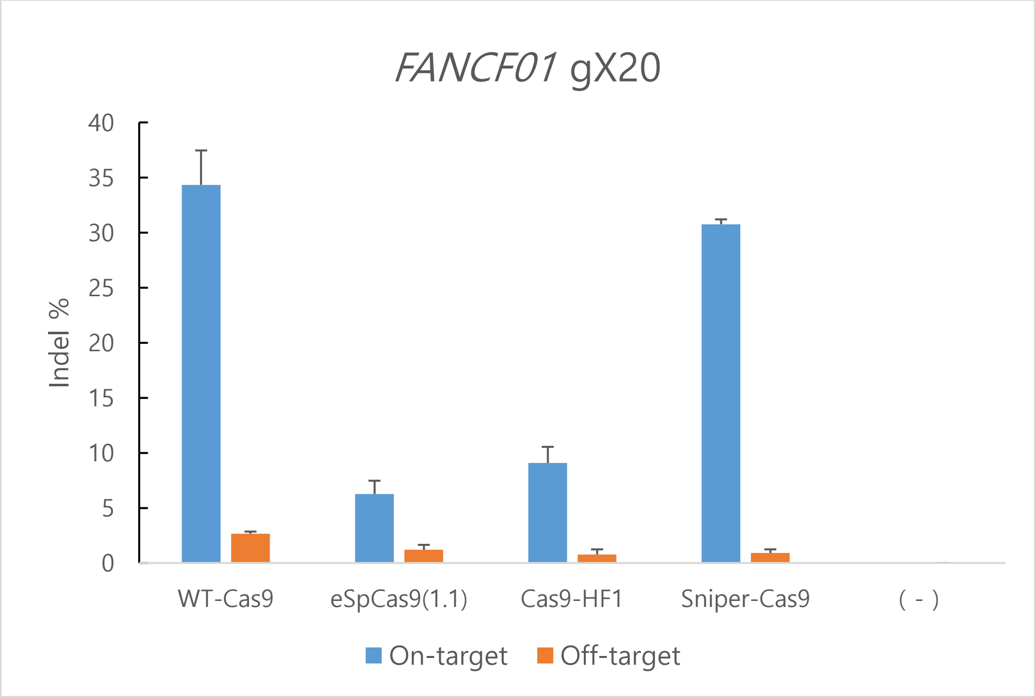
FANCF01 gX20
40
35
30
25
20
15
10
5
0
Indel %
WT-Cas9
eSpCas9(1.1)
Cas9-HF1
Sniper-Cas9
(-)
On-target
Off-target

Supplementary Figure 11.

Tolerance of Cas9 variants for sgRNAs containing mismatches relative to *HBB02*, *VEGFA* and *FANCF01* targets. *HBB02* was targeted with gX19 sgRNAs and *VEGFA* was targeted with gX20 sgRNAs. *FANCF01* was targeted with 2bp mismatched GX19 sgRNAs. Indel frequencies were measured using targeted deep sequencing. Error bars indicate s.e.m. (n = 3)

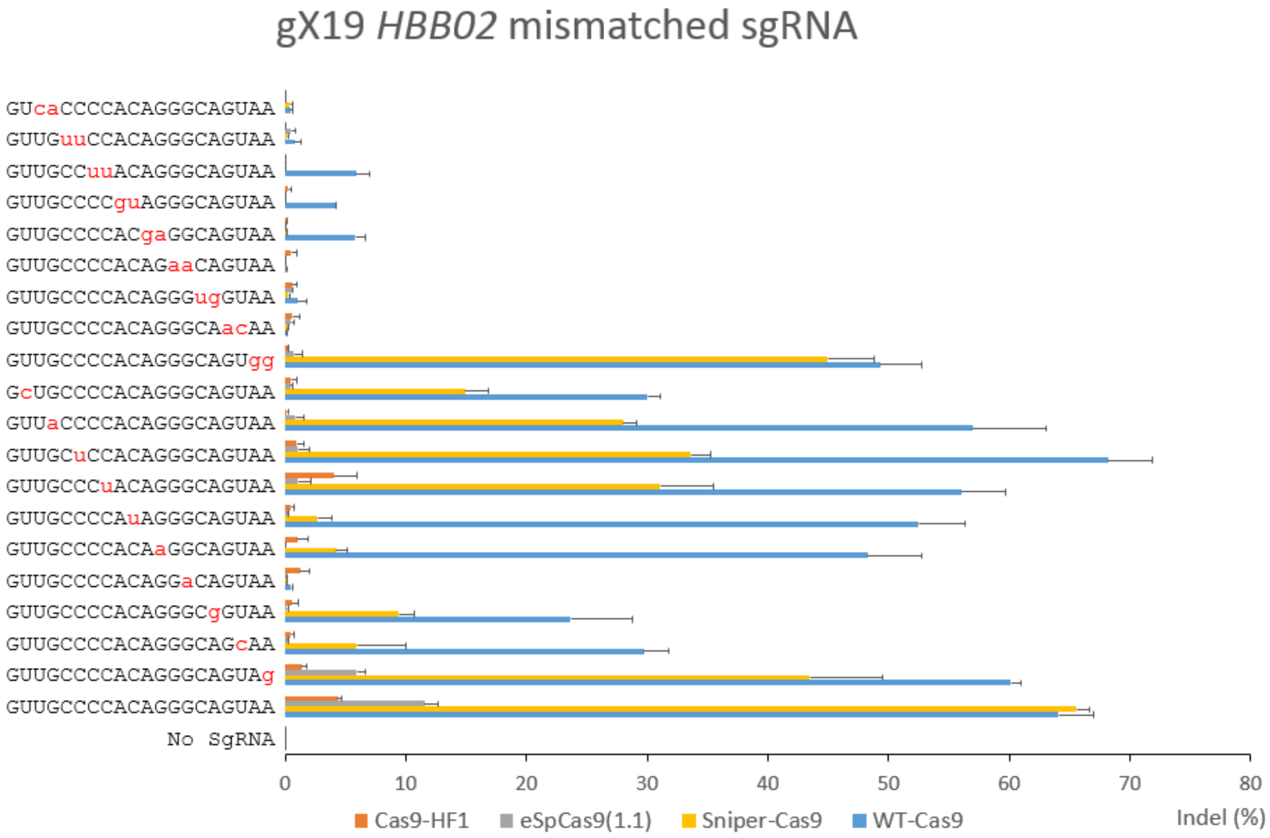

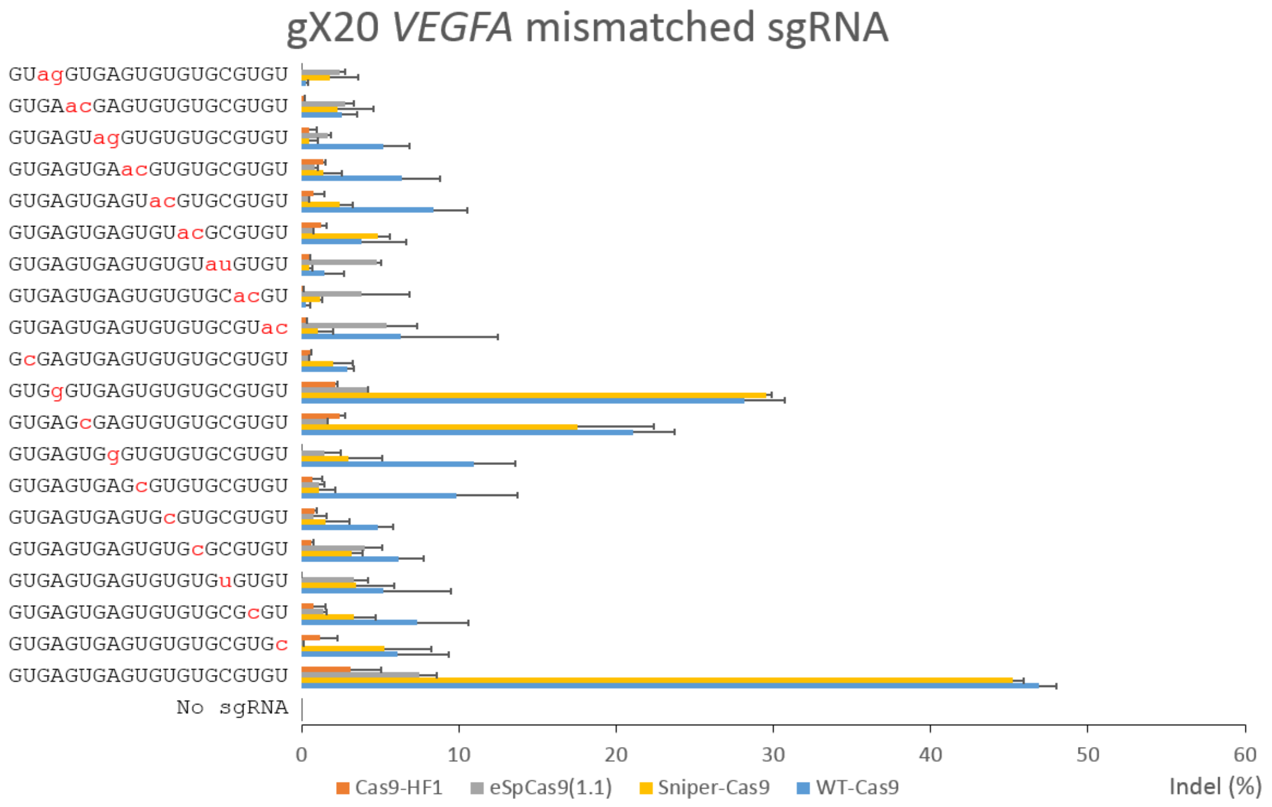
gX20 VEGFA mismatched sgRNA
GUagGUGAGUGUGUGCGUGU
GUGAacGAGUGUGUGCGUGU
GUGAGUagGUGUGUGCGUGU
GUGAGUGAacGUGUGCGUGU
GUGAGUGAGUacGUGCGUGU
GUGAGUGAGUGUacGCGUGU
GUGAGUGAGUGUGUauGUGU
GUGAGUGAGUGUGUGCacGU
GUGAGUGAGUGUGUGCGUac
GcGAGUGAGUGUGUGCGUGU
GUGgGUGAGUGUGUGCGUGU
GUGAGcGAGUGUGUGCGUGU
GUGAGUGgGUGUGUGCGUGU
GUGAGUGAGcGUGUGCGUGU
GUGAGUGAGUGcGUGCGUGU
GUGAGUGAGUGUGcGCGUGU
GUGAGUGAGUGUGUGuGUGU
GUGAGUGAGUGUGUGCGcGU
GUGAGUGAGUGUGUGCGUGc
GUGAGUGAGUGUGUGCGUGU
No sgRNA
0
10
20
30
40
50
60
Cas9-HF1
eSpCas9(1.1)
Sniper-Cas9
WT-Cas9
Indel (%)

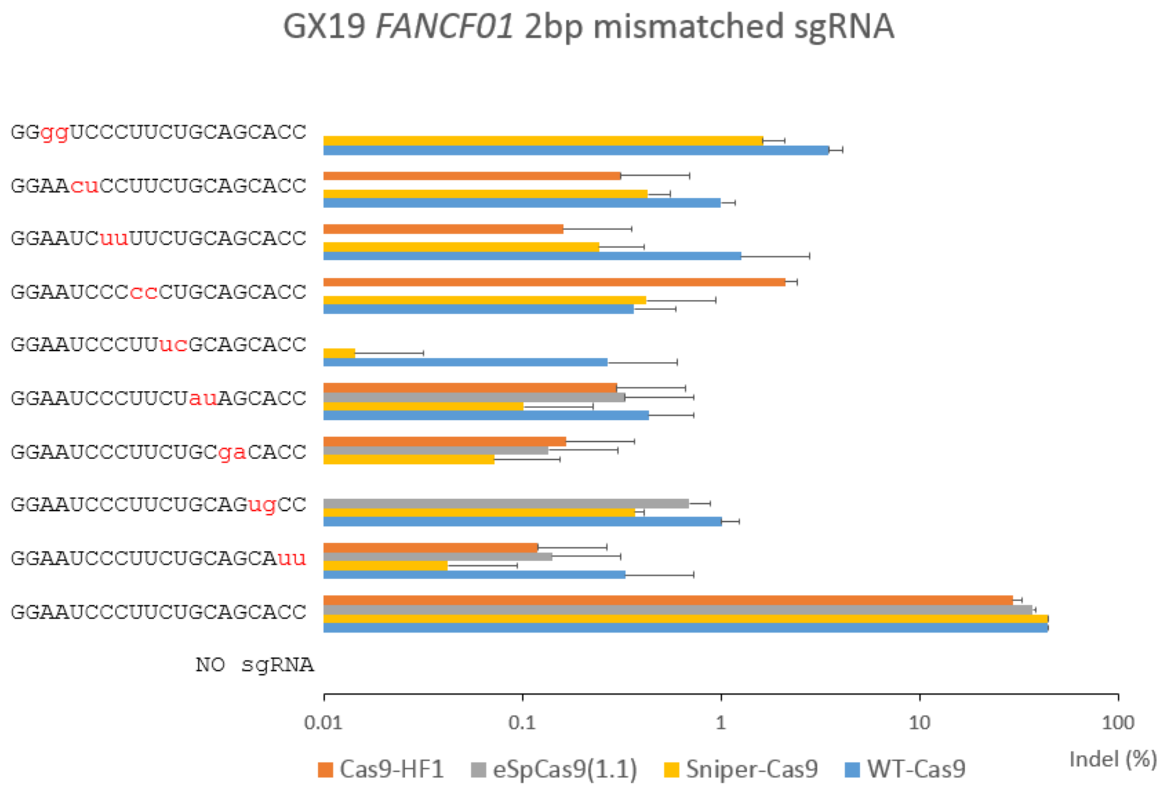
GX19 FANCF01 2bp mismatched sgRNA
GGggUCCCUUCUGCAGCACC
GGAAcuCCUUCUGCAGCACC
GGAAUCuuUUCUGCAGCACC
GGAAUCCCccCUGCAGCACC
GGAAUCCCUUucGCAGCACC
GGAAUCCCUUCUauAGCACC
GGAAUCCCUUCUGCgaCACC
GGAAUCCCUUCUGCAGugCC
GGAAUCCCUUCUGCAGCAuu
GGAAUCCCUUCUGCAGCACC
NO sgRNA
0.01
0.1
1
10
100
Cas9-HF1
eSpCas9(1.1)
Sniper-Cas9
WT-Cas9
Indel (%)

Supplementary Figure 12.

On-target and off-target activities of xCas9-3.7 compared to WT-Cas9 and Sniper-Cas9 using sgRNAs targeting 10 target sites. Specificity ratios were determined by dividing indel frequencies at on-target sites by those at respective off-target sites. sgRNAs with a matched guanine at the 5' terminus (GX19) and those with a mismatched guanine (gX19 or gX20) are indicated. N/A (Not Available): Specificity ratios were not calculated when on-target activities were less than 70% of the WT plus 20mer guide sequence. Error bars indicate s.e.m. (*n*=3).

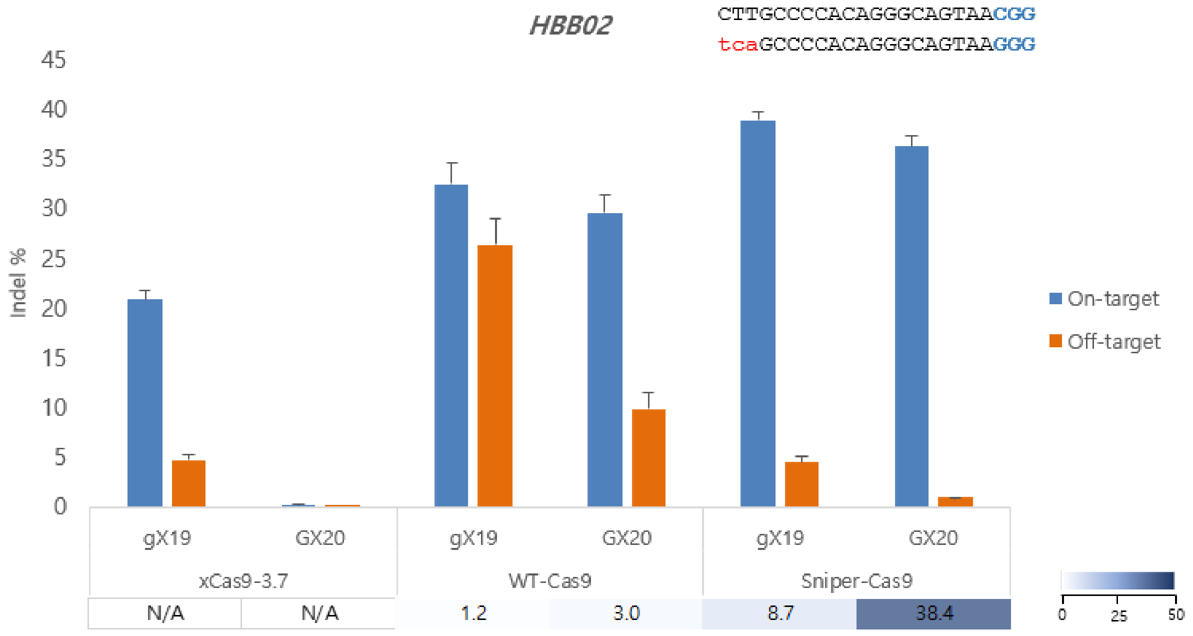

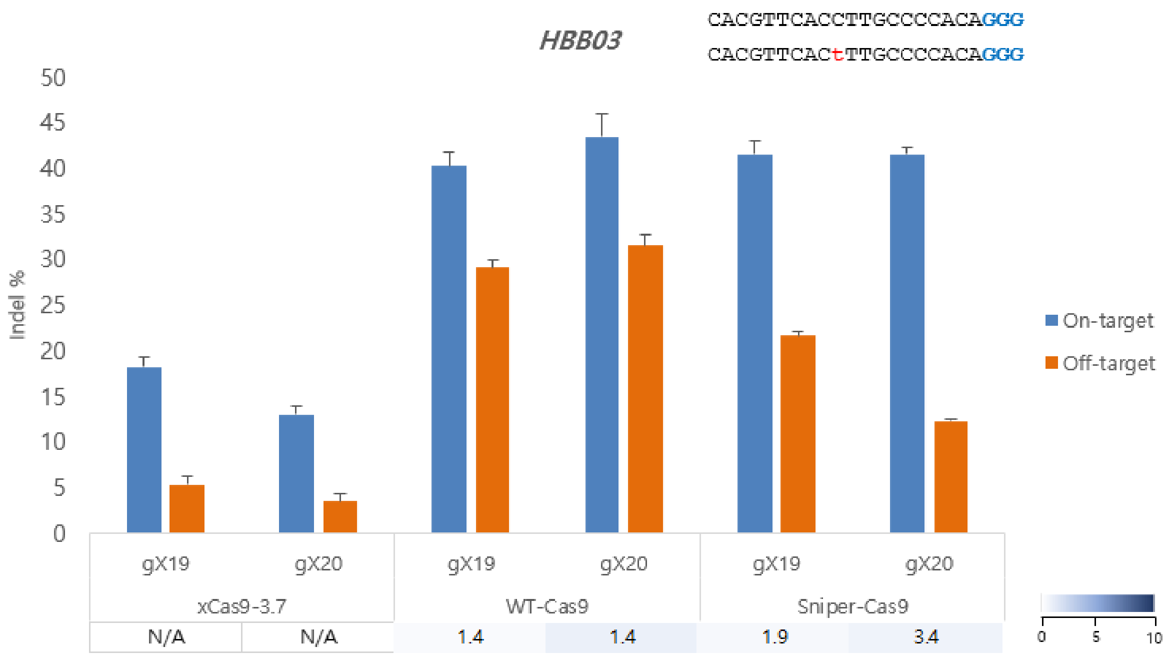
HBB03
CACGTTCACCTTGCCCCACAGGG
CACGTTCACtTTGCCCCACAGGG
Indel %
50
45
40
35
30
25
20
15
10
5
0
On-target
Off-target
gX19
gX20
gX19
gX20
gX19
gX20
xCas9-3.7
WT-Cas9
Sniper-Cas9
N/A
N/A
1.4
1.4
1.9
3.4
0
5
10

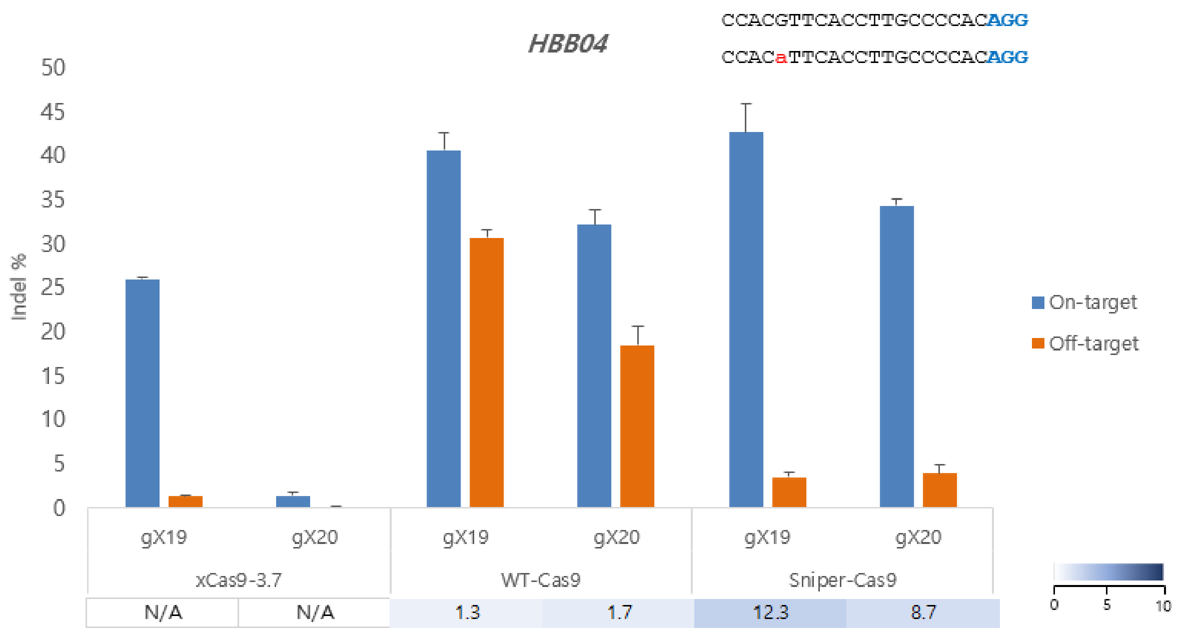
HBB04
CCACGTTCACCTTGCCCCACAGG
CCACaTTCACCTTGCCCCACAGG
Indel %
50
45
40
35
30
25
20
15
10
5
0
On-target
Off-target
gX19
gX20
gX19
gX20
gX19
gX20
xCas9-3.7
WT-Cas9
Sniper-Cas9
N/A
N/A
1.3
1.7
12.3
8.7
0
5
10

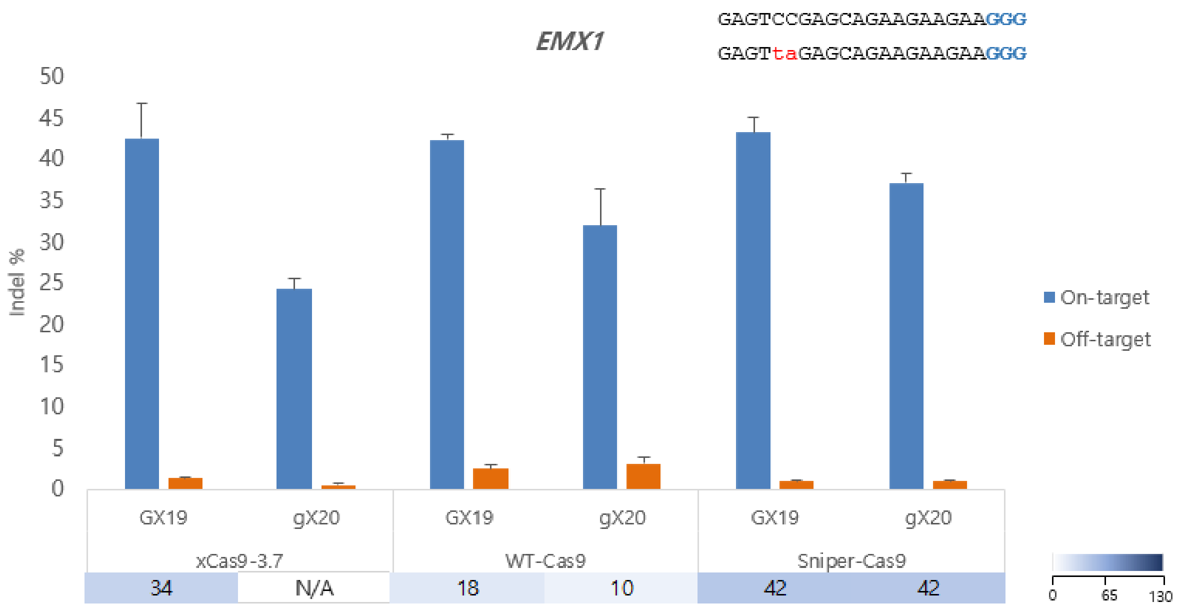
EMX1
GAGTCCGAGCAGAAGAAGAAGGG
GAGTtaGAGCAGAAGAAGAAGGG
Indel %
50
45
40
35
30
25
20
15
10
5
0
GX19
gX20
GX19
gX20
GX19
gX20
xCas9-3.7
WT-Cas9
Sniper-Cas9
34
N/A
18
10
42
42
On-target
Off-target
0
65
130

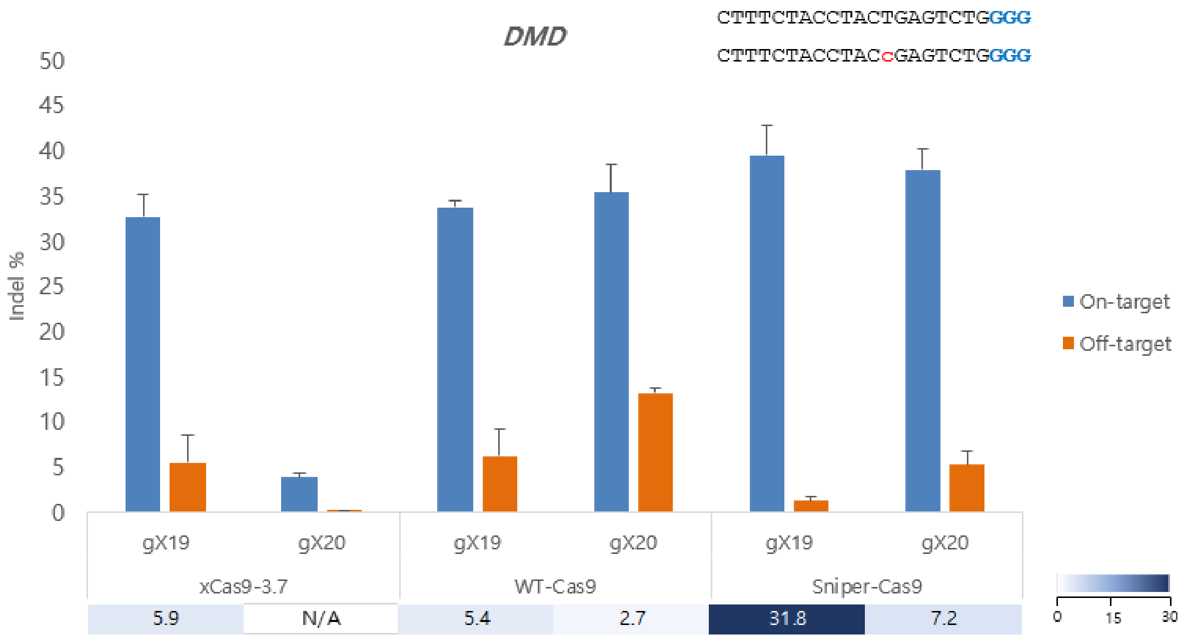
DMD
CTTTCTACCTACTGAGTCTGGGG
CTTTCTACCTACcGAGTCTGGGG
Indel %
50
45
40
35
30
25
20
15
10
5
0
gX19
gX20
gX19
gX20
gX19
gX20
xCas9-3.7
WT-Cas9
Sniper-Cas9
5.9
N/A
5.4
2.7
31.8
7.2
On-target
Off-target
0
15
30

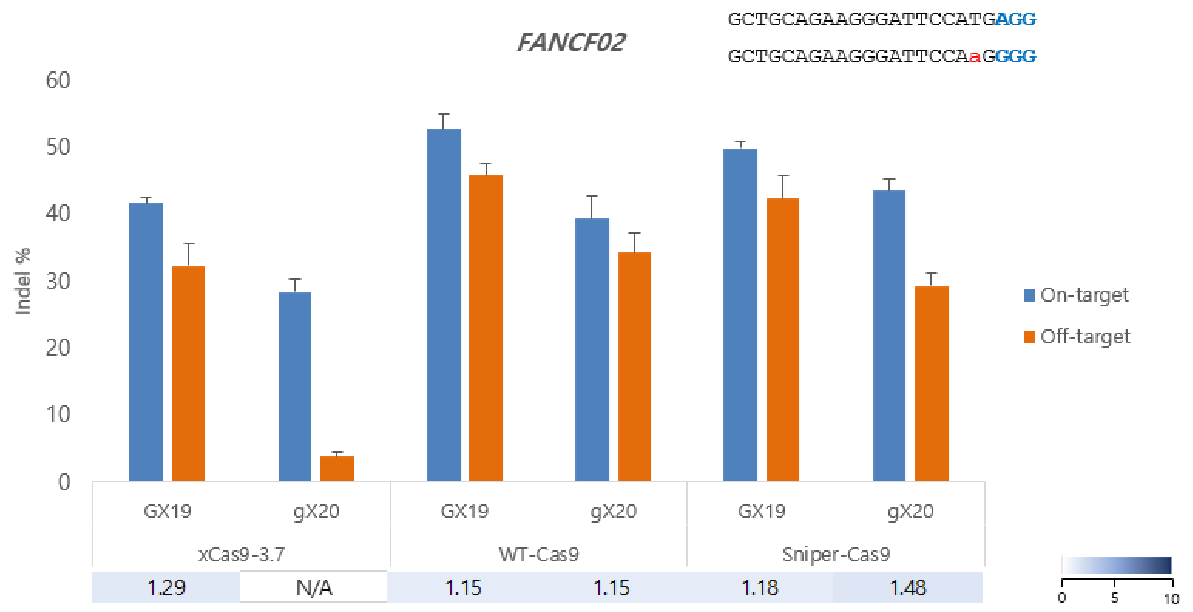

FANCF02
GCTGCAGAAGGGATTCCATGAGG
GCTGCAGAAGGGATTCCAaGGGG
Indel %
60
50
40
30
20
10
0
On-target
Off-target
GX19
gX20
GX19
gX20
GX19
gX20
xCas9-3.7
WT-Cas9
Sniper-Cas9
1.29
N/A
1.15
1.15
1.18
1.48
0
5
10

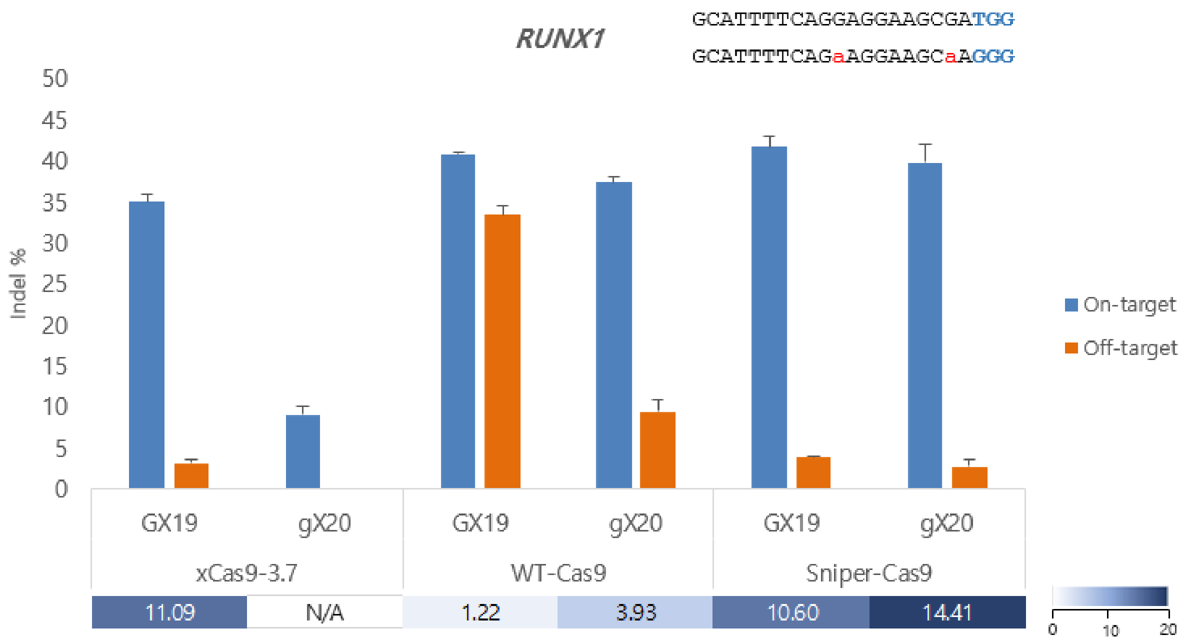

RUNX1
GCATTTTCAGGAGGAAGCGATGG
GCATTTTCAGaAGGAAGCaAGGG
Indel %
50
45
40
35
30
25
20
15
10
5
0
On-target
Off-target
GX19
gX20
GX19
gX20
GX19
gX20
xCas9-3.7
WT-Cas9
Sniper-Cas9
11.09
N/A
1.22
3.93
10.60
14.41
0
10
20

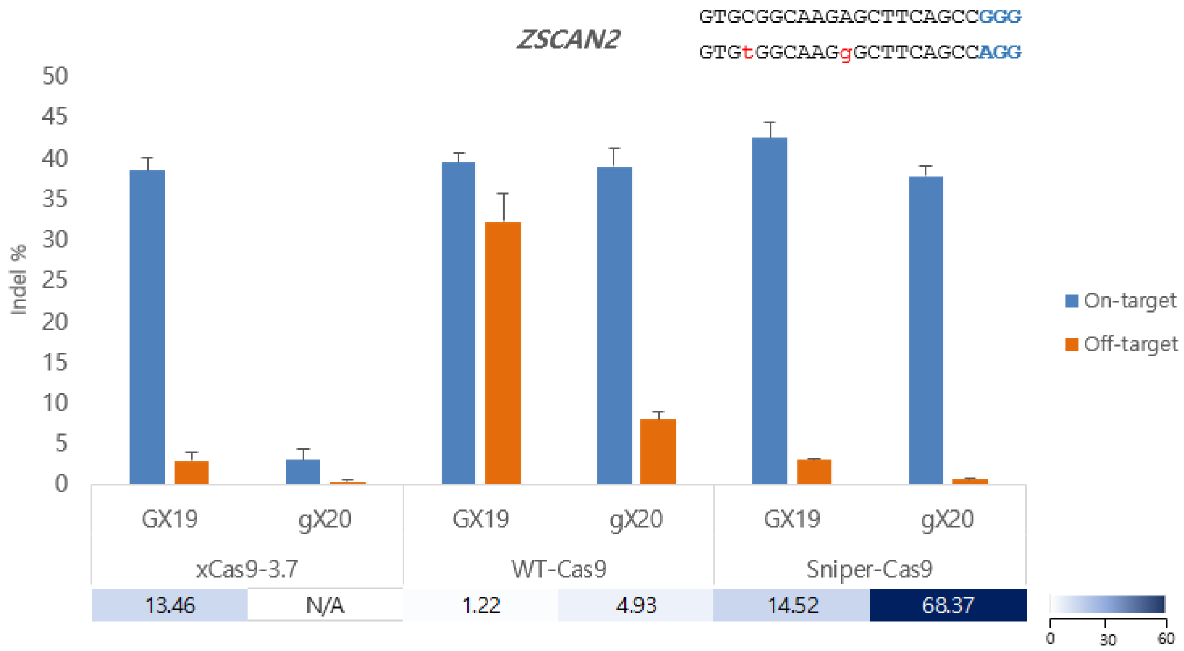
ZSCAN2
GTGCGGCAAGAGCTTCAGCCGGG
GTGtGGCAAGgGCTTCAGCCAGG
Indel %
50
45
40
35
30
25
20
15
10
5
0
On-target
Off-target
GX19
gX20
GX19
gX20
GX19
gX20
xCas9-3.7
WT-Cas9
Sniper-Cas9
13.46
N/A
1.22
4.93
14.52
68.37
0
30
60

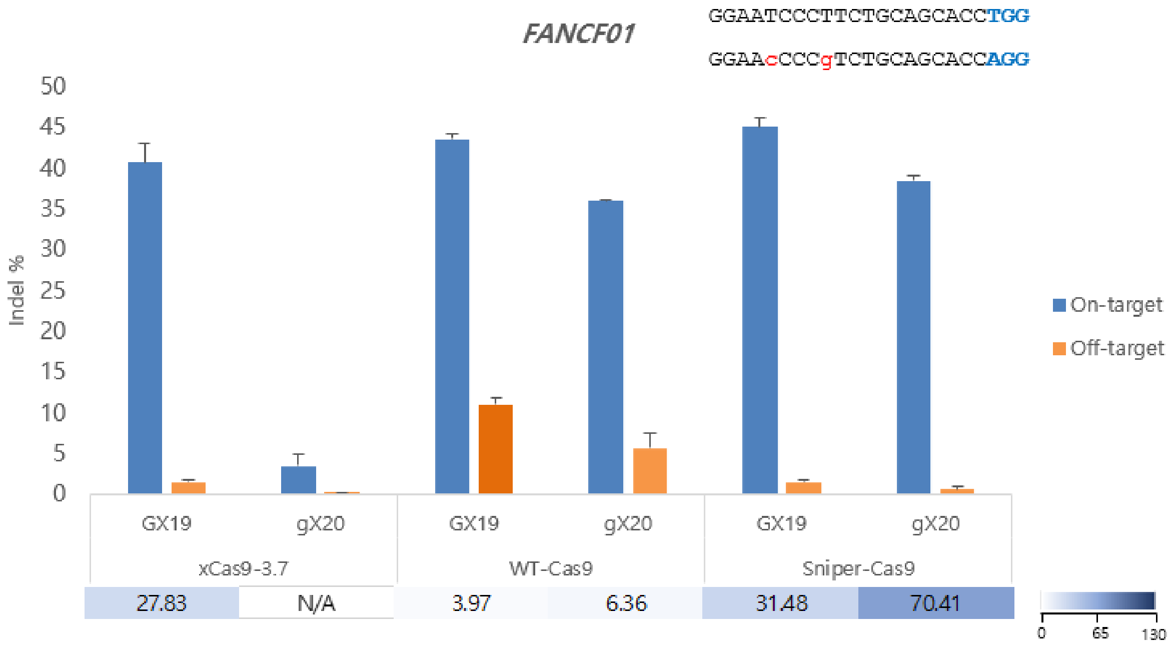
FANCF01
GGAATCCCTTCTGCAGCACCTGG
GGAAcCCCgTCTGCAGCACCAGG
Indel %
50
45
40
35
30
25
20
15
10
5
0
On-target
Off-target
GX19
gX20
GX19
gX20
GX19
gX20
xCas9-3.7
WT-Cas9
Sniper-Cas9
27.83
N/A
3.97
6.36
31.48
70.41
0
65
130

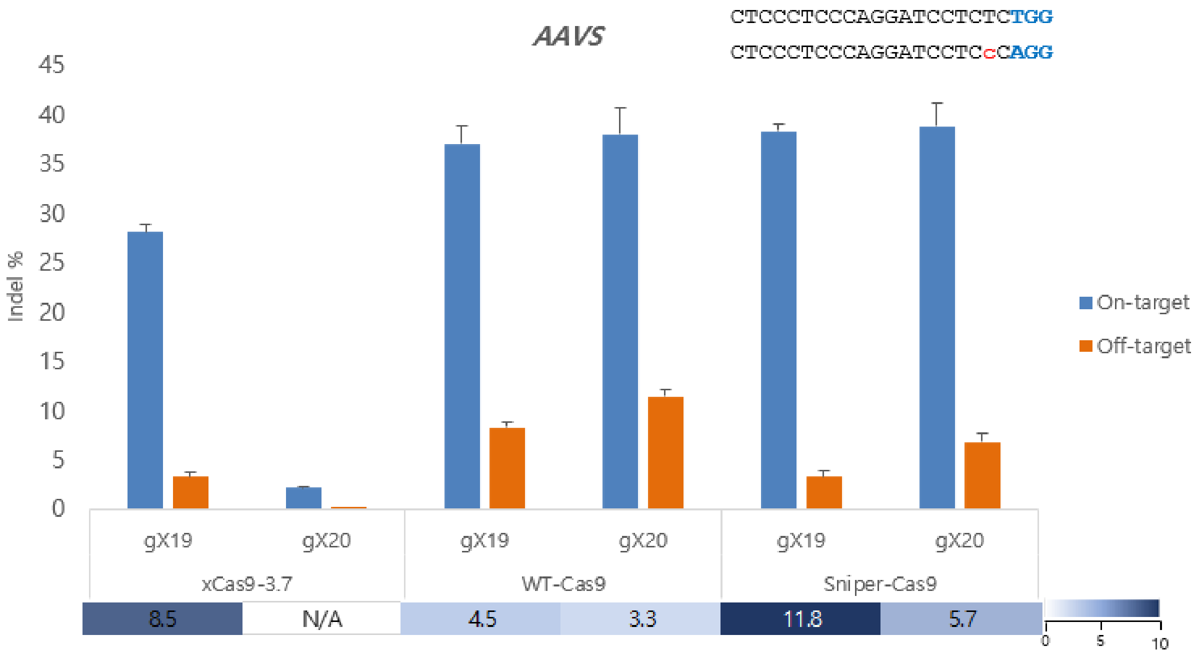
AAVS
CTCCCTCCCAGGATCCTCTCTGG
CTCCCTCCCAGGATCCTCcCAGG
Indel %
45
40
35
30
25
20
15
10
5
0
On-target
Off-target
gX19
gX20
gX19
gX20
gX19
gX20
xCas9-3.7
WT-Cas9
Sniper-Cas9
8.5
N/A
4.5
3.3
11.8
5.7
0
5
10

Supplementary Figure 13.

Validation of 10 candidate off-target sites for WT-Cas9 and Sniper-Cas9 targeting *AAVS, DMD, FANCF01* and *HBB04*. Candidate sites had received the top rank of cleavage score in Digenome-sequencing experiments and included fewer than 5 mismatches relative to the on-target site.

AAVS
WT-Cas9

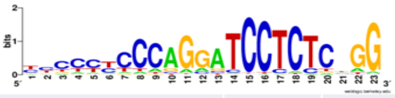

tcCCaTCCCtGGATCCTCTCaaG	4	58.53207
CTtCCatCCAGGAcCCTCTCTGG	4	48.09723
CaCCtTCCCAGGgTCCTCTtcGG	4	38.65975
gTCCCTCaCAcaATCCTCTCTGG	4	38.57542
CaCCtTCCCAGGATCaTCcCcGG	4	29.14423
gaCCCTCCaAGGAcCCTCTCcGG	4	27.21651
CTCCCTCCCAGGATCCTCcCaGG	1	26.63057
CagCaTCCCAGGATCCTCTtTGG	4	25.70335
gTCCCTCCCAGGAcCCTCTgTGG	3	25.64998
CTttgTCCCAGGATCCTCTggGG	4	22.09995
CTCCCaCCCAGGAcCCTCTCTGG	2	20.42314

AAVS
Sniper-Cas9

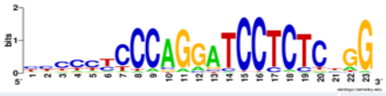

tcCCaTCCCtGGATCCTCTCaaG	4	41.77622
CTtCCatCCAGGAcCCTCTCTGG	4	32.57071
CTCCCTCCCAGGATCCTCcCaGG	1	29.3543
CaCCtTCCCAGGgTCCTCTtcGG	4	29.23651
CTCCCaCCCAGGAcCCTCTCTGG	2	27.99266
CaCCtTCCCAGGATCaTCcCcGG	4	26.97908
gTCCCTCaCAcaATCCTCTCTGG	4	26.90956
CagCaTCCCAGGATCCTCTtTGG	4	22.76564
CTttgTCCCAGGATCCTCTggGG	4	22.30188
gaCCCTCCaAGGAcCCTCTCcGG	4	21.87487
gTCCCTCCCAGGAcCCTCTgTGG	3	20.27001

DMD
WT-Cas9

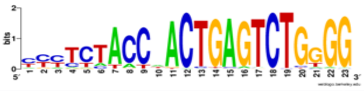

CTcTCTACCaACTGAGTCTGGGG	2	23.04014
CTTTCTACCTACcGAGTCTGGGG	1	18.53525
CTcTCTAtCTACTGAGTCTGGGG	2	15.02185
accTCTACCaACTGAGTCTGGGG	4	11.03111
tcTTCTACCaACTGAGTCTGGGG	3	8.952455
tTTTCTACCaACTGAGTCTGGGG	2	7.939596
CTgTCTACCcACTGAGTCTGGGG	2	7.855161
CcTTCTACCaACTGAGTCTGGGG	2	7.660574
CTcTCTACCTACTGAGTCTGcaG	1	4.872062

DMD
Sniper-Cas9

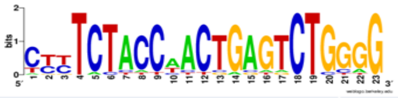

CTcTCTACCaACTGAGTCTGGGG	2	16.38587
CTTTCTACCTACcGAGTCTGGGG	1	12.13051
CcTTCTACCaACTGAGTCTGGGG	2	6.822693
tcTTCTACCaACTGAGTCTGGGG	3	5.840074
CTgTCTACCcACTGAGTCTGGGG	2	5.134188
tTTTCTACCaACTGAGTCTGGGG	2	4.270272
accTCTACCaACTGAGTCTGGGG	4	3.812088
CTcTCTAtCTACTGAGTCTGGGG	2	3.744405
CTcTCTACCTACTGAGTCTGcaG	1	3.146989

FANCF01
WT-Cas9

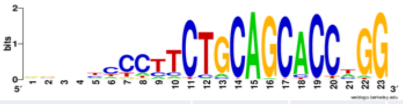

tGAATCCCaTCTcCAGCACCaGG	3	37.71296
GGgAgtCCaTCTGCAGCACCaGG	4	28.99838
GGAgTCCCTcCTaCAGCACCaGG	3	23.52006
GGAAcCCCgTCTGCAGCACCaGG	2	18.93554
GGAgcCCCTTCTGCAG-ACCcGG	3	16.50595
aGctcCCaTTCTGCAGCACCcGG	5	14.33861
aGggTCCCTTCTGCAGCcCCTGG	4	12.63617
aGttcCCCaTCTGCAGCACCaGG	5	10.36073
aGAggCCCcTCTGCAGCACCaGG	4	9.442236
GGAAcaCCTTCTGCAGCtCCaGG	3	6.181714

FANCF01
Sniper-Cas9

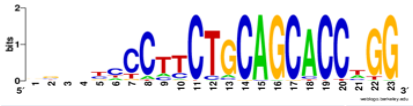

GGAgcCCCTTCTGCAG-ACCcGG	3	29.31338
GGAgTCCCTcCTaCAGCACCaGG	3	19.92436
tGAATCCCaTCTcCAGCACCaGG	3	17.98515
aGggTCCCTTCTGCAGCcCCTGG	4	11.5206
GGAAcaCCTTCTGCAGCtCCaGG	3	8.993105
GGgAgtCCaTCTGCAGCACCaGG	4	8.789375
GGAAcCCCgTCTGCAGCACCaGG	2	8.772959
aGctcCCaTTCTGCAGCACCcGG	5	7.504666
aGttcCCCaTCTGCAGCACCaGG	5	6.829126
aGAggCCCcTCTGCAGCACCaGG	4	6.717098

HBB04
WT-Cas9

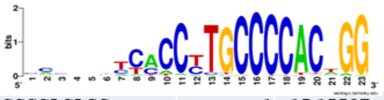

CCACGTTCACtTTGCCCCACAGG	1	15.15537
gCACGcTCACCcTGCCC-ACgGG	4	13.39999
CCcaGcTCACCcTGCCCCACtGG	4	10.88308
CCctGcTCACCcTGCCCCACtGG	4	9.297476
CtgaGgTCACCTTGCCCCACtGG	4	8.148633
CagaaTTCACCcTGCCCCACgGG	5	7.807143
gCAgGTTCcaCcTGCCCCACtGG	5	7.774244
CCcCaaaCACCTTGCCCCACtGG	4	7.65154
gaAtGcTCACCTTGCCCCACAGG	4	6.915867
gCtCcTcCACCTTGCCCCtCgGG	5	6.099295

HBB04
Sniper-Cas9

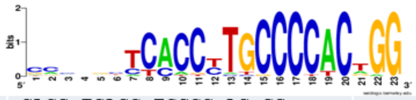

gCACGcTCACCcTGCCC-ACgGG	4	14.45391
CCACGTTCACtTTGCCCCACAGG	1	8.69193
CCctGcTCACCcTGCCCCACtGG	4	7.441092
gCAgGTTCcaCcTGCCCCACtGG	5	6.970092
CagaaTTCACCcTGCCCCACgGG	5	6.255218
gCtCcTcCACCTTGCCCCtCgGG	5	4.892282
gaAtGcTCACCTTGCCCCACAGG	4	4.451276
CCcaGcTCACCcTGCCCCACtGG	4	4.29183
CtgaGgTCACCTTGCCCCACtGG	4	3.654234
CCcCaaaCACCTTGCCCCACtGG	4	3.43091

Supplementary Figure 14.

WT-Cas9 and Sniper-Cas9 off-target sites for *AAVS, DMD* and *HBB04* validated in HEK293T cells by targeted deep sequencing. Error bars indicate s.e.m. (*n*=3).

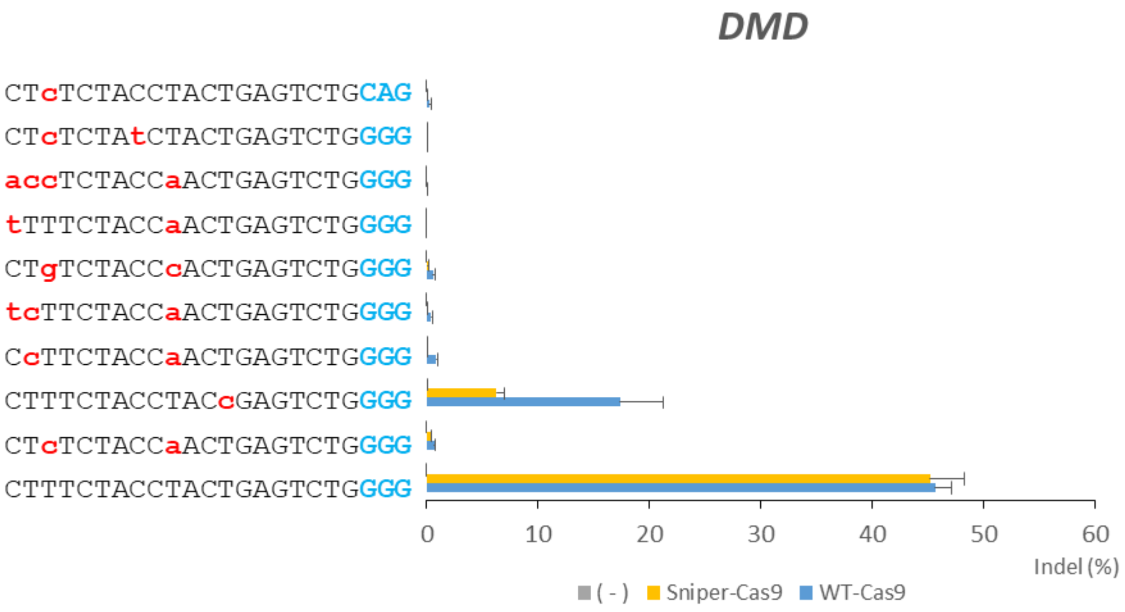

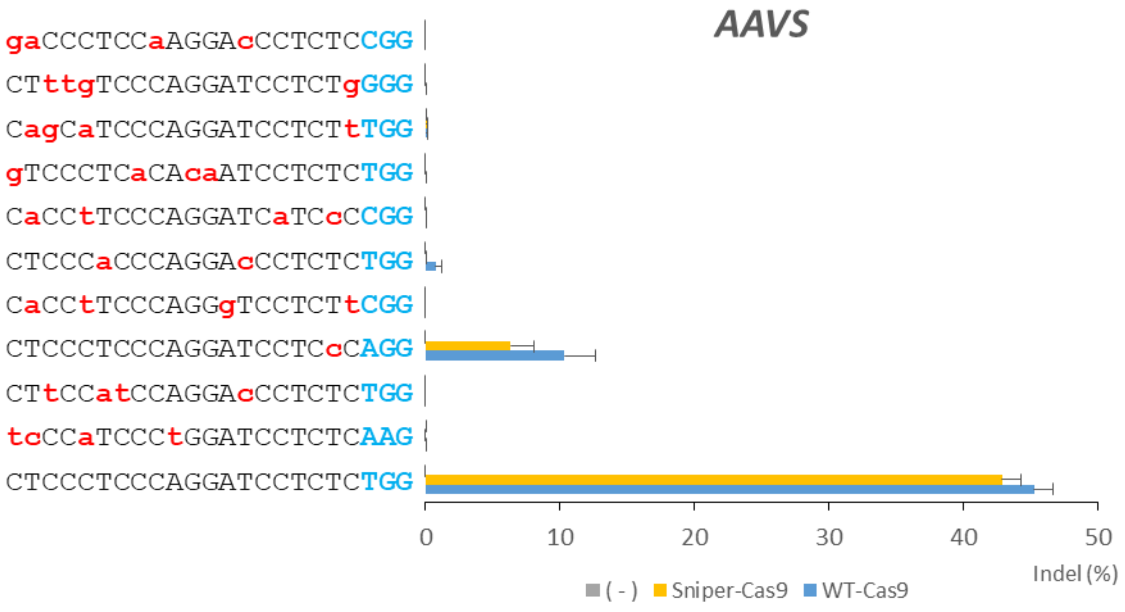

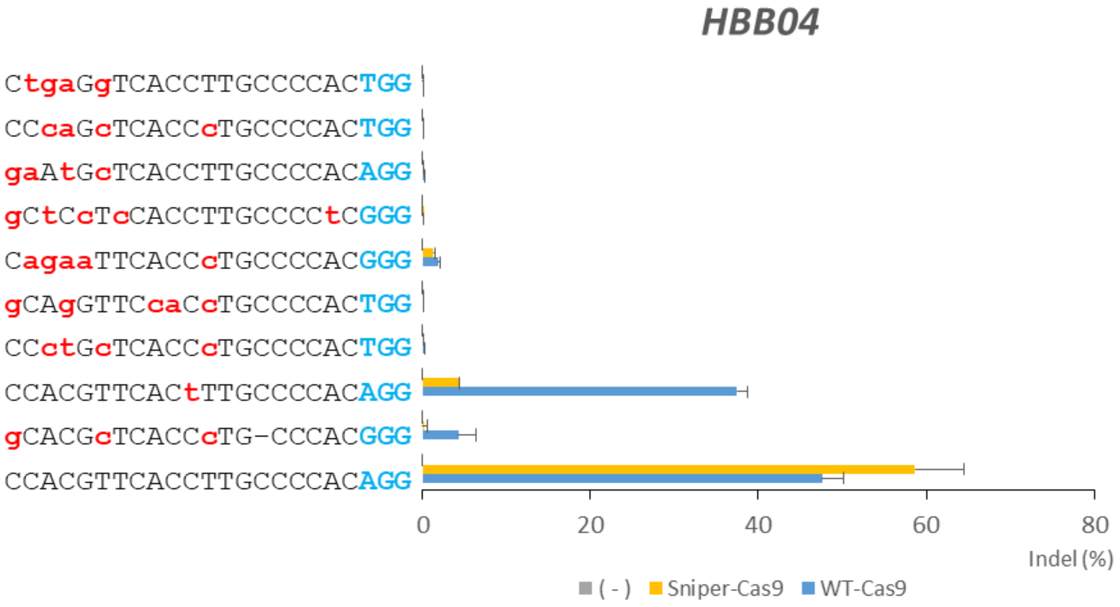

HBB04
CtgaGgTCACCTTGCCCCACTGG
CCcaGcTCACCcTGCCCCACTGG
gaAtGcTCACCTTGCCCCACAGG
gCtCcTcCACCTTGCCCCtCGGG
CagaaTTCACCcTGCCCCACGGG
gCAgGTTCcaCcTGCCCCACTGG
CCctGcTCACCcTGCCCCACTGG
CCACGTTCACtTTGCCCCACAGG
gCACGcTCACCcTG-CCCACGGG
CCACGTTCACCTTGCCCCACAGG
0
20
40
60
80
Indel (%)
(-)
Sniper-Cas9
WT-Cas9

Supplementary Figure 15.

Validation of off-targets candidate from only Sniper-Cas9 in Digenome-seq. Off-target sites for *AAVS, DMD* and *FANC01* validated in HEK293T cells by targeted deep sequencing. Error bars indicate s.e.m. (n=3)

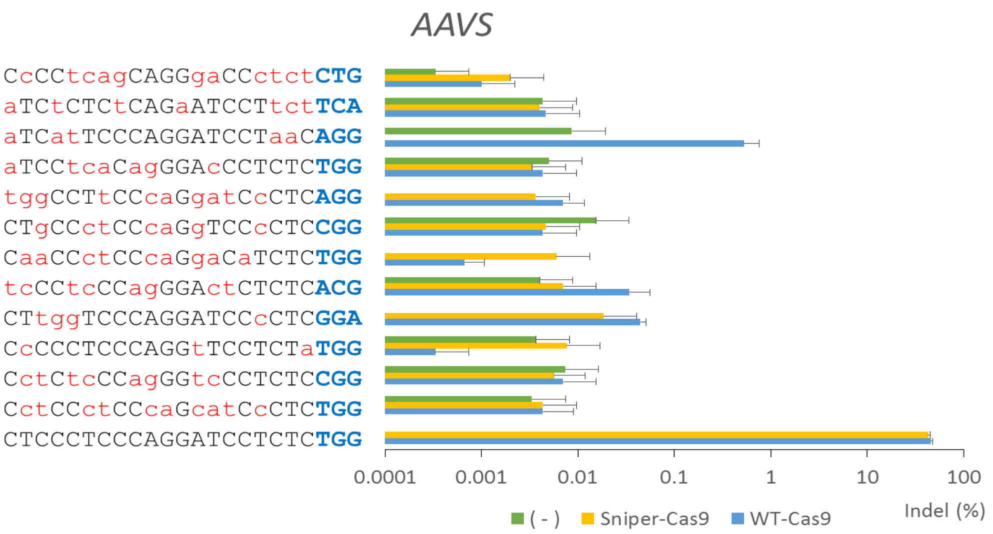

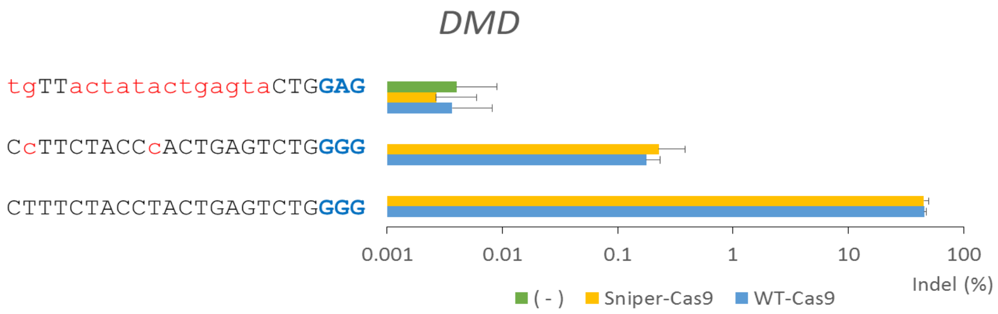

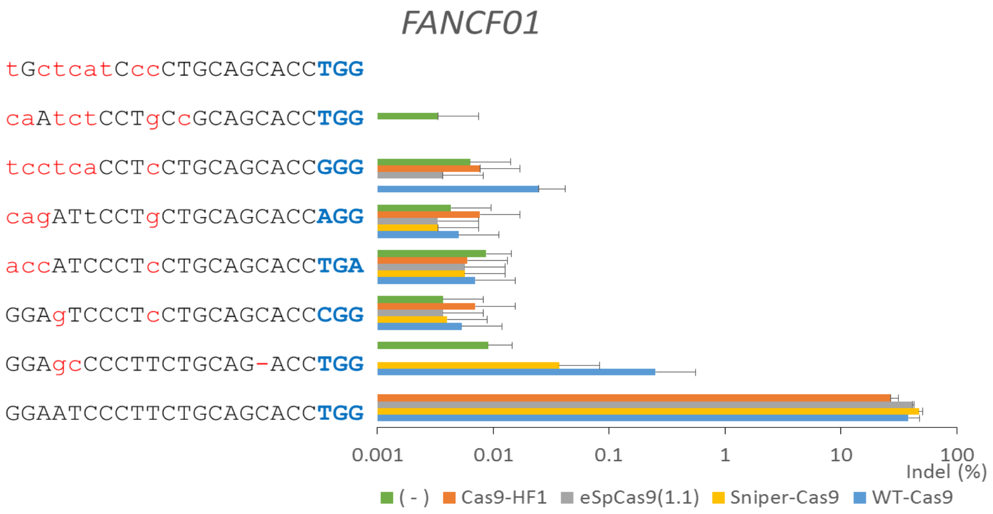

Supplementary Figure 16.

Validation of off-targets candidate (Yellow mark) from Guide-seq from other studies. 70% of WT on-target activity level was represented by red line. Off-target sites for *EMX1 site 2, FANCF3, RUNX1, ZSCAN2 and DNMT1 site 4* were validated in HEK293T cells by targeted deep sequencing. Error bars indicate s.e.m. (*n*=3)

	# targets for specificity ratio comparison	Targets
Science (eSpCas9)	2	EMX1(1), VEGFA(1)
Nature (Cas9-HF)	6	EMX1(1), EMX1(2), FANCF1, FANCF3, RUNX1, ZSCAN2
Nature (HypaCas9)	6	FANCF2, FANCF6, DNMT1(3), DNMT1(4), VEGFA(2), VEGFA(3)
NBT(evoCas9)	8	VEGFA2, VEGFA3, EMX1, HEK4, FANCF2, CCR5, CXCR4, PD1

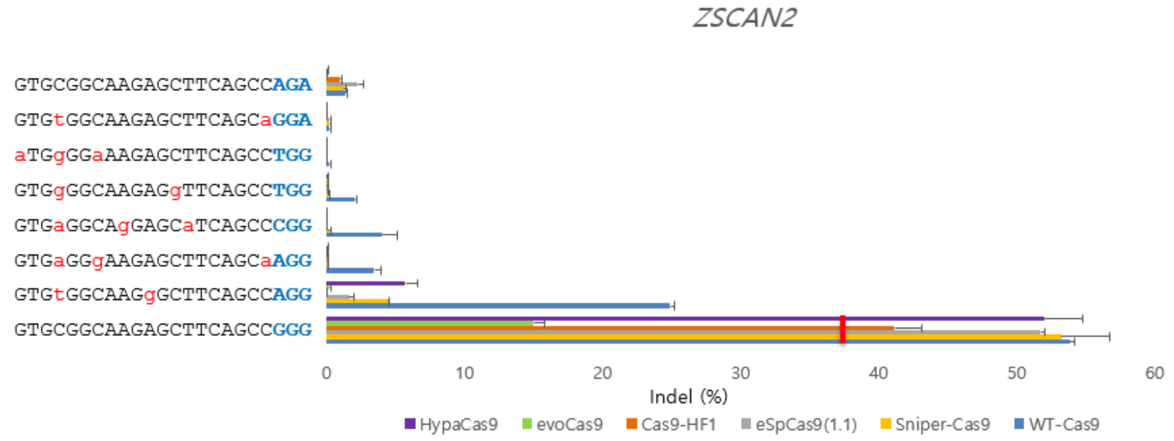

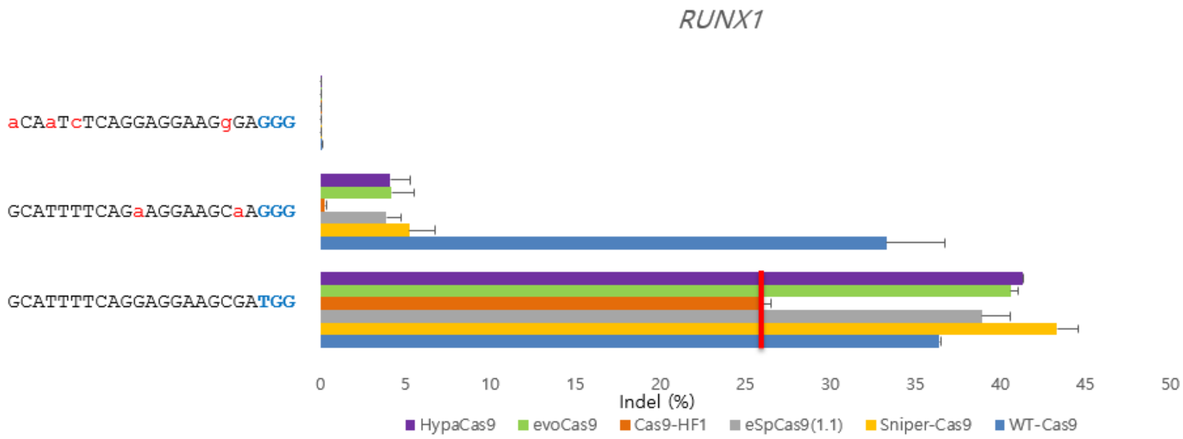

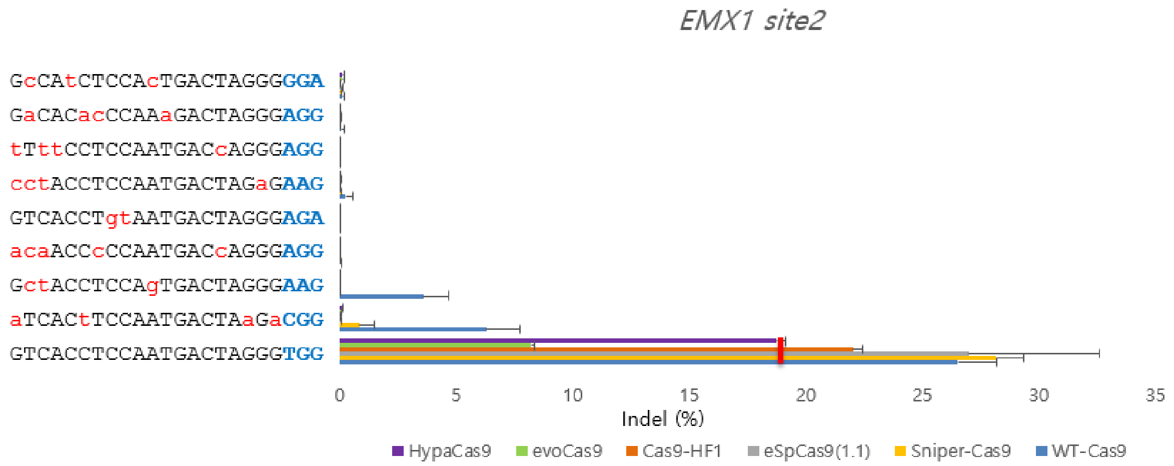

EMX1 site2
GcCAtCTCCAcTGACTAGGGGGA
GaCACacCCAAaGACTAGGGAGG
tTttCCTCCAATGACcAGGGAGG
cctACCTCCAATGACTAGaGAAG
GTCACCTgtAATGACTAGGGAGA
acaACCcCCAATGACcAGGGAGG
GctACCTCCAgTGACTAGGGAAG
aTCACtTCCAATGACTAaGaCGG
GTCACCTCCAATGACTAGGGTGG
0 5 10 15 20 25 30 35
Indel (%)
HypaCas9 evoCas9 Cas9-HF1 eSpCas9(1.1) Sniper-Cas9 WT-Cas9

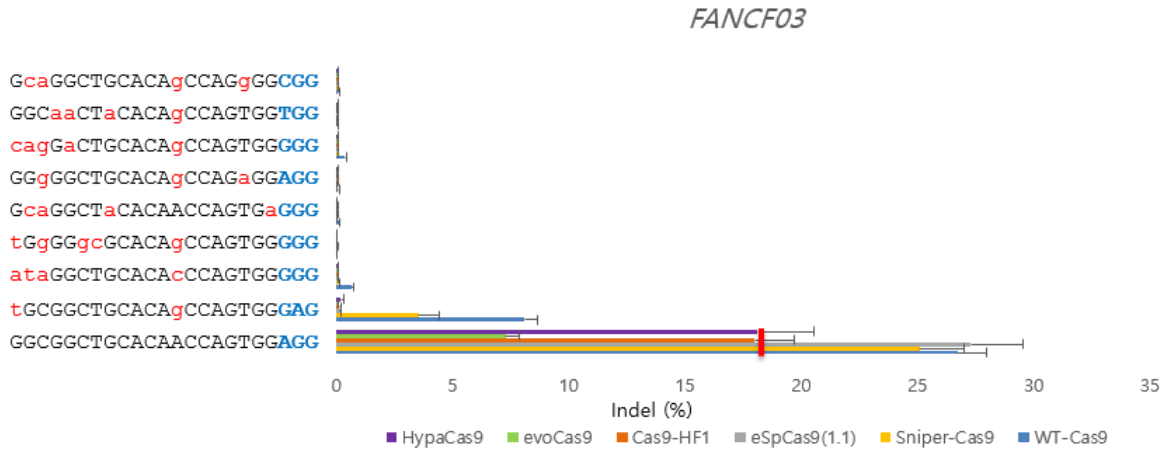

FANCF03
GcaGGCTGCACAgCCAGgGGCGG
GGCaaCTaCACAgCCAGTGGTGG
cagGaCTGCACAgCCAGTGGGGG
GGgGGCTGCACAgCCAGaGGAGG
GcaGGCTaCACAACCAGTGaGGG
tGgGGgcGCACAgCCAGTGGGGG
ataGGCTGCACAcCCAGTGGGGG
tGCGGCTGCACAgCCAGTGGGAG
GGCGGCTGCACAACCAGTGGAGG
0 5 10 15 20 25 30 35
Indel (%)
HypaCas9 evoCas9 Cas9-HF1 eSpCas9(1.1) Sniper-Cas9 WT-Cas9

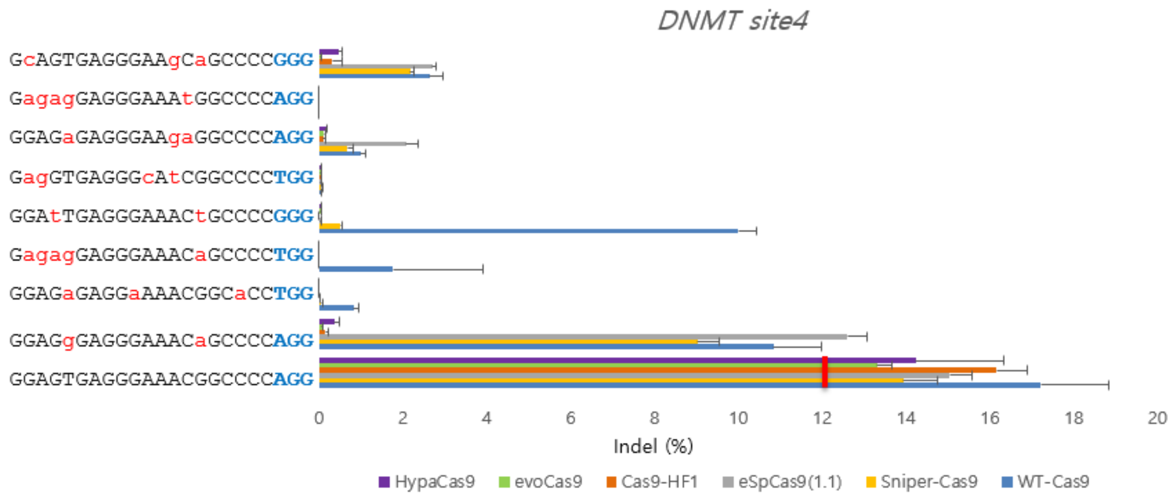

DNMT site4
GcAGTGAGGGAAgCaGCCCCGGG
GagagGAGGGAAAtGGCCCCAGG
GGAGaGAGGGAAgaGGCCCCAGG
GagGTGAGGGcAtCGGCCCCTGG
GGAtTGAGGGAAACtGCCCCGGG
GagagGAGGGAAACaGCCCCTGG
GGAGaGAGGaAAACGGCaCCTGG
GGAGgGAGGGAAACaGCCCCAGG
GGAGTGAGGGAAACGGCCCCAGG
0 2 4 6 8 10 12 14 16 18 20
Indel (%)
HypaCas9 evoCas9 Cas9-HF1 eSpCas9(1.1) Sniper-Cas9 WT-Cas9

Supplementary Figure 17.

Base editor (BE3) on-target activities measured in HEK293t cells. Substitutions were measured using targeted deep sequencing. Substitution of C (represented by green type) to T was measured. The PAM is shown in blue type. Error bars indicate s.e.m. (*n*=3)

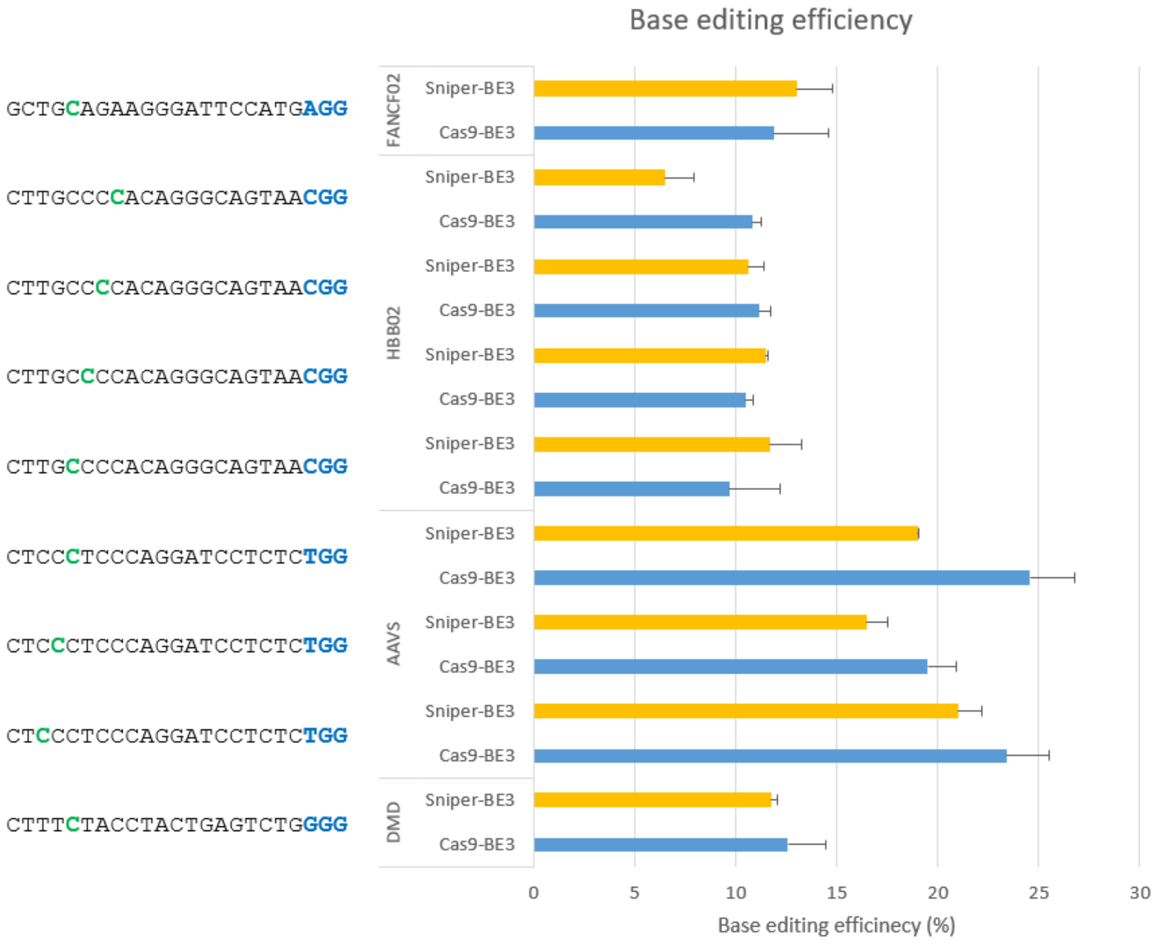

Supplementary Figure 18.

WT-Cas9 and Sniper-Cas9 off-target sites for *AAVS* validated in human primary iPS and T-cells by targeted deep sequencing. Error bars indicate s.e.m. (*n*=3).

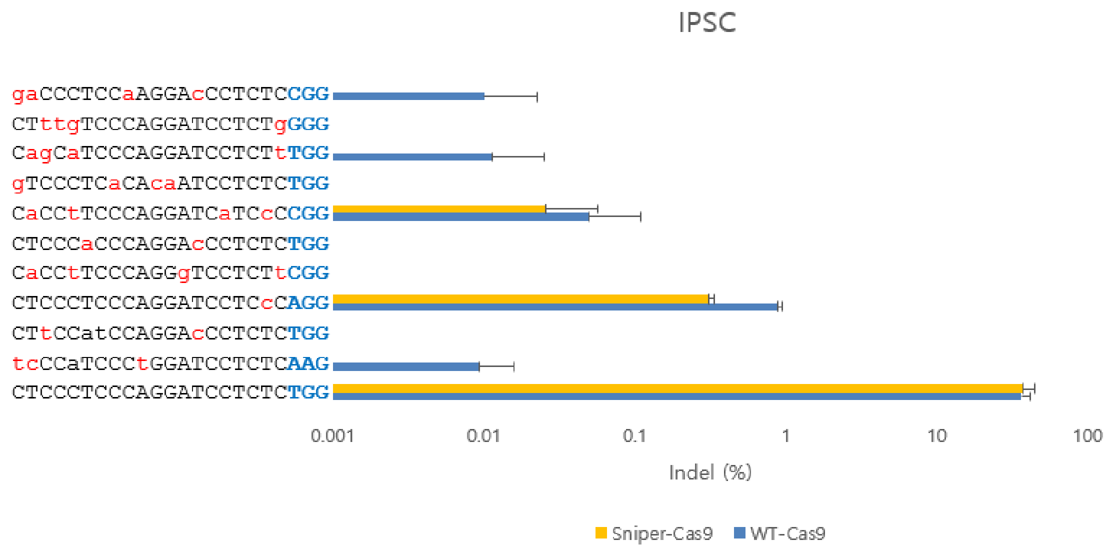

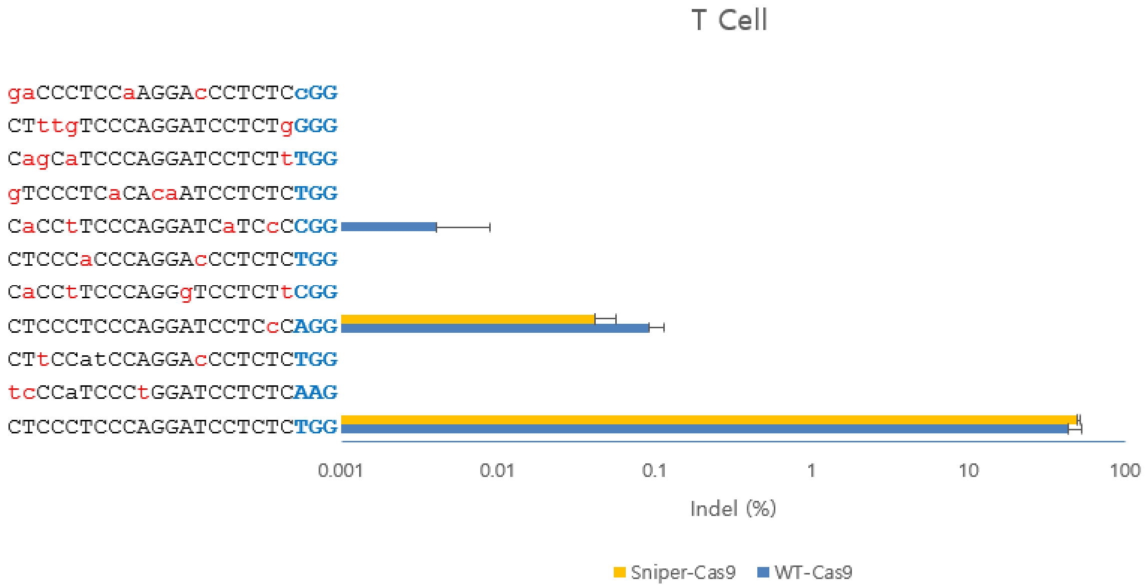

Supplementary Figure 19.

PBLC Sp-Cas9 WT vector Map

CMV Promoter PltetO1 promoter WT-Sp-Cas9 BGH polyA signal p15a origin cat promoter Chloramphenicol Resistance

gaattcagccagcaagacagcgatcccgggcgctgcttcgcgatgtacgggccagatatacgcgttgacattgattattgactagttattaat
agtaatcaattacggggtcattagttcatagcccatatatggagttccgcgttacataacttacggtaaatggcccgcctggctgaccgccca
acgacccccgcccattgacgtcaataatgacgtatgttcccatagtaacgccaatagggactttccattgacgtcaatgggtggagtatttac
ggtaaactgcccacttggcagtacatcaagtgtatcatatgccaagtacgccccctattgacgtcaatgacggtaaatggcccgcctggcatt
atgcccagtacatgaccttatgggactttcctacttggcagtacatctacgtattagtcatcgctattaccatggtgatgcggttttggcagtaca
tcaatgggcgtggatagcggtttgactcacggggatttccaagtctccaccccattgacgtcaatgggagtttgttttggcaccaaaatcaac
gggactttccaaaatgtcgtaacaactccgccccattgacgcaaatgggcggtaggcgtgtacggtgggaggtctatataagcagagctct
ctggctaactagagaacccactgcttactggcttatcgaaatttccctatcagtgatagagattgacatccctatcagtgatagagatactgag
cacatcagcaggacgcactgaccagggagacccaagcttgccaccatggtgtaccccctacgacgtgcccgactacgccgaattgcctccaa
aaaagaagagaaaggtagggatccgaaccatggacaagaagtacagcatcggcctggacatcggtaccaacagcgtgggctgggccg
tgatcaccgacgagtacaaggtgcccagcaagaagttcaaggtgctgggcaacaccgaccgccacagcatcaagaagaacctgatcggc
gccctgctgttcgacagcggcgagaccgccgaggccacccgcctgaagcgcaccgcccgccgccgctacacccgccgcaagaaccgcat
ctgctacctgcaggagatcttcagcaacgagatggccaaggtggacgacagcttcttccaccgcctggaggagagcttcctggtggagga
ggacaagaagcacgagcgccaccccatcttcggcaacatcgtggacgaggtggcctaccacgagaagtaccccaccatctaccacctgcg
caagaagctggtggacagcaccgacaaggccgacctgcgcctgatctacctggccctggcccacatgatcaagttccgcggccacttcctg
atcgagggcgacctgaaccccgacaacagcgacgtggacaagctgttcatccagctggtgcagacctacaaccagctgttcgaggagaa
ccccatcaacgccagcggcgtggacgccaaggccatcctgagcgcccgcctgagcaagagccgccgcctggagaacctgatcgcccag
ctgcccggcgagaagaagaacggcctgttcggcaacctgatcgccctgagcctgggcctgacccccaacttcaagagcaacttcgacctg
gccgaggacgccaagctgcagctgagcaaggacacctacgacgacgacctggacaacctgctggcccagatcggcgaccagtacgccg
acctgttcctggccgccaagaacctgagcgacgccatcctgctgagcgacatcctgcgcgtgaacaccgagatcaccaaggccccctga
gcgccagcatgatcaagcgctacgacgagcaccaccaggacctgaccctgctgaaggccctggtgcgccagcagctgcccgagaagtac
aaggagatcttcttcgaccagagcaagaacggctacgccggctacatcgacggcggcgccagccaggaggagttctacaagttcatcaa
gcccatcctggagaagatggacggcaccgaggagctgctggtgaagctgaaccgcgaggacctgctgcgcaagcagcgcaccttcgac
aacggcagcatcccccaccagatccacctgggcgagctgcacgccatcctgcgccgccaggaggacttctaccccttcctgaaggacaacc
gcgagaagatcgagaagatcctgaccttccgcatcccctactacgtgggccccctggcccgcggcaacagccgcttcgcctggatgaccc
gcaagagcgaggagaccatcaccccctggaacttcgaggaggtggtggacaagggcgccagcgcccagagcttcatcgagcgcatga
ccaacttcgacaagaacctgcccaacgagaaggtgctgcccaagcacagcctgctgtacgagtacttcaccgtgtacaacgagctgaccaa
ggtgaagtacgtgaccgagggcatgcgcaagcccgccttcctgagcggcgagcagaagaaggccatcgtggacctgctgttcaagacc
aaccgcaaggtgaccgtgaagcagctgaaggaggactacttcaagaagatcgagtgcttcgacagcgtggagatcagcggcgtggag
gaccgcttcaacgccagcctgggcacctaccacgacctgctgaagatcatcaaggacaaggacttcctggacaacgaggagaacgagga
catcctggaggacatcgtgctgaccctgaccctgttcgaggaccgcgagatgatcgaggagcgcctgaagacctacgcccacctgttcgac

gacaaggtgatgaagcagctgaagcgccgccgctacaccggctggggccgcctgagccgcaagcttatcaacggcatccgcgacaagc
agagcggcaagaccatcctggacttcctgaagagcgacggcttcgccaaccgcaacttcatgcagctgatccacgacgacagcctgacctt
caaggaggacatccagaaggcccaggtgagcggccagggcgacagcctgcacgagcacatcgccaacctggccggcagccccgccat
caagaagggcatcctgcagaccgtgaaggtggtggacgagctggtgaaggtgatgggccgccacaagcccgagaacatcgtgatcga
gatggcccgcgagaaccagaccacccagaagggccagaagaacagccgcgagcgcatgaagcgcatcgaggagggcatcaaggag
ctgggcagccagatcctgaaggagcaccccgtggagaacacccagctgcagaacgagaagctgtacctgtactacctgcagaacggccg
cgacatgtacgtggaccaggagctggacatcaaccgcctgagcgactacgacgtggaccacatcgtgccccagagcttcctgaaggacg
acagcatcgacaacaaggtgctgacccgcagcgacaagaaccgcggcaagagcgacaacgtgcccagcgaggaggtggtgaagaag
atgaagaactactggcgccagctgctgaacgccaagctgatcacccagcgcaagttcgacaacctgaccaaggccgagcgcggcggcct
gagcgagctggacaaggccggcttcatcaagcgccagctggtggagacccgccagatcaccaagcacgtggcccagatcctggacagc
cgcatgaacaccaagtacgacgagaacgacaagctgatccgcgaggtgaaggtgatcaccctgaagagcaagctggtgagcgacttcc
gcaaggacttccagttctacaaggtgcgcgagatcaacaactaccaccacgcccacgacgcctacctgaacgccgtggtgggcaccgccc
tgatcaagaagtaccccaagctggagagcgagttcgtgtacggcgactacaaggtgtacgacgtgcgcaagatgatcgccaagagcga
gcaggagatcggcaaggccaccgccaagtacttcttctacagcaacatcatgaacttcttcaagaccgagatcaccctggccaacggcgag
atccgcaagcgcccccctgatcgagaccaacggcgagaccggcgagatcgtgtgggacaagggccgcgacttcgccaccgtgcgcaag
gtgctgagcatgccccaggtgaacatcgtgaagaagaccgaggtgcagaccggcggcttcagcaaggagagcatcctgcccaagcgca
acagcgacaagctgatcgcccgcaagaaggactgggaccccaagaagtacggcggcttcgacagccccaccgtggcctacagcgtgct
ggtggtggccaaggtggagaagggcaagagcaagaagctgaagagcgtgaaggagctgctgggcatcaccatcatggagcgcagca
gcttcgagaagaaccccatcgacttcctggaggccaagggctacaaggaggtgaagaaggacctgatcatcaagctgcccaagtacagc
ctgttcgagctggagaacggccgcaagcgcatgctggccagcgccggcgagctgcagaagggcaacgagctggccctgcccagcaag
tacgtgaacttcctgtacctggccagccactacgagaagctgaagggcagccccgaggacaacgagcagaagcagctgttcgtggagca
gcacaagcactacctggacgagatcatcgagcagatcagcgagttcagcaagcgcgtgatcctggccgacgccaacctggacaaggtgc
tgagcgcctacaacaagcaccgcgacaagcccatccgcgagcaggccgagaacatcatccacctgttcaccctgaccaacctgggcgccc
ccgccgccttcaagtacttcgacaccaccatcgaccgcaagcgctacaccagcaccaaggaggtgctggacgccaccctgatccaccaga
gcatcaccggtctgtacgagacccgcatcgacctgagccagctgggcggcgacggcggctccggacctccaaagaaaaagagaaaagt
ataccccctacgacgtgcccgactacgcctaataactcgagcatgcatctagagggccctattctatagtgtcacctaaatgctagagctcgctg
atcagcctcgactgtgccttctagttgccagccatctgttgtttgcccctcccccgtgccttccttgaccctggaaggtgccactcccactgtcctt
tcctaataaaatgaggaaattgcatcgcattgtctgagtaggtgtcattctattctggggggtggggtggggcaggacagcaagggggag
gattgggaagacaatagcaggcatgctggggatgcggtgggctctatggcttgctagcatcacctgtaagtcggacgaattcttaataaga
tgatcttcttgagatcgttttggtctgcgcgtaatctcttgctctgaaaacgaaaaaaccgccttgcagggcggtttttcgaaggttctctgagct
accaactctttgaaccgaggtaactggcttggaggagcgcagtcaccaaaacttgtcctttcagtttagccttaaccggcgcatgacttcaag
actaactcctctaaatcaattaccagtggctgctgccagtggtgcttttgcatgtctttccgggttggactcaagacgatagttaccggataag
gcgcagcggtcggactgaacggggggttcgtgcatacagtccagcttggagcgaactgcctacccggaactgagtgtcaggcgtggaat
gagacaaacgcggccataacagcggaatgacaccggtaaaccgaaaggcaggaacaggagagcgcacgagggagccgccagggg
gaaacgcctggtatctttatagtcctgtcgggtttcgccaccactgatttgagcgtcagatttcgtgatgcttgtcaggggggcggagcctat
ggaaaaacggctttgccgcggccctctcacttccctgttaagtatcttcctggcatcttccaggaaatctccgccccgttcgtcagccatttccgc
tcgccgcagtcgaacgaccgagcgtagcgagtcagtgagcgaggaagcggaatatatcctgtatcacatattctgctgacgcaccggtgc

agccttttttctcctgccacatgaagcacttcactgacaccctcatcagtgccaacatagtaagccagtatacactccgctagcgctgaacctgt
aagtcggacacggaagatcacttcgcagaataaataaatcctggtgtccctgttgataccgggaagccctgggccaacttttggcgaaaat
gagacgttgatcggcacgtaagaggttccaactttcaccataatgaaataagatcactaccgggcgtattttttgagttatcgagattttcagg
agctaaggaagctaaaatggagaaaaaaatcactggatataccaccgttgatatatcccaatggcatcgtaaagaacattttgaggcatttca
gtcagttgctcaatgtacctataaccagaccgttcagctggatattacggcctttttaaagaccgtaaagaaaaataagcacaagttttatccg
gcctttattcacattcttgcccgcctgatgaatgctcatccggaatttcgtatggcaatgaaagacggtgagctggtgatatgggatagtgttc
acccttgttacaccgttttccatgagcaaactgaaacgttttcatcgctctggagtgaataccacgacgatttccggcagtttctacacatatattc
gcaagatgtggcgtgttacggtgaaaacctggcctatttccctaaagggtttattgagaatatgtttttcgtctcagccaatccctgggtgagtt
tcaccagttttgatttaaacgtggccaatatggacaacttcttcgcccccgttttcaccatgggcaaatattatacgcaaggcgacaaggtgctg
atgccgctggcgattcaggttcatcatgccgtttgtgatggcttccatgtcggcagaatgcttaatgaattacaacagtactgcgatgagtgg
cagggcggggcgtaatttttttaaggcagttattggtgcccttaaacgcctggttgctacgcctgaataagtgataataagcggatgaatgg
cagaaattc

sgRNA vector Map containing BsaI site (bold)

PltetO1 promoter gRNA scaffold rrnB T1 terminator pSC101 Ori Rep101(Ts) KanR TetRepressor
Amp promoter

gacgaagactcaattgtccctatcagtgatagagattgacatccctatcagtgatagagatactgagcacgt**gagacc**ggatcc**ggtctcc**
gttttagagctagaaatagcaagttaaaataaggctagtccgttatcaacttgaaaaagtggcaccgagtcggtgctttttttataaaacgaaa
ggctcagtcgaaagactgggcctttcgttttattctagattcgcgatgtacgggccagatatacgcgttgacattgattattgactagttgtcttc
ctgcattaatgaatcggccaacgcgcggggagaggcggtttgcgtattgggcgctcttccgcttcctcgctcactgactcgctgcgctcggt
cgttcggctgcggcgagcggtatcagctcactcaaaggcggtaatacggttatccacagaatcaggggataacgcaggaaagaacgaca
gtaagacgggtaagcctgttgatgataccgctgccttactgggtgcattagccagtctgaatgacctgtcacgggataatccgaagtggtca
gactggaaaatcagagggcaggaactgctgaacagcaaaaagtcagatagcaccacatagcagacccgccataaaacgccctgagaag
cccgtgacgggcttttcttgtattatgggtagtttccttgcatgaatccataaaaggcgcctgtagtgccatttacccccattcactgccagagc
cgtgagcgcagcgaactgaatgtcacgaaaaagacagcgactcaggtgcctgatggtcggagacaaaaggaatattcagcgatttgccc
gagcttgcgagggtgctacttaagcctttagggttttaaggtctgttttgtagaggagcaaacagcgtttgcgacatccttttgtaatactgcg
gaactgactaaagtagtgagttatacacagggctgggatctattctttttatctttttttattctttctttattctataaattataaccacttgaatataa
acaaaaaaaacacacaaaggtctagcggaatttacagagggtctagcagaatttacaagttttccagcaaaggtctagcagaatttacagat
acccacaactcaaaggaaaaggactagtaattatcattgactagcccatctcaattggtatagtgattaaaatcacctagaccaattgagatgt
atgtctgaattagttgttttcaaagcaaatgaactagcgattagtcgctatgacttaacggagcatgaaaccaagctaattttatgctgtgtggc
actactcaaccccacgattgaaaaccctacaaggaaagaacggacggtatcgttcacttataaccaatacgttcagatgatgaacatcagta
gggaaaatgcttatggtgtattagctaaagcaaccagagagctgatgacgagaactgtggaaatcaggaatcctttggttaaaggctttga
gattttccagtggacaaactatgccaagttctcaagcgaaaaattagaattagtttttagtgaagagatattgccttatcttttccagttaaaaaa
attcataaaatataatctggaacatgttaagtcttttgaaaacaaatactctatgaggatttatgagtggttattaaaagaactaacacaaaaga
aaactcacaaggcaaatatagagattagccttgatgaatttaagttcatgttaatgcttgaaaataactaccatgagtttaaaaggcttaacca
atgggttttgaaaccaataagtaaagatttaaacacttacagcaatatgaaattggtggttgataagcgaggccgcccgactgatacgttgat
tttccaagttgaactagatagacaaatggatctcgtaaccgaacttgagaacaaccagataaaaatgaatggtgacaaaataccaacaacca
ttacatcagattcctacctacataacggactaagaaaaacactacacgatgctttaactgcaaaaattcagctcaccagttttgaggcaaattt
ttgagtgacatgcaaagtaagtatgatctcaatggttcgttctcatggctcacgcaaaaacaacgaaccacactagagaacatactggctaaa
tacggaaggatctgaggttcttatggctcttgtatctatcagtgaagcatcaagactaacaaacaaaagtagaacaactgttcaccgttacata
tcaaagggaaaactgtccatatgcacagatgaaaacggtgtaaaaaagatagatacatcagagcttttacgagtttttggtgcatttaaagct
gttcaccatgaacagatcgacaatgtaacagatgaacagcatgtaacacctaatagaacaggtgaaaccagtaaaacaaagcaactagaa
catgaaattgaacacctgagacaacttgttacagctcaacagtcacacatagacagcctgaaacaggcgatgctgcttatcgaatcaaagct
gccgacaacacgggagccagtgacgcctcccgtggggaaaaaatcatggcaattctggaagaaatagcgctttcagccgtctgacgctca
gtggaacgaaaactcacgttaagggattttggtcatgagattatcaaaaaggatcttcacctagatccttttaaattaaaaatgaagttttaaat
caatctaaagtatatatgagtaaacttggtctgacagttagaaaaactcatcgagcatcaaatgaaactgcaatttattcatatcaggattatca
ataccatatttttgaaaaagccgtttctgtaatgaaggagaaaactcaccgaggcagttccataggatggcaagatcctggtatcggtctgcg
attccgactcgtccaacatcaatacaacctattaatttcccctcgtcaaaaataaggttatcaagtgagaaatcaccatgagtgacgactgaatc
cggtgagaatggcaaaagtttatgcatttctttccagacttgttcaacaggccagccattacgctcgtcatcaaaatcactcgcatcaaccaaac

cgttattcattcgtgattgcgcctgagcgagacgaaatacgcgatcgctgttaaaaggacaattacaaacaggaatcgaatgcaaccggcg
caggaacactgccagcgcatcaacaatattttcacctgaatcaggatattcttctaatacctggaatgctgttttcccggggatcgcagtggtg
agtaaccatgcatcatcaggagtacggataaaatgcttgatggtcggaagaggcataaattccgtcagccagtttagtctgaccatctcatct
gtaacatcattggcaacgctacctttgccatgtttcagaaacaactctggcgcatcgggcttcccatacaatcgatagattgtcgcacctgattg
cccgacattatcgcgagcccatttatacccatataaatcagcatccatgttggaatttaatcgcggcctagagcaagacgtttcccgttgaatat
ggctcatcattaattcctattaagacccactttcacatttaagttgtttttctaatccgcatatgatcaattcaaggccgaataagaaggctggctc
tgcaccttggtgatcaaataattcgatagcttgtcgtaataatggcggcatactatcagtagtaggtgtttccctttcttctttagcgacttgatgc
tcttgatcttccaatacgcaacctaaagtaaaatgccccactgcgctgagtgcatataatgcattctctagtgaaaaaccttgttggcataaaaa
ggctaattgattttcgagagtttcatactgtttttctgtaggccgtgtacctaaatgtacttttgctccatcgcgatgacttagtaaagcacatcta
aaacttttagcgttattacgtaaaaaatcttgccagctttccccttctaaagggcaaaagtgagtatggtgcctatctaacatctcaatggctaa
ggcgtcgagcaaagcccgcttatttttacatgccaatacaatgtaggctgctctacacctagcttctgggcgagtttacgggttgttaaacctt
cgattccgacctcattaagcagctctaatgcgctgttaatcactttacttttatctaaacgagacatactcttcctttttcaatattattgaagcattta
tcagggttattgtctcatgagcggatacatatttgaatgtatttagaaaaataaacaaataggggttccgcgcacatttccccgaaaagtgcca
cctgacgtc

Supplementary Figure 20. Images and VCD of iPS and T-cells after RNP transfection

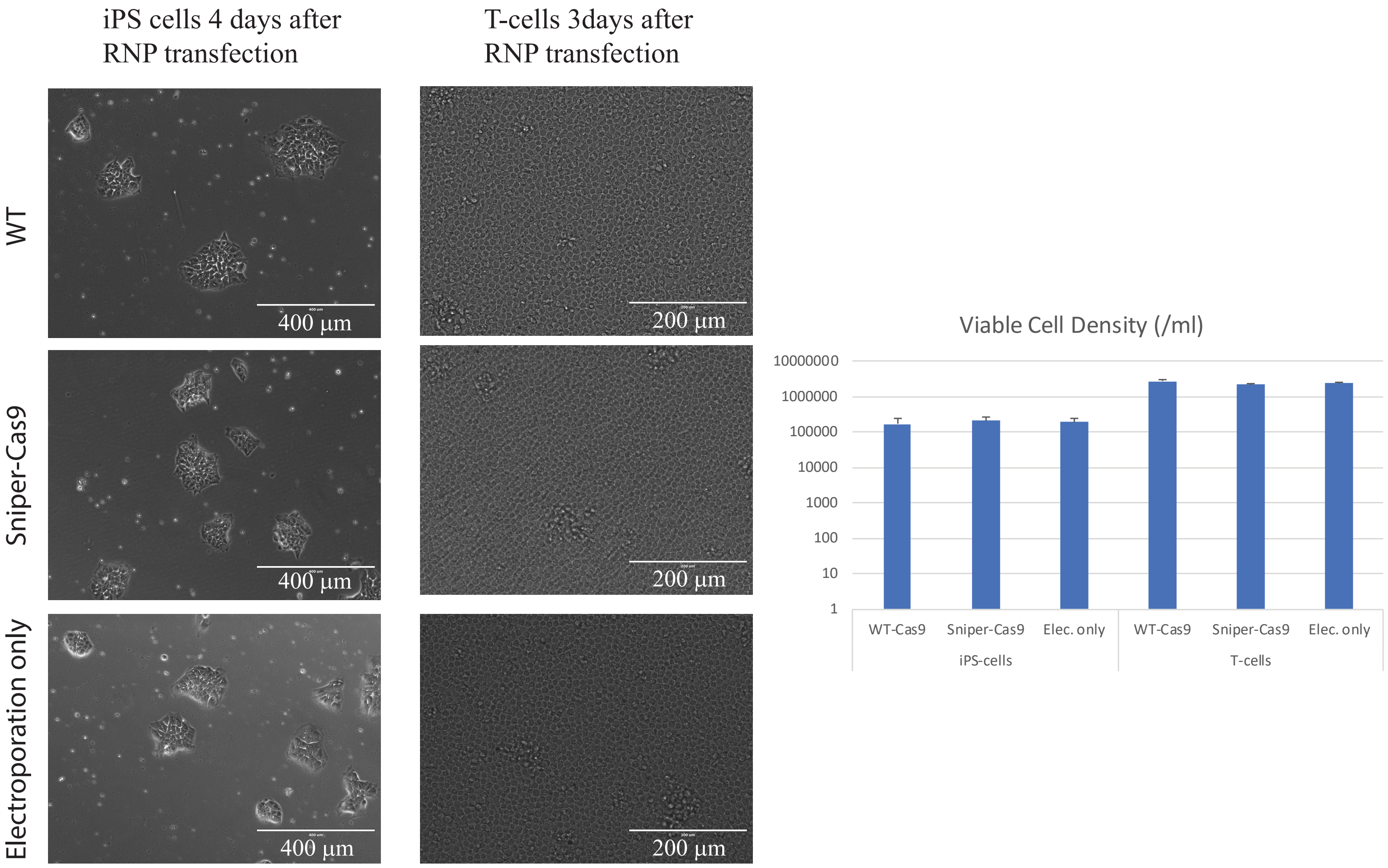

iPS cells transfected with GFP mRNA after 48 hours

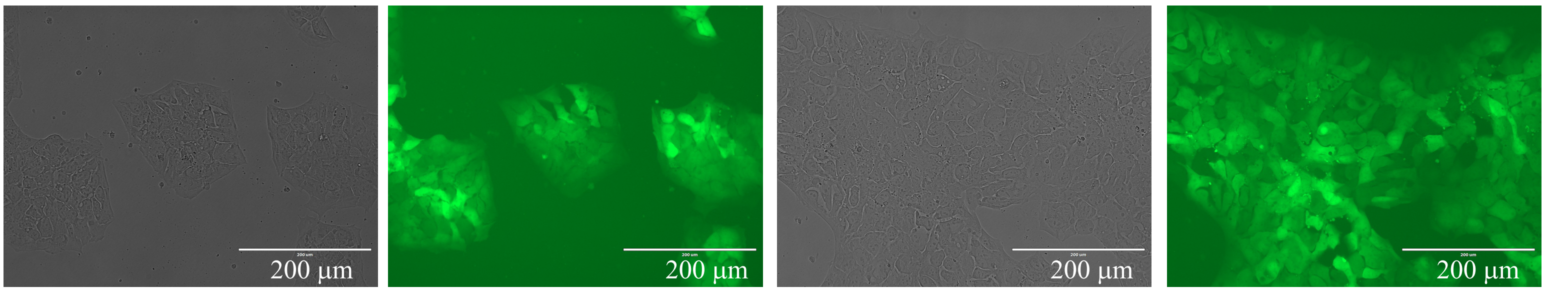

Supplementary Table 1.

Plasmid Construct(Sniper-Screen)

Oligo-cloning p11-lacY-wtx double digested with SphI and XhoI

EMX-56-p11-F	ctagagagtccgagcagaaAGagaaggggcatg
EMX-56-p11-R	cccettctCTttctgctcggactct
EMX-1718-p11-F	ctagagaACccgagcagaagaagaaggggcatg
EMX-1718-p11-R	cccettcttcttctgctcggGTtct
EMX-7-p11-F	ctagagagtccgagcagaGgaagaaggggcatg
EMX-7-p11-R	cccettcttcCtctgctcggactct
EMX-17-p11-F	ctagagaGCccgagcagaagaagaaggggcatg
EMX-17-p11-R	cccettcttcttctgctcggGCtct

Oligo-cloning EMX-1 sgRNA after BsaI digestion

EMX-56-sgRNA-F	gcacggagtccgagcagaaAGagaa
EMX-56-sgRNA-R	AAACttctCTttctgctcggactcC
EMX-1718-sgRNA-F	gcacggaACccgagcagaagaagaa
EMX-1718-sgRNA-R	AAACttcttcttctgctcggGTtcC
EMX-7-sgRNA-F	gcacggagtccgagcagaGgaagaa
EMX-7-sgRNA-R	AAACttcttcCtctgctcggactcC
EMX-17-sgRNA-F	gcacggaGCccgagcagaagaagaa
EMX-17-sgRNA-R	AAACttcttcttctgctcggGCtcC

Supplementary Table 2.

PCR amplification of EMX1 in genomic DNA from HEK 293t cell followed by double digestion using NotI and XhoI to be ligated into pgrg36

EMX1-Not1-F	atgcatgcggccgcccttctgtgaatgttagacccatggg
EMX1-XhoI-R	gactgactcgagcattgcttgtccctctgtcaatgg

PCR amplification of tn7 inserted sequence for sequencing purposes

glms-F	GATGCTGGTGGCGAAGCTGT
glms-R	GATGACGGTTTGTCACATGGA

Supplementary Table 3.

These primers were used to amplify sp-Cas9 followed by Gibson assembly into PBLC-SpCas9 vector double digested with BamHI and XbaI

Cas9-BamHI-30-F	attgcctccaaaaaagaagagaaaggtagg
Cas9-XbaI-30-R	catttaggtgacactatagaatagggccct

Supplementary Table 4.

On/Off Target sequence

On target	Target sequence	Genomic sequence
DMD	CTTTCTACCTACTGAGTCTGGGG	tccCTTTCTACCTACTGAGTCTGGGGtct
AAVS	CTCCCTCCCAGGATCCTCTCTGG	gctCTCCCTCCCAGGATCCTCTCTGGctc
EMX1	GAGTCCGAGCAGAAGAAGAAGGG	cctGAGTCCGAGCAGAAGAAGAAGGGctc
HBB02	CTTGCCCCACAGGGCAGTAACGG	cacCTTGCCCCACAGGGCAGTAACGGcag
HBB03	CACGTTCACCTTGCCCCACAGGG	atcCACGTTCACCTTGCCCCACAGGGcag
HBB04	CCACGTTCACCTTGCCCCACAGG	catCCACGTTCACCTTGCCCCACAGGgca
FANCF01	GGAATCCCTTCTGCAGCACCTGG	gatGGAATCCCTTCTGCAGCACCTGGatg
FANCF02	GCTGCAGAAGGGATTCCATGAGG	ggtGCTGCAGAAGGGATTCCATGAGGtgc
RUNX1	GCATTTTCAGGAGGAAGCGATGG	ggtGCATTTTCAGGAGGAAGCGATGGctt
ZSCAN2	GTGCGGCAAGAGCTTCAGCCGGG	gtaGTGCGGCAAGAGCTTCAGCCGGGgct
HPRT	TCGAGATGTGATGAAGGAGATGG	tgcTCGAGATGTGATGAAGGAGATGGgag
HEK4	GGCACTGCGGCTGGAGGTGG	ggtGGCACTGCGGCTGGAGGTGGggg
VEGFA	GGTGAGTGAGTGTGTGCGTGTGG	gtgGGTGAGTGAGTGTGTGCGTGTGGggt

Off Target	Target sequence	Genomic sequence
DMD	CTTTCTACCTACcGAGTCTGGGG	tcaCTTTCTACCTACcGAGTCTGGGGtct
AAVS	CTCCCTCCCAGGATCCTCCCAGG	tctCTCCCTCCCAGGATCCTCCCAGGaat
EMX1	GAGTTAGAGCAGAAGAAGAAGGG	tcaGAGTTAGAGCAGAAGAAGAAGGGatg
HBB02	tcaGCCCCACAGGGCAGTAAGGG	tgctcaGCCCCACAGGGCAGTAAGGGcag
HBB03	CACGTTCACTTTGCCCCACAGGG	atcCACGTTCACTTTGCCCCACAGGGcat
HBB04	CCACATTCACCTTGCCCCACAGG	cttCCACATTCACCTTGCCCCACAGGctt
FANCF01	GGAAcCCCgTCTGCAGCACCAGG	ccgGGAAcCCCgTCTGCAGCACCAGGccc
FANCF02	GCTGCAGAAGGGATTCCAaGGGG	tatGCTGCAGAAGGGATTCCAaGGGGaat
RUNX1	GCATTTTCAGaAGGAAGCaAGGG	gaaGCATTTTCAGaAGGAAGCaAGGGttc
ZSCAN2	GTGtGGCAAGgGCTTCAGCCAGG	ggaGTGtGGCAAGgGCTTCAGCCAGGctc
HPRT	caGAGATGTGATGtAGGAGAAGG	tttcaGAGATGTGATGtAGGAGAAGGtgg
HEK4	tGCACTGCGGCcGGAGGAGG	tggtGCACTGCGGCcGGAGGAGGtgg

Digenome-seq validation target sequence

	Target sequence
AAVS off target 1	tcCCaTCCCtGGATCCTCTCaaG
AAVS off target 2	CTtCCatCCAGGAcCCTCTCTGG
AAVS off target 3	CTCCCTCCCAGGATCCTCcCaGG
AAVS off target 4	CaCCtTCCCAGGgTCCTCTtcGG
AAVS off target 5	CTCCCaCCCAGGAcCCTCTCTGG
AAVS off target 6	CaCCtTCCCAGGATCaTCcCcGG
AAVS off target 7	gTCCCTCaCAcaATCCTCTCTGG
AAVS off target 8	CagCaTCCCAGGATCCTCTtTGG
AAVS off target 9	CTttgTCCCAGGATCCTCTggGG
AAVS off target 10	gaCCCTCCaAGGAcCCTCTCcGG
AAVS off target 11	gTCCCTCCCAGGAcCCTCTgTGG

	Target sequence
DMD off target 1	CTcTCTACCaACTGAGTCTGGGG
DMD off target 2	CTTTCTACCTACcGAGTCTGGGG
DMD off target 3	CcTTCTACCaACTGAGTCTGGGG
DMD off target 4	tcTTCTACCaACTGAGTCTGGGG
DMD off target 5	CTgTCTACCcACTGAGTCTGGGG
DMD off target 6	tTTTCTACCaACTGAGTCTGGGG
DMD off target 7	accTCTACCaACTGAGTCTGGGG
DMD off target 8	CTcTCTAtCTACTGAGTCTGGGG
DMD off target 9	CTcTCTACCTACTGAGTCTGcaG

	Target sequence
FANCF01 off target 1	GGAgcCCCTTCTGCAG-ACCcGG
FANCF01 off target 2	GGAgTCCCTcCTaCAGCACCaGG
FANCF01 off target 3	tGAATCCCaTCTcCAGCACCaGG
FANCF01 off target 4	aGggTCCCTTCTGCAGCcCCTGG
FANCF01 off target 5	GGAAcaCCTTCTGCAGCtCCaGG
FANCF01 off target 6	GGgAgtCCaTCTGCAGCACCaGG
FANCF01 off target 7	GGAAcCCCgTCTGCAGCACCaGG
FANCF01 off target 8	aGctcCCaTTCTGCAGCACCcGG
FANCF01 off target 9	aGttcCCCaTCTGCAGCACCaGG
FANCF01 off target 10	aGAggCCCcTCTGCAGCACCaGG

	Target sequence
HBB04 off target 1	gCACGcTCACCcTG-CCCACgGG
HBB04 off target 2	CCACGTTCACtTTGCCCCACAGG
HBB04 off target 3	CCctGcTCACCcTGCCCCACtGG
HBB04 off target 4	gCAgGTTCcaCcTGCCCCACtGG
HBB04 off target 5	CagaaTTCACCcTGCCCCACgGG
HBB04 off target 6	gCtCcTcCACCTTGCCCCtCgGG
HBB04 off target 7	gaAtGcTCACCTTGCCCCACAGG
HBB04 off target 8	CCcaGcTCACCcTGCCCCACtGG
HBB04 off target 9	CtgaGgTCACCTTGCCCCACtGG
HBB04 off target 10	CCcCaaaCACCTTGCCCCACtGG

BE3 EMX1 target sequence

	Target seqeunce
EMX1 off target 1	GAGTCtaAGCAGAAGAAGAAGAG
EMX1 off target 2	GAaTCCaAGCAGAAGAAGAgAAG
EMX1 off target 3	aAGTCtGAGCAcAAGAAGAATGG
EMX1 off target 4	GAGTCCGAGCAGAAGAAGAAGGG
EMX1 off target 5	agaatCcAagAGAAGAAGAATGG
EMX1 off target 6	GAGTCCtAGCAGgAGAAGAAGAG

Supplementary Table 5.

On target amplicon primer

On target	Target amplicon primer
DMD_F	Gcagtctccagccagctctt
DMD_R	Atccctgtttgctcgctctc
AAVS_F	Gatcagtgaaacgcaccaga
AAVS_R	cacctcctgttaggcagatt
HBB_F	ctccacatgcccagtttcta
HBB_R	cagggcagagccatctattg
FANCF_F	cccaggtgctgacgtaggta
FANCF_R	ggcttttaagttgcccagag
RUNX_F	tacaggcaaagctgagcaaa
RUNX_R	ccagaggtatccagcagagg
EMX1_F	aggtgaaggtgtggttccag
EMX1_R	agtggccagagtccagctt
ZSCAN2_F	aacctcctcaagcaccagag
ZSCAN2_R	agttccagctaaagcctttcc
HEK4_F	gacccgctggtcttctttc
HEK4_R	aacggagacacacacacagg
HPRT_F	ttgtgtgggtcacaatgctt
HPRT_R	ttgccagccaggtttacaat
VEGFA_F	tgcagacggcagtcactag
VEGFA_R	gctaggaatattgaaggggg

Off target amplicon primer

DMD_F	ggcatgctctcctctgattg
DMD_R	gagatgggatgggaagttca
AAVS_F	gggagaaggccatgaatacag
AAVS_R	cccagtagcactgtggatag
HBB02_F	aaggggaagatcccagagaa
HBB02_R	tgacccactgcatcagaatca
HBB03_F	cccaagagtcttctctgtctac
HBB03_R	aagaaagtgtaagcaacagtcg
HBB04_F	gtcagaaggtgccacaaatc
HBB04_R	tggaacgtctgaggttatcaa
FANCF01_F	acgccagcactttctaagga
FANCF01_R	tgatgccactggagttgttt
FANCF02_F	cccatttctgtctccacctc
FANCF02_R	cccatctttccctcactctg
RUNX_F	gcatgatactttgggggaga
RUNX_R	tctgatcagcaatgttgagatg
EMX1_F	gacaccttttaagatctgacagagaa
EMX1_R	tgcacatgtatgtacaggagtcat
ZSCAN2_F	tctctcctgtgtggattctacagt
ZSCAN2_R	ccgtatcagtgtgatgcatgt
HEK4_F	acctgcacctgtgaaaccac
HEK4_R	cctcctcggagtcctcaagt
HPRT_F	ttgtgtgggtcacaatgctt
HPRT_R	ttgccagccaggtttacaat

Digenome validation target primer

Oligo Name	Primer sequence
AAVS off target 1_F	acaggtgcaatgctcacaag
AAVS off target 1_R	gggtaaactgagggcacaaa
AAVS off target 2_F	tgtgtaaaacccagccatga
AAVS off target 2_R	aggttccacaaggtatggaca
AAVS off target 3_F	tgaattatcgatggtgggaga
AAVS off target 3_R	acaggctctagctgcaggac
AAVS off target 4_F	cttttcccaaactccgatca
AAVS off target 4_R	cagtatccccagccctacag
AAVS off target 5_F	ccccagcactcactttctct
AAVS off target 5_R	tgagattgtcagcctctcca
AAVS off target 6_F	atgactgttcctgggcagat
AAVS off target 6_R	tcacaggatgtgagggagtg
AAVS off target 7_F	tctccatggacacccttcc
AAVS off target 7_R	cgtgtgcatctctgtgtgtg
AAVS off target 8_F	ttcactgtgtccaggagcaa
AAVS off target 8_R	acaaagagaaatggcgaacg
AAVS off target 9_F	gcagttgtgttagaccttgtcc
AAVS off target 9_R	caattgcttaatccctccaaa
AAVS off target 10_F	tctgtcctgttggaggatga
AAVS off target 10_R	ggatcagcattgagccctaa
AAVS off target 11_F	aagcctgggtggaccttc
AAVS off target 11_R	cggtgcccagtaaagaacat

Oligo Name	Primer sequence
DMD off target 1_F	gaggtcatgctgacgactga
DMD off target 1_R	cccaatggattctttcaacc
DMD off target 2_F	gagatgggatgggaagttca
DMD off target 2_R	tctccacccttcaccaattt
DMD off target 3_F	ggatggtgagggtggagaat
DMD off target 3_R	caatcgaatgctgccttttc
DMD off target 4_F	ttcaatgttgccttttccaa
DMD off target 4_R	ctcagtggagagggaagctg
DMD off target 5_F	ggatggtgagggcagataat
DMD off target 5_R	tcctgtttgcccactctttc
DMD off target 6_F	tcctgctctctcctgattgg
DMD off target 6_R	atgggaagggcaggtagtct
DMD off target 7_F	ttcaccgtcacctatttcaca
DMD off target 7_R	cttccccaaagtcaggtcag
DMD off target 8_F	gcagattgtcttccccaaag
DMD off target 8_R	tttgtttgcctgctctctcc
DMD off target 9_F	tgctctttcccaattggttc
DMD off target 9_R	taagcgatggaaatggctct

Oligo Name	Primer sequence
FANCF01 off target 1_F	cgaactcctggggcttct
FANCF01 off target 1_R	gtggggagggagagcagt
FANCF01 off target 2_F	gtgacccaggtccagtgttt
FANCF01 off target 2_R	agccctggagaccttgagtt
FANCF01 off target 3_F	ggtgctggtttaggggagat
FANCF01 off target 3_R	tgtctgattgagtccccaca
FANCF01 off target 4_F	tgggaacatgcaatatgtgt
FANCF01 off target 4_R	tttcaaccacttgaggcaaa
FANCF01 off target 5_F	gagatcggccgagcagac
FANCF01 off target 5_R	agcgctaccacgacatcaag
FANCF01 off target 6_F	agagactgcagggctcagg
FANCF01 off target 6_R	catcagtcatccccttgtca
FANCF01 off target 7_F	acgccagcactttctaagga
FANCF01 off target 7_R	tgatgccactggagttgttt
FANCF01 off target 8_F	gattaggagacgggtgtgga
FANCF01 off target 8_R	gaggaaaactaggcgtgtgc
FANCF01 off target 9_F	cagaaagccagtgggtgtg
FANCF01 off target 9_R	gaggtggggtggagacct
FANCF01 off target 10_F	ggctctgggtacagttctgc
FANCF01 off target 10_R	gggtaacgcgttcactctga

Oligo Name	Primer sequence
HBB04 off target 1_F	ggcctcaccttgaccacat
HBB04 off target 1_R	gcataaacagcacggaagc
HBB04 off target 2_F	acacatgcccagtttccatt
HBB04 off target 2_R	ggaggacaggaccagcataa
HBB04 off target 3_F	aaagcaggctctaaggcaca
HBB04 off target 3_R	acaggcaggacgttgactct
HBB04 off target 4_F	accagcctcacctgaacagt
HBB04 off target 4_R	atgcctgagggaggatca
HBB04 off target 5_F	atcacggcttccttatcgtg
HBB04 off target 5_R	aagtggtaagggggaggttg
HBB04 off target 6_F	aacctccacctctgcatcc
HBB04 off target 6_R	tctgcaattgagaggtcctg
HBB04 off target 7_F	cctaagcacccactttccaa
HBB04 off target 7_R	tggaagctacgagccagtct
HBB04 off target 8_F	tctctttacggaggaactcagc
HBB04 off target 8_R	ccaaacatgcacaaaagcat
HBB04 off target 9_F	gggactgatgcagcctaaat
HBB04 off target 9_R	tccaagaagaggccacagat
HBB04 off target 10_F	cacacatgtggagtcggaac
HBB04 off target 10_R	gacccaacctttttgggaat

BE3 EMX1 amplicon primer

Oligo Name	Primer sequence
EMX1 off target 1_F	atctcacctgggcgagaa
EMX1 off target 1_R	gcctcattatcatcagtgtt
EMX1 off target 2_F	gtcccagaccttcatctcc
EMX1 off target 2_R	cactgtctgcagggctctc
EMX1 off target 3_F	ttggtcccacaggtgaata
EMX1 off target 3_R	tttttggtcaatatctgaaaggtt
EMX1 off target 4_F	gggcctcctgagtttctca
EMX1 off target 4_R	cagcagcaagcagcactc
EMX1 off target 5_F	ctgaaaatttatgacaatttacta
EMX1 off target 5_R	caaacaaagaaggaaagtcc
EMX1 off target 6_F	gcttgcctgtgtgactt
EMX1 off target 6_R	gcccagctgtgcattctat

Supplementary Table 6.

Deep sequencing index primer

Forward primer adapter sequence

D501	tatagcct	AATGATACGGCGACCACCGAGATCTACACtatagcctACACTCTTTCCCTACACGAC
D502	atagaggc	AATGATACGGCGACCACCGAGATCTACACatagaggcACACTCTTTCCCTACACGAC
D503	cctatcct	AATGATACGGCGACCACCGAGATCTACACcctatcctACACTCTTTCCCTACACGAC
D504	ggctctga	AATGATACGGCGACCACCGAGATCTACACggctctgaACACTCTTTCCCTACACGAC
D505	aggcgaag	AATGATACGGCGACCACCGAGATCTACACaggcgaagACACTCTTTCCCTACACGAC
D506	taatctta	AATGATACGGCGACCACCGAGATCTACACtaatcttaACACTCTTTCCCTACACGAC
D507	caggacgt	AATGATACGGCGACCACCGAGATCTACACcaggacgtACACTCTTTCCCTACACGAC
D508	gtactgac	AATGATACGGCGACCACCGAGATCTACACgtactgacACACTCTTTCCCTACACGAC

Reverse primer

D701	cgagtaat	CAAGCAGAAGACGGCATACGAGATcgagtaatGTGACTGGAGTTCAGACGTGT
D702	tctccgga	CAAGCAGAAGACGGCATACGAGATtctccggaGTGACTGGAGTTCAGACGTGT
D703	aatgagcg	CAAGCAGAAGACGGCATACGAGATaatgagcgGTGACTGGAGTTCAGACGTGT
D704	ggaatctc	CAAGCAGAAGACGGCATACGAGATggaatctcGTGACTGGAGTTCAGACGTGT
D705	ttctgaat	CAAGCAGAAGACGGCATACGAGATttctgaatGTGACTGGAGTTCAGACGTGT
D706	acgaattc	CAAGCAGAAGACGGCATACGAGATacgaattcGTGACTGGAGTTCAGACGTGT
D707	agcttcag	CAAGCAGAAGACGGCATACGAGATagcttcagGTGACTGGAGTTCAGACGTGT
D708	gcgcatta	CAAGCAGAAGACGGCATACGAGATgcgcattaGTGACTGGAGTTCAGACGTGT
D709	catagccg	CAAGCAGAAGACGGCATACGAGATcatagccgGTGACTGGAGTTCAGACGTGT
D710	ttcgcgga	CAAGCAGAAGACGGCATACGAGATttcgcggaGTGACTGGAGTTCAGACGTGT
D711	gcgcgaga	CAAGCAGAAGACGGCATACGAGATgcgcgagaGTGACTGGAGTTCAGACGTGT
D712	ctatcgct	CAAGCAGAAGACGGCATACGAGATctatcgctGTGACTGGAGTTCAGACGTGT
