# Supplementary material for: Directed evolution of CRISPR-Cas9 to increase its specificity
Source: Nat Commun. 2018 Aug 6;9:3048. doi: 10.1038/s41467-018-05477-x (PMC6078992; doi:10.1038/s41467-018-05477-x)
Supplement: Supplementary file 3 — Description of Additional Supplementary Files [file 41467_2018_5477_MOESM3_ESM.pdf]

Description of Additional Supplementary Files

File Name: Supplementary Data 1
Description: Protein and DNA sequence alignment of Cas9 variants used in this study
