# Supplementary material for: Directed evolution of CRISPR-Cas9 to increase its specificity
Source: Nat Commun. 2018 Aug 6;9:3048. doi: 10.1038/s41467-018-05477-x (PMC6078992; doi:10.1038/s41467-018-05477-x)
Supplement: Supplementary file 4 — Supplementary Data 1 [file 41467_2018_5477_MOESM4_ESM.pdf]

Supplementary Data 1. Protein sequence alignment of Cas9 variants used in this study

```
Clone-1       1  MDKKYSIGLDIGTNSVGWAVITDEYKVPSKKFKVLGNTDRHSIKKNLIGALLFDSGETAE
Clone-2       1  MDKKYSIGLDIGTNSVGWAVITDEYKVPSKKFKVLGNTDRHSIKKNLIGALLFDSGETAE
Clone-3       1  MDKKYSIGLDIGTNSVGWAVITDEYKVPSKKFKVLGNTDRHSIKKNLIGALLFDSGETAE
WT-Cas9       1  MDKKYSIGLDIGTNSVGWAVITDEYKVPSKKFKVLGNTDRHSIKKNLIGALLFDSGETAE
eSpCas9       1  MDKKYSIGLDIGTNSVGWAVITDEYKVPSKKFKVLGNTDRHSIKKNLIGALLFDSGETAE
Cas9-HF1      1  MDKKYSIGLDIGTNSVGWAVITDEYKVPSKKFKVLGNTDRHSIKKNLIGALLFDSGETAE
evo-Cas9      1  MDKKYSIGLDIGTNSVGWAVITDEYKVPSKKFKVLGNTDRHSIKKNLIGALLFDSGETAE
Hypa-Cas9     1  MDKKYSIGLDIGTNSVGWAVITDEYKVPSKKFKVLGNTDRHSIKKNLIGALLFDSGETAE
xCas9-3.7     1  MDKKYSIGLDIGTNSVGWAVITDEYKVPSKKFKVLGNTDRHSIKKNLIGALLFDSGETAE

Clone-1      61  ATRLKRTARRRYTRRKNRICYLQEIFSNEMAKVDDSFFHRLEESFLVEEDKKHERHPIFG
Clone-2      61  ATRLKRTARRRYTRRKNRICYLQEIFSNEMAKVDDSFFHRLEESFLVEEDKKHERHPIFG
Clone-3      61  ATRLKRTARRRYTRRKNRICYLQEIFSNEMAKVDDSFFHRLEESFLVEEDKKHERHPIFG
WT-Cas9      61  ATRLKRTARRRYTRRKNRICYLQEIFSNEMAKVDDSFFHRLEESFLVEEDKKHERHPIFG
eSpCas9      61  ATRLKRTARRRYTRRKNRICYLQEIFSNEMAKVDDSFFHRLEESFLVEEDKKHERHPIFG
Cas9-HF1     61  ATRLKRTARRRYTRRKNRICYLQEIFSNEMAKVDDSFFHRLEESFLVEEDKKHERHPIFG
evo-Cas9     61  ATRLKRTARRRYTRRKNRICYLQEIFSNEMAKVDDSFFHRLEESFLVEEDKKHERHPIFG
Hypa-Cas9    61  ATRLKRTARRRYTRRKNRICYLQEIFSNEMAKVDDSFFHRLEESFLVEEDKKHERHPIFG
xCas9-3.7    61  ATRLKRTARRRYTRRKNRICYLQEIFSNEMAKVDDSFFHRLEESFLVEEDKKHERHPIFG

Clone-1     121  NIVDEVAYHEKYPTIYHLRKKLVDSTDKADLRLIYLALAHMIKFRGHFLIEGDLNPDNSD
Clone-2     121  NIVDEVAYHEKYPTIYHLRKKLVDSTDKADLRLIYLALAHMIKFRGHFLIEGDLNPDNSD
Clone-3     121  NIVDEVAYHEKYPTIYHLRKKLVDSTDKADLRLIYLALAHMIKFRGHFLIEGDLNPDNSD
WT-Cas9     121  NIVDEVAYHEKYPTIYHLRKKLVDSTDKADLRLIYLALAHMIKFRGHFLIEGDLNPDNSD
eSpCas9     121  NIVDEVAYHEKYPTIYHLRKKLVDSTDKADLRLIYLALAHMIKFRGHFLIEGDLNPDNSD
Cas9-HF1    121  NIVDEVAYHEKYPTIYHLRKKLVDSTDKADLRLIYLALAHMIKFRGHFLIEGDLNPDNSD
evo-Cas9    121  NIVDEVAYHEKYPTIYHLRKKLVDSTDKADLRLIYLALAHMIKFRGHFLIEGDLNPDNSD
Hypa-Cas9   121  NIVDEVAYHEKYPTIYHLRKKLVDSTDKADLRLIYLALAHMIKFRGHFLIEGDLNPDNSD
xCas9-3.7   121  NIVDEVAYHEKYPTIYHLRKKLVDSTDKADLRLIYLALAHMIKFRGHFLIEGDLNPDNSD

Clone-1     181  VDKLFIQLVQTYNQLFEENPINASGVDAKAILSARLSKSRRLENLIAQLPGEKKNGLFGN
Clone-2     181  VDKLFIQLVQTYNQLFEENPINASGVDAKAILSARLSKSRRLENLIAQLPGEKKNGLFGN
Clone-3     181  VDKLFIQLVQTYNQLFEENPINDSGVDAKAILSARLSKSRRLENLIAQLPGEKKNGLFGN
WT-Cas9     181  VDKLFIQLVQTYNQLFEENPINASGVDAKAILSARLSKSRRLENLIAQLPGEKKNGLFGN
eSpCas9     181  VDKLFIQLVQTYNQLFEENPINASGVDAKAILSARLSKSRRLENLIAQLPGEKKNGLFGN
Cas9-HF1    181  VDKLFIQLVQTYNQLFEENPINASGVDAKAILSARLSKSRRLENLIAQLPGEKKNGLFGN
evo-Cas9    181  VDKLFIQLVQTYNQLFEENPINASGVDAKAILSARLSKSRRLENLIAQLPGEKKNGLFGN
Hypa-Cas9   181  VDKLFIQLVQTYNQLFEENPINASGVDAKAILSARLSKSRRLENLIAQLPGEKKNGLFGN
xCas9-3.7   181  VDKLFIQLVQTYNQLFEENPINASGVDAKAILSARLSKSRRLENLIAQLPGEKKNGLFGN

Clone-1     241  LIALSLGLTPNFKSNFDLAEDAKLQLSKDTYDDDLDNLLAQIGDQYADLFLAAKNLSDAI
Clone-2     241  LIALSLGLTPNFKSNFDLAEDAKLQLSKDTYDDDLDNLLAQIGDQYADLFLAAKNLSDAI
Clone-3     241  LIALSLGLTPNFKSNFDLAEDAKLQLSKDTYDDDLDHLLAQIGDQYADLFLAAKNLSDAI
WT-Cas9     241  LIALSLGLTPNFKSNFDLAEDAKLQLSKDTYDDDLDNLLAQIGDQYADLFLAAKNLSDAI
eSpCas9     241  LIALSLGLTPNFKSNFDLAEDAKLQLSKDTYDDDLDNLLAQIGDQYADLFLAAKNLSDAI
Cas9-HF1    241  LIALSLGLTPNFKSNFDLAEDAKLQLSKDTYDDDLDNLLAQIGDQYADLFLAAKNLSDAI
evo-Cas9    241  LIALSLGLTPNFKSNFDLAEDAKLQLSKDTYDDDLDNLLAQIGDQYADLFLAAKNLSDAI
Hypa-Cas9   241  LIALSLGLTPNFKSNFDLAEDAKLQLSKDTYDDDLDNLLAQIGDQYADLFLAAKNLSDAI
xCas9-3.7   241  LIALSLGLTPNFKSNFDLAEDTKLQLSKDTYDDDLDNLLAQIGDQYADLFLAAKNLSDAI

Clone-1     301  LLSDILRVNTEITKAPLSASMIKRYDEHHQDLTLLKALVRQQLPEKYKEIFFDQSKNGYA
Clone-2     301  LLSDILRVNTEITKAPLSASMIKRYDEHHQDLTLLKALVRQQLPEKYKEIFFDQSKNGYA
Clone-3     301  LLSDILRVNTEITKAPLSASMIKRYDEHHQDLTLLKALVRQQLPEKYKEIFFDQSKNGYA
WT-Cas9     301  LLSDILRVNTEITKAPLSASMIKRYDEHHQDLTLLKALVRQQLPEKYKEIFFDQSKNGYA
eSpCas9     301  LLSDILRVNTEITKAPLSASMIKRYDEHHQDLTLLKALVRQQLPEKYKEIFFDQSKNGYA
Cas9-HF1    301  LLSDILRVNTEITKAPLSASMIKRYDEHHQDLTLLKALVRQQLPEKYKEIFFDQSKNGYA
evo-Cas9    301  LLSDILRVNTEITKAPLSASMIKRYDEHHQDLTLLKALVRQQLPEKYKEIFFDQSKNGYA
Hypa-Cas9   301  LLSDILRVNTEITKAPLSASMIKRYDEHHQDLTLLKALVRQQLPEKYKEIFFDQSKNGYA
xCas9-3.7   301  LLSDILRVNTEITKAPLSASMIKLYDEHHQDLTLLKALVRQQLPEKYKEIFFDQSKNGYA
```

```
Clone-1    361 GYIDGGASQEEFYKFIKPILEKMDGTEELLVKLNREDLLRKQRTFDNGSIPHQIHLGELH
Clone-2    361 GYIDGGASQEEFYKFIKPILEKMDGTEELLVKLNREDLLRKQRTFDNGSIPHQIHLGELH
Clone-3    361 GYIDGSASQEEFYKFIKPILEKMDGTEELLVKLNREDLLRKQRTFDNGSIPHQIHLGELH
WT-Cas9    361 GYIDGGASQEEFYKFIKPILEKMDGTEELLVKLNREDLLRKQRTFDNGSIPHQIHLGELH
eSpCas9    361 GYIDGGASQEEFYKFIKPILEKMDGTEELLVKLNREDLLRKQRTFDNGSIPHQIHLGELH
Cas9-HF1   361 GYIDGGASQEEFYKFIKPILEKMDGTEELLVKLNREDLLRKQRTFDNGSIPHQIHLGELH
evo-Cas9   361 GYIDGGASQEEFYKFIKPILEKMDGTEELLVKLNREDLLRKQRTFDNGSIPHQIHLGELH
Hypa-Cas9  361 GYIDGGASQEEFYKFIKPILEKMDGTEELLVKLNREDLLRKQRTFDNGSIPHQIHLGELH
xCas9-3.7  361 GYIDGGASQEEFYKFIKPILEKMDGTEELLVKLNREDLLRKQRTFDNGIIPHQIHLGELH

Clone-1    421 AILRRQEDFYPFLKDNREKIEKILTFRIPYYVGPLARGNSRFAWMTRKSEETITPWNFEE
Clone-2    421 AILRRQEDFYPFLKDNREKIEKILTFRIPYYVGPLARGNSRFAWMTRKSEETITPWNFEE
Clone-3    421 AILRRQEDFYPFLKDNREKIEKILTFRIPYYVGPLARGNSRFAWMTRKSEETITPWNFEE
WT-Cas9    421 AILRRQEDFYPFLKDNREKIEKILTFRIPYYVGPLARGNSRFAWMTRKSEETITPWNFEE
eSpCas9    421 AILRRQEDFYPFLKDNREKIEKILTFRIPYYVGPLARGNSRFAWMTRKSEETITPWNFEE
Cas9-HF1   421 AILRRQEDFYPFLKDNREKIEKILTFRIPYYVGPLARGNSRFAWMTRKSEETITPWNFEE
evo-Cas9   421 AILRRQEDFYPFLKDNREKIEKILTFRIPYYVGPLARGNSRFAWMTRKSEETITPWNFEE
Hypa-Cas9  421 AILRRQEDFYPFLKDNREKIEKILTFRIPYYVGPLARGNSRFAWMTRKSEETITPWNFEE
xCas9-3.7  421 AILRRQEDFYPFLKDNREKIEKILTFRIPYYVGPLARGNSRFAWMTRKSEETITPWNFEK

Clone-1    481 VVDKGASAQSFIERMTNFDKNLPNEKVLPKHSLLYEYFTVYNELTKVKYVTEGMRKPASL
Clone-2    481 VVDKGASAQSFIERMTNFDKNLPNEKVLPKHSLLYEYFTVYNELTKVKYVTEGMRKPAFL
Clone-3    481 VVDKGASAQSFIERMTNFDKNLPNEKVLPKHSLLYEYFTVYNELTKVKYVTEGMRKPAFL
WT-Cas9    481 VVDKGASAQSFIERMTNFDKNLPNEKVLPKHSLLYEYFTVYNELTKVKYVTEGMRKPAFL
eSpCas9    481 VVDKGASAQSFIERMTNFDKNLPNEKVLPKHSLLYEYFTVYNELTKVKYVTEGMRKPAFL
Cas9-HF1   481 VVDKGASAQSFIERMTAFDKNLPNEKVLPKHSLLYEYFTVYNELTKVKYVTEGMRKPAFL
evo-Cas9   481 VVDKGASAQSFIERVTNFDKNLPNEKVLPKHSLLNEYFTVYNELTKEKYVTEGMRKPAFL
Hypa-Cas9  481 VVDKGASAQSFIERMTNFDKNLPNEKVLPKHSLLYEYFTVYNELTKVKYVTEGMRKPAFL
xCas9-3.7  481 VVDKGASAQSFIERMTNFDKNLPNEKVLPKHSLLYEYFTVYNELTKVKYVTEGMRKPAFL

Clone-1    541 SGEQKKAIVDLLFKTNRKVTVKQLKEDYFKKIECFDSVEISGVEDRFNASLGTYHDLLKI
Clone-2    541 SGEQKKAIVDLLFKTNRKVTVKQLKEDYFKKIECFDSVEISGVEDRFNASLGTYHDLLKI
Clone-3    541 SGEQKKAIVDLLFKTNRKVTVKQLKEDYFKKIECFDSVEISGVEDRFNASLGTYHDLLKI
WT-Cas9    541 SGEQKKAIVDLLFKTNRKVTVKQLKEDYFKKIECFDSVEISGVEDRFNASLGTYHDLLKI
eSpCas9    541 SGEQKKAIVDLLFKTNRKVTVKQLKEDYFKKIECFDSVEISGVEDRFNASLGTYHDLLKI
Cas9-HF1   541 SGEQKKAIVDLLFKTNRKVTVKQLKEDYFKKIECFDSVEISGVEDRFNASLGTYHDLLKI
evo-Cas9   541 SGEQKKAIVDLLFKTNRKVTVKQLKEDYFKKIECFDSVEISGVEDRFNASLGTYHDLLKI
Hypa-Cas9  541 SGEQKKAIVDLLFKTNRKVTVKQLKEDYFKKIECFDSVEISGVEDRFNASLGTYHDLLKI
xCas9-3.7  541 SGDQKKAIVDLLFKTNRKVTVKQLKEDYFKKIECFDSVEISGVEDRFNASLGTYHDLLKI

Clone-1    601 IKDKDFLDNEENEDILEDIVLTLTLFEDREMIEERLKTYAHLFDDKVMKQLKRRRYTGWG
Clone-2    601 NKDKDFLDNEENEDILEDIVLTLTLFEDREMIEERLKTYAHLFDDKVMKQLKRRRYTGWG
Clone-3    601 IKDKDFLDNEENEDILEDIVLTLTLFEDREMIEERLKTYAHLFDDKVMKQLKRRRYTGWG
WT-Cas9    601 IKDKDFLDNEENEDILEDIVLTLTLFEDREMIEERLKTYAHLFDDKVMKQLKRRRYTGWG
eSpCas9    601 IKDKDFLDNEENEDILEDIVLTLTLFEDREMIEERLKTYAHLFDDKVMKQLKRRRYTGWG
Cas9-HF1   601 IKDKDFLDNEENEDILEDIVLTLTLFEDREMIEERLKTYAHLFDDKVMKQLKRRRYTGWG
evo-Cas9   601 IKDKDFLDNEENEDILEDIVLTLTLFEDREMIEERLKTYAHLFDDKVMKQLKRRRYTGWG
Hypa-Cas9  601 IKDKDFLDNEENEDILEDIVLTLTLFEDREMIEERLKTYAHLFDDKVMKQLKRRRYTGWG
xCas9-3.7  601 IKDKDFLDNEENEDILEDIVLTLTLFEDREMIEERLKTYAHLFDDKVMKQLKRRRYTGWG

Clone-1    661 RLSRKLINGIRDKQSGKTILDFLKSDGFANRNFMQLIHDDSLTFKEDIQKAQVSGQGDSL
Clone-2    661 RLSRKLINGIRDKQSGKTILDFLKSDGFANRNFMQLIHDDSLTFKEDIQKAQVSGQGDSL
Clone-3    661 RLSRKLINGIRDKQSGKTILDFLKSDGFANRNFMQLIHDDSLTFKEDIQKAQVSGQGDSL
WT-Cas9    661 RLSRKLINGIRDKQSGKTILDFLKSDGFANRNFMQLIHDDSLTFKEDIQKAQVSGQGDSL
eSpCas9    661 RLSRKLINGIRDKQSGKTILDFLKSDGFANRNFMQLIHDDSLTFKEDIQKAQVSGQGDSL
Cas9-HF1   661 ALSRKLINGIRDKQSGKTILDFLKSDGFANRNFMALIHDDSLTFKEDIQKAQVSGQGDSL
evo-Cas9   661 QLSRKLINGIRDKQSGKTILDFLKSDGFANRNFMQLIHDDSLTFKEDIQKAQVSGQGDSL
Hypa-Cas9  661 RLSRKLINGIRDKQSGKTILDFLKSDGFANRAFAALIADDSLTFKEDIQKAQVSGQGDSL
xCas9-3.7  661 RLSRKLINGIRDKQSGKTILDFLKSDGFANRNFIQLIHDDSLTFKEDIQKAQVSGQGDSL
```

```
Clone-1     721  HEHIANLAGSPAIKKGILQTVKVVDELVKVMGRHKPENIVIEIARENQTTQKGQKNSRER
Clone-2     721  HEHIANLAGSPAIKKGILQTVKVVDELVKVMGRHKPENIVIEMARENQTTQKGQKNSRER
Clone-3     721  HEHIANLAGSPAIKKGILQTVKVVDELVKVMGRHKPENIVIEIARENQTTQKGQKNSRER
WT-Cas9     721  HEHIANLAGSPAIKKGILQTVKVVDELVKVMGRHKPENIVIEMARENQTTQKGQKNSRER
eSpCas9     721  HEHIANLAGSPAIKKGILQTVKVVDELVKVMGRHKPENIVIEMARENQTTQKGQKNSRER
Cas9-HF1    721  HEHIANLAGSPAIKKGILQTVKVVDELVKVMGRHKPENIVIEMARENQTTQKGQKNSRER
evo-Cas9    721  HEHIANLAGSPAIKKGILQTVKVVDELVKVMGRHKPENIVIEMARENQTTQKGQKNSRER
Hypa-Cas9   721  HEHIANLAGSPAIKKGILQTVKVVDELVKVMGRHKPENIVIEMARENQTTQKGQKNSRER
xCas9-3.7   721  HEHIANLAGSPAIKKGILQTVKVVDELVKVMGRHKPENIVIEMARENQTTQKGQKNSRER

Clone-1     781  MKRIEEGIKELGSQILKEHPVENTQLQNEKLYLYYLQNGRDMYVDQELDINRLSDYDVDH
Clone-2     781  MKRIEEGIKELGSQILKEHPVENTQLQNEKLYLYYLQNGRDMYVDQELDINRLSDYDVDH
Clone-3     781  MKRIEEGIKELGSQILKEHPVENTQLQNEKLYLYYLQNGRDMYVDQELDINRLSDYDVDH
WT-Cas9     781  MKRIEEGIKELGSQILKEHPVENTQLQNEKLYLYYLQNGRDMYVDQELDINRLSDYDVDH
eSpCas9     781  MKRIEEGIKELGSQILKEHPVENTQLQNEKLYLYYLQNGRDMYVDQELDINRLSDYDVDH
Cas9-HF1    781  MKRIEEGIKELGSQILKEHPVENTQLQNEKLYLYYLQNGRDMYVDQELDINRLSDYDVDH
evo-Cas9    781  MKRIEEGIKELGSQILKEHPVENTQLQNEKLYLYYLQNGRDMYVDQELDINRLSDYDVDH
Hypa-Cas9   781  MKRIEEGIKELGSQILKEHPVENTQLQNEKLYLYYLQNGRDMYVDQELDINRLSDYDVDH
xCas9-3.7   781  MKRIEEGIKELGSQILKEHPVENTQLQNEKLYLYYLQNGRDMYVDQELDINRLSDYDVDH

Clone-1     841  IVPQSFLKDDSIDNKVLTRSDKNRGKSDNVPSEEVVKKMKNYWRQLLNANLITQRKFDNL
Clone-2     841  IVPQSFLKDDSIDNKVLTRSDKNRGKSDNVPSEEVVKKMKNYWRQLLNAKLITQRKFDNL
Clone-3     841  IVPQSFLKDDSIDNKVLTRSDKNRGKSDNVPSEEVVKKMKNYWRQLLNAKLITQRKFDNL
WT-Cas9     841  IVPQSFLKDDSIDNKVLTRSDKNRGKSDNVPSEEVVKKMKNYWRQLLNAKLITQRKFDNL
eSpCas9     841  IVPQSFLADDSIDNKVLTRSDKNRGKSDNVPSEEVVKKMKNYWRQLLNAKLITQRKFDNL
Cas9-HF1    841  IVPQSFLKDDSIDNKVLTRSDKNRGKSDNVPSEEVVKKMKNYWRQLLNAKLITQRKFDNL
evo-Cas9    841  IVPQSFLKDDSIDNKVLTRSDKNRGKSDNVPSEEVVKKMKNYWRQLLNAKLITQRKFDNL
Hypa-Cas9   841  IVPQSFLKDDSIDNKVLTRSDKNRGKSDNVPSEEVVKKMKNYWRQLLNAKLITQRKFDNL
xCas9-3.7   841  IVPQSFLKDDSIDNKVLTRSDKNRGKSDNVPSEEVVKKMKNYWRQLLNAKLITQRKFDNL

Clone-1     901  TKAERGGLSELDKAGFIKRQLVETRQITKHVAQILDSRMNTKYDENDKLIREVKVITLKS
Clone-2     901  TKAERGGLSELDKAGFIKRQLVETRQITKHVAQILDSRMNTKYDENDKLIREVKVITLKS
Clone-3     901  TKAERGGLSELDKAGFIKRQLVETRQITKHVAQILDSRMNTKYDENDKLIREVKVITLKS
WT-Cas9     901  TKAERGGLSELDKAGFIKRQLVETRQITKHVAQILDSRMNTKYDENDKLIREVKVITLKS
eSpCas9     901  TKAERGGLSELDKAGFIKRQLVETRQITKHVAQILDSRMNTKYDENDKLIREVKVITLKS
Cas9-HF1    901  TKAERGGLSELDKAGFIKRQLVETRAITKHVAQILDSRMNTKYDENDKLIREVKVITLKS
evo-Cas9    901  TKAERGGLSELDKAGFIKRQLVETRQITKHVAQILDSRMNTKYDENDKLIREVKVITLKS
Hypa-Cas9   901  TKAERGGLSELDKAGFIKRQLVETRQITKHVAQILDSRMNTKYDENDKLIREVKVITLKS
xCas9-3.7   901  TKAERGGLSELDKAGFIKRQLVETRQITKHVAQILDSRMNTKYDENDKLIREVKVITLKS

Clone-1     961  KLVSDFRKDFQFYKVREINNYHHAHDAYLNAVVGTALIKKYPKLESEFVYGDYKVYDVRK
Clone-2     961  KLVSYFRKDFQFYKVREINNYHHAHDAYLNAVVGTALIKKYPKLESEFVYGDYKVYDVRK
Clone-3     961  KLVSDFRKDFQFYKVREINNYHHAHDAYLNAVVGTALIKKYPKLESEFVYGDYKVYDVRK
WT-Cas9     961  KLVSDFRKDFQFYKVREINNYHHAHDAYLNAVVGTALIKKYPKLESEFVYGDYKVYDVRK
eSpCas9     961  KLVSDFRKDFQFYKVREINNYHHAHDAYLNAVVGTALIKKYPALESEFVYGDYKVYDVRK
Cas9-HF1    961  KLVSDFRKDFQFYKVREINNYHHAHDAYLNAVVGTALIKKYPKLESEFVYGDYKVYDVRK
evo-Cas9    961  KLVSDFRKDFQFYKVREINNYHHAHDAYLNAVVGTALIKKYPKLESEFVYGDYKVYDVRK
Hypa-Cas9   961  KLVSDFRKDFQFYKVREINNYHHAHDAYLNAVVGTALIKKYPKLESEFVYGDYKVYDVRK
xCas9-3.7   961  KLVSDFRKDFQFYKVREINNYHHAHDAYLNAVVGTALIKKYPKLESEFVYGDYKVYDVRK

Clone-1    1021  MIAKSEQEIGKATAKYFFYSNIMNFFKTEITLANGEIRKRPLIETNGETGEIVWDKGRDF
Clone-2    1021  MIAKSEQEIGKATAKYFFYSNIMNFFKTEITLANGEIRKRPLIETNGETGEIVWDKGRDF
Clone-3    1021  MIAKSEQEIGKATAKYFYYSNIMNFFKTEITLANGEIRKRPLIETNGETGEIVWDKGRDF
WT-Cas9    1021  MIAKSEQEIGKATAKYFFYSNIMNFFKTEITLANGEIRKRPLIETNGETGEIVWDKGRDF
eSpCas9    1021  MIAKSEQEIGKATAKYFFYSNIMNFFKTEITLANGEIRKAPLIETNGETGEIVWDKGRDF
Cas9-HF1   1021  MIAKSEQEIGKATAKYFFYSNIMNFFKTEITLANGEIRKRPLIETNGETGEIVWDKGRDF
evo-Cas9   1021  MIAKSEQEIGKATAKYFFYSNIMNFFKTEITLANGEIRKRPLIETNGETGEIVWDKGRDF
Hypa-Cas9  1021  MIAKSEQEIGKATAKYFFYSNIMNFFKTEITLANGEIRKRPLIETNGETGEIVWDKGRDF
xCas9-3.7  1021  MIAKSEQEIGKATAKYFFYSNIMNFFKTEITLANGEIRKRPLIETNGETGEIVWDKGRDF
```

```
Clone-1   1081 ATVRKVLSMPQVNIVKKTEVQTGGFSKESILPKRNSDKLIARKKDWDPKKYGGFDSPTVA
Clone-2   1081 ATVRKVLSMPQVNIVKKTEVQTGGFSKESILPKRNSDKLIARKKDWDPKKYGGFDSPTVA
Clone-3   1081 ATVRKVLSMPQVNIVKKTEVQPGGFSKESILPKRNSDKLIARKKDWEPKKYGGFDSPTVA
WT-Cas9   1081 ATVRKVLSMPQVNIVKKTEVQTGGFSKESILPKRNSDKLIARKKDWDPKKYGGFDSPTVA
eSpCas9   1081 ATVRKVLSMPQVNIVKKTEVQTGGFSKESILPKRNSDKLIARKKDWDPKKYGGFDSPTVA
Cas9-HF1  1081 ATVRKVLSMPQVNIVKKTEVQTGGFSKESILPKRNSDKLIARKKDWDPKKYGGFDSPTVA
evo-Cas9  1081 ATVRKVLSMPQVNIVKKTEVQTGGFSKESILPKRNSDKLIARKKDWDPKKYGGFDSPTVA
Hypa-Cas9 1081 ATVRKVLSMPQVNIVKKTEVQTGGFSKESILPKRNSDKLIARKKDWDPKKYGGFDSPTVA
xCas9-3.7 1081 ATVRKVLSMPQVNIVKKTEVQTGGFSKESILPKRNSDKLIARKKDWDPKKYGGFDSPTVA

Clone-1   1141 YSVLVVAKVEKGKSKKLKSVKELLGITIMERSSFEKNPIDFLEAKGYKEVKKDLIIKLPK
Clone-2   1141 YSVLVVAKVEKGKSKKLKSVKELLGITIMERSSFEKNPIDFLEAKGYKEVKKDLIIKLPK
Clone-3   1141 YSVLVVAKVEKGKSKKLKSVKELLGITIMERSSFEKNPIDFLEAKGYKEVKKDLIIKLPK
WT-Cas9   1141 YSVLVVAKVEKGKSKKLKSVKELLGITIMERSSFEKNPIDFLEAKGYKEVKKDLIIKLPK
eSpCas9   1141 YSVLVVAKVEKGKSKKLKSVKELLGITIMERSSFEKNPIDFLEAKGYKEVKKDLIIKLPK
Cas9-HF1  1141 YSVLVVAKVEKGKSKKLKSVKELLGITIMERSSFEKNPIDFLEAKGYKEVKKDLIIKLPK
evo-Cas9  1141 YSVLVVAKVEKGKSKKLKSVKELLGITIMERSSFEKNPIDFLEAKGYKEVKKDLIIKLPK
Hypa-Cas9 1141 YSVLVVAKVEKGKSKKLKSVKELLGITIMERSSFEKNPIDFLEAKGYKEVKKDLIIKLPK
xCas9-3.7 1141 YSVLVVAKVEKGKSKKLKSVKELLGITIMERSSFEKNPIDFLEAKGYKEVKKDLIIKLPK

Clone-1   1201 YSLFELENGRKRMLASAGELQKGNELALPSKYVNFLYLASHYEKLKGSPEDNEQKQLFVE
Clone-2   1201 YSLFELENGRKRMLASAGELQKGNELALPSKYVNFLYLASHYEKLKGSPEDNEQKQLFVE
Clone-3   1201 YSLFELENGRKRMLASAGELQKGNELALPSKYVNFLYLASHYEKLKGSPEDNEQKQLFVE
WT-Cas9   1201 YSLFELENGRKRMLASAGELQKGNELALPSKYVNFLYLASHYEKLKGSPEDNEQKQLFVE
eSpCas9   1201 YSLFELENGRKRMLASAGELQKGNELALPSKYVNFLYLASHYEKLKGSPEDNEQKQLFVE
Cas9-HF1  1201 YSLFELENGRKRMLASAGELQKGNELALPSKYVNFLYLASHYEKLKGSPEDNEQKQLFVE
evo-Cas9  1201 YSLFELENGRKRMLASAGELQKGNELALPSKYVNFLYLASHYEKLKGSPEDNEQKQLFVE
Hypa-Cas9 1201 YSLFELENGRKRMLASAGELQKGNELALPSKYVNFLYLASHYEKLKGSPEDNEQKQLFVE
xCas9-3.7 1201 YSLFELENGRKRMLASAGVLQKGNELALPSKYVNFLYLASHYEKLKGSPEDNEQKQLFVE

Clone-1   1261 QHKHYLDEIIEQISEFSKRVILADANLDKVLSAYNKHRDKPIREQAENIIHLFTLTNLGA
Clone-2   1261 QHKHYLDEIIEQISEFSKRVILADANLDKVLSAYNKHRDKPIREQAENIIHLFTLTNLGA
Clone-3   1261 QHKHYLDEIIEQISEFSKRVILADANLDKVLSAYNKHRDKPIREQAENIIHLFTLTNLGA
WT-Cas9   1261 QHKHYLDEIIEQISEFSKRVILADANLDKVLSAYNKHRDKPIREQAENIIHLFTLTNLGA
eSpCas9   1261 QHKHYLDEIIEQISEFSKRVILADANLDKVLSAYNKHRDKPIREQAENIIHLFTLTNLGA
Cas9-HF1  1261 QHKHYLDEIIEQISEFSKRVILADANLDKVLSAYNKHRDKPIREQAENIIHLFTLTNLGA
evo-Cas9  1261 QHKHYLDEIIEQISEFSKRVILADANLDKVLSAYNKHRDKPIREQAENIIHLFTLTNLGA
Hypa-Cas9 1261 QHKHYLDEIIEQISEFSKRVILADANLDKVLSAYNKHRDKPIREQAENIIHLFTLTNLGA
xCas9-3.7 1261 QHKHYLDEIIEQISEFSKRVILADANLDKVLSAYNKHRDKPIREQAENIIHLFTLTNLGA

Clone-1   1321 PAAFKYFDTTIDRKRYTSTKEVLDATLIHQSITGLYETRIDLSQLGGDGGSGPPKKKRKV
Clone-2   1321 PAAFKYFDTTIDRKRYTSTKEVLDATLIHQSITGLYETRIDLSQLGGDGGSGPPKKKRKV
Clone-3   1321 PAAFKYFDTTIDRKRYTSTKEVLDATLIHQSITGLYETRIDLSQLGGDGGSGPPKKKRKV
WT-Cas9   1321 PAAFKYFDTTIDRKRYTSTKEVLDATLIHQSITGLYETRIDLSQLGGDGGSGPPKKKRKV
eSpCas9   1321 PAAFKYFDTTIDRKRYTSTKEVLDATLIHQSITGLYETRIDLSQLGGDGGSGPPKKKRKV
Cas9-HF1  1321 PAAFKYFDTTIDRKRYTSTKEVLDATLIHQSITGLYETRIDLSQLGGDGGSGPPKKKRKV
evo-Cas9  1321 PAAFKYFDTTIDRKRYTSTKEVLDATLIHQSITGLYETRIDLSQLGGDGGSGPPKKKRKV
Hypa-Cas9 1321 PAAFKYFDTTIDRKRYTSTKEVLDATLIHQSITGLYETRIDLSQLGGDGGSGPPKKKRKV
xCas9-3.7 1321 PAAFKYFDTTIDRKRYTSTKEVLDATLIHQSITGLYETRIDLSQLGGDGGSGPPKKKRKV

Clone-1   1381 YPYDVPDYA
Clone-2   1381 YPYDVPDYA
Clone-3   1381 YPYDVPDYA
WT-Cas9   1381 YPYDVPDYA
eSpCas9   1381 YPYDVPDYA
Cas9-HF1  1381 YPYDVPDYA
evo-Cas9  1381 YPYDVPDYA
Hypa-Cas9 1381 YPYDVPDYA
xCas9-3.7 1381 YPYDVPDYA
```

DNA sequence alignment of Cas9 variants used in this study

```
Clone-1      1  ATGGACAAGAAGTACAGCATCGGCCTGGACATCGGTACCAACAGCGTGGGCTGGGCCGTG
Clone-2      1  ATGGACAAGAAGTACAGCATCGGCCTGGACATCGGTACCAACAGCGTGGGCTGGGCCGTG
Clone-3      1  ATGGACAAGAAGTACAGCATCGGCCTGGACATCGGTACCAACAGCGTGGGCTGGGCCGTG
WT-Cas9      1  ATGGACAAGAAGTACAGCATCGGCCTGGACATCGGTACCAACAGCGTGGGCTGGGCCGTG
eSpCas9      1  ATGGACAAGAAGTACAGCATCGGCCTGGACATCGGTACCAACAGCGTGGGCTGGGCCGTG
Cas9-HF1     1  ATGGACAAGAAGTACAGCATCGGCCTGGACATCGGTACCAACAGCGTGGGCTGGGCCGTG
evo-Cas9     1  ATGGACAAGAAGTACAGCATCGGCCTGGACATCGGTACCAACAGCGTGGGCTGGGCCGTG
Hypa-Cas9    1  ATGGACAAGAAGTACAGCATCGGCCTGGACATCGGTACCAACAGCGTGGGCTGGGCCGTG
xCas9-3.7    1  ATGGACAAGAAGTACAGCATCGGCCTGGACATCGGTACCAACAGCGTGGGCTGGGCCGTG

Clone-1     61  ATCACCGACGAGTACAAGGTGCCCAGCAAGAAGTTCAAGGTGCTGGGCAACACCGACCGC
Clone-2     61  ATCACCGACGAGTACAAGGTGCCCAGCAAGAAGTTCAAGGTGCTGGGCAACACCGACCGC
Clone-3     61  ATCACCGACGAGTACAAGGTGCCCAGCAAGAAGTTCAAGGTGCTGGGCAACACCGACCGC
WT-Cas9     61  ATCACCGACGAGTACAAGGTGCCCAGCAAGAAGTTCAAGGTGCTGGGCAACACCGACCGC
eSpCas9     61  ATCACCGACGAGTACAAGGTGCCCAGCAAGAAGTTCAAGGTGCTGGGCAACACCGACCGC
Cas9-HF1    61  ATCACCGACGAGTACAAGGTGCCCAGCAAGAAGTTCAAGGTGCTGGGCAACACCGACCGC
evo-Cas9    61  ATCACCGACGAGTACAAGGTGCCCAGCAAGAAGTTCAAGGTGCTGGGCAACACCGACCGC
Hypa-Cas9   61  ATCACCGACGAGTACAAGGTGCCCAGCAAGAAGTTCAAGGTGCTGGGCAACACCGACCGC
xCas9-3.7   61  ATCACCGACGAGTACAAGGTGCCCAGCAAGAAGTTCAAGGTGCTGGGCAACACCGACCGC

Clone-1    121  CACAGCATCAAGAAGAACCTGATCGGCGCCCTGCTGTTCGACAGCGGCGAGACCGCCGAG
Clone-2    121  CACAGCATCAAGAAGAACCTGATCGGCGCCCTGCTGTTCGACAGCGGCGAGACCGCCGAG
Clone-3    121  CACAGCATCAAGAAGAACCTGATCGGCGCCCTGCTGTTCGACAGCGGCGAGACCGCCGAG
WT-Cas9    121  CACAGCATCAAGAAGAACCTGATCGGCGCCCTGCTGTTCGACAGCGGCGAGACCGCCGAG
eSpCas9    121  CACAGCATCAAGAAGAACCTGATCGGCGCCCTGCTGTTCGACAGCGGCGAGACCGCCGAG
Cas9-HF1   121  CACAGCATCAAGAAGAACCTGATCGGCGCCCTGCTGTTCGACAGCGGCGAGACCGCCGAG
evo-Cas9   121  CACAGCATCAAGAAGAACCTGATCGGCGCCCTGCTGTTCGACAGCGGCGAGACCGCCGAG
Hypa-Cas9  121  CACAGCATCAAGAAGAACCTGATCGGCGCCCTGCTGTTCGACAGCGGCGAGACCGCCGAG
xCas9-3.7  121  CACAGCATCAAGAAGAACCTGATCGGCGCCCTGCTGTTCGACAGCGGCGAGACCGCCGAG

Clone-1    181  GCCACCCGCCTGAAGCGCACCGCCCGCCGCCGCTACACCCGCCGCAAGAACCGCATCTGC
Clone-2    181  GCCACCCGCCTGAAGCGCACCGCCCGCCGCCGCTACACCCGCCGCAAGAACCGCATCTGC
Clone-3    181  GCCACCCGCCTGAAGCGCACCGCCCGCCGCCGCTACACCCGCCGCAAGAACCGCATCTGC
WT-Cas9    181  GCCACCCGCCTGAAGCGCACCGCCCGCCGCCGCTACACCCGCCGCAAGAACCGCATCTGC
eSpCas9    181  GCCACCCGCCTGAAGCGCACCGCCCGCCGCCGCTACACCCGCCGCAAGAACCGCATCTGC
Cas9-HF1   181  GCCACCCGCCTGAAGCGCACCGCCCGCCGCCGCTACACCCGCCGCAAGAACCGCATCTGC
evo-Cas9   181  GCCACCCGCCTGAAGCGCACCGCCCGCCGCCGCTACACCCGCCGCAAGAACCGCATCTGC
Hypa-Cas9  181  GCCACCCGCCTGAAGCGCACCGCCCGCCGCCGCTACACCCGCCGCAAGAACCGCATCTGC
xCas9-3.7  181  GCCACCCGCCTGAAGCGCACCGCCCGCCGCCGCTACACCCGCCGCAAGAACCGCATCTGC

Clone-1    241  TACCTGCAGGAGATCTTCAGCAACGAGATGGCCAAGGTGGACGACAGCTTCTTCCACCGC
Clone-2    241  TACCTGCAGGAGATCTTCAGCAACGAGATGGCCAAGGTGGACGACAGCTTCTTCCACCGC
Clone-3    241  TACCTGCAGGAGATCTTCAGCAACGAGATGGCCAAGGTGGACGACAGCTTCTTCCACCGC
WT-Cas9    241  TACCTGCAGGAGATCTTCAGCAACGAGATGGCCAAGGTGGACGACAGCTTCTTCCACCGC
eSpCas9    241  TACCTGCAGGAGATCTTCAGCAACGAGATGGCCAAGGTGGACGACAGCTTCTTCCACCGC
Cas9-HF1   241  TACCTGCAGGAGATCTTCAGCAACGAGATGGCCAAGGTGGACGACAGCTTCTTCCACCGC
evo-Cas9   241  TACCTGCAGGAGATCTTCAGCAACGAGATGGCCAAGGTGGACGACAGCTTCTTCCACCGC
Hypa-Cas9  241  TACCTGCAGGAGATCTTCAGCAACGAGATGGCCAAGGTGGACGACAGCTTCTTCCACCGC
xCas9-3.7  241  TACCTGCAGGAGATCTTCAGCAACGAGATGGCCAAGGTGGACGACAGCTTCTTCCACCGC

Clone-1    301  CTGGAGGAGAGCTTCCTGGTGGAGGAGGACAAGAAGCACGAGCGCCACCCCATCTTCGGC
Clone-2    301  CTGGAGGAGAGCTTCCTGGTGGAGGAGGACAAGAAGCACGAGCGCCACCCCATCTTCGGC
Clone-3    301  CTGGAGGAGAGCTTCCTGGTGGAGGAGGACAAGAAGCACGAGCGCCACCCCATCTTCGGC
WT-Cas9    301  CTGGAGGAGAGCTTCCTGGTGGAGGAGGACAAGAAGCACGAGCGCCACCCCATCTTCGGC
eSpCas9    301  CTGGAGGAGAGCTTCCTGGTGGAGGAGGACAAGAAGCACGAGCGCCACCCCATCTTCGGC
Cas9-HF1   301  CTGGAGGAGAGCTTCCTGGTGGAGGAGGACAAGAAGCACGAGCGCCACCCCATCTTCGGC
evo-Cas9   301  CTGGAGGAGAGCTTCCTGGTGGAGGAGGACAAGAAGCACGAGCGCCACCCCATCTTCGGC
Hypa-Cas9  301  CTGGAGGAGAGCTTCCTGGTGGAGGAGGACAAGAAGCACGAGCGCCACCCCATCTTCGGC
xCas9-3.7  301  CTGGAGGAGAGCTTCCTGGTGGAGGAGGACAAGAAGCACGAGCGCCACCCCATCTTCGGC
```

```
Clone-1     361 AACATCGTGGACGAGGTGGCCTACCACGAGAAGTACCCCACCATCTACCACCTGCGCAAG
Clone-2     361 AACATCGTGGACGAGGTGGCCTACCACGAGAAGTACCCCACCATCTACCACCTGCGCAAG
Clone-3     361 AACATCGTGGACGAGGTGGCCTACCACGAGAAGTACCCCACCATCTACCACCTGCGCAAG
WT-Cas9     361 AACATCGTGGACGAGGTGGCCTACCACGAGAAGTACCCCACCATCTACCACCTGCGCAAG
eSpCas9     361 AACATCGTGGACGAGGTGGCCTACCACGAGAAGTACCCCACCATCTACCACCTGCGCAAG
Cas9-HF1    361 AACATCGTGGACGAGGTGGCCTACCACGAGAAGTACCCCACCATCTACCACCTGCGCAAG
evo-Cas9    361 AACATCGTGGACGAGGTGGCCTACCACGAGAAGTACCCCACCATCTACCACCTGCGCAAG
Hypa-Cas9   361 AACATCGTGGACGAGGTGGCCTACCACGAGAAGTACCCCACCATCTACCACCTGCGCAAG
xCas9-3.7   361 AACATCGTGGACGAGGTGGCCTACCACGAGAAGTACCCCACCATCTACCACCTGCGCAAG

Clone-1     421 AAGCTGGTGGACAGCACCGACAAGGCCGACCTGCGCCTGATCTACCTGGCCCTGGCCCAC
Clone-2     421 AAGCTGGTGGACAGCACCGACAAGGCCGACCTGCGCCTGATCTACCTGGCCCTGGCCCAC
Clone-3     421 AAGCTGGTGGACAGCACTGACAAGGCCGACCTGCGCCTGATCTACCTGGCCCTGGCCCAC
WT-Cas9     421 AAGCTGGTGGACAGCACCGACAAGGCCGACCTGCGCCTGATCTACCTGGCCCTGGCCCAC
eSpCas9     421 AAGCTGGTGGACAGCACCGACAAGGCCGACCTGCGCCTGATCTACCTGGCCCTGGCCCAC
Cas9-HF1    421 AAGCTGGTGGACAGCACCGACAAGGCCGACCTGCGCCTGATCTACCTGGCCCTGGCCCAC
evo-Cas9    421 AAGCTGGTGGACAGCACCGACAAGGCCGACCTGCGCCTGATCTACCTGGCCCTGGCCCAC
Hypa-Cas9   421 AAGCTGGTGGACAGCACCGACAAGGCCGACCTGCGCCTGATCTACCTGGCCCTGGCCCAC
xCas9-3.7   421 AAGCTGGTGGACAGCACCGACAAGGCCGACCTGCGCCTGATCTACCTGGCCCTGGCCCAC

Clone-1     481 ATGATCAAGTTCCGCGGCCACTTCCTGATCGAGGGCGACCTGAACCCCGACAACAGCGAC
Clone-2     481 ATGATCAAGTTCCGCGGCCACTTCCTGATCGAGGGCGACCTGAACCCCGACAACAGCGAC
Clone-3     481 ATGATCAAGTTCCGCGGCCACTTCCTGATCGAGGGCGACCTGAACCCCGACAACAGCGAC
WT-Cas9     481 ATGATCAAGTTCCGCGGCCACTTCCTGATCGAGGGCGACCTGAACCCCGACAACAGCGAC
eSpCas9     481 ATGATCAAGTTCCGCGGCCACTTCCTGATCGAGGGCGACCTGAACCCCGACAACAGCGAC
Cas9-HF1    481 ATGATCAAGTTCCGCGGCCACTTCCTGATCGAGGGCGACCTGAACCCCGACAACAGCGAC
evo-Cas9    481 ATGATCAAGTTCCGCGGCCACTTCCTGATCGAGGGCGACCTGAACCCCGACAACAGCGAC
Hypa-Cas9   481 ATGATCAAGTTCCGCGGCCACTTCCTGATCGAGGGCGACCTGAACCCCGACAACAGCGAC
xCas9-3.7   481 ATGATCAAGTTCCGCGGCCACTTCCTGATCGAGGGCGACCTGAACCCCGACAACAGCGAC

Clone-1     541 GTGGACAAGCTGTTCATCCAGCTGGTGCAGACCTACAACCAGCTGTTCGAGGAGAACCCC
Clone-2     541 GTGGACAAGCTGTTTATCCAGCTGGTGCAGACCTACAACCAGCTGTTCGAGGAGAACCCC
Clone-3     541 GTGGACAAGCTGTTCATCCAGCTGGTGCAGACCTACAACCAGCTGTTCGAGGAGAACCCC
WT-Cas9     541 GTGGACAAGCTGTTCATCCAGCTGGTGCAGACCTACAACCAGCTGTTCGAGGAGAACCCC
eSpCas9     541 GTGGACAAGCTGTTCATCCAGCTGGTGCAGACCTACAACCAGCTGTTCGAGGAGAACCCC
Cas9-HF1    541 GTGGACAAGCTGTTCATCCAGCTGGTGCAGACCTACAACCAGCTGTTCGAGGAGAACCCC
evo-Cas9    541 GTGGACAAGCTGTTCATCCAGCTGGTGCAGACCTACAACCAGCTGTTCGAGGAGAACCCC
Hypa-Cas9   541 GTGGACAAGCTGTTCATCCAGCTGGTGCAGACCTACAACCAGCTGTTCGAGGAGAACCCC
xCas9-3.7   541 GTGGACAAGCTGTTCATCCAGCTGGTGCAGACCTACAACCAGCTGTTCGAGGAGAACCCC

Clone-1     601 ATCAACGCCAGCGGCGTGGACGCCAAGGCCATCCTGAGCGCCCGCCTGAGCAAGAGCCGC
Clone-2     601 ATCAACGCCAGCGGCGTGGACGCCAAGGCCATCCTGAGCGCCCGCCTGAGCAAGAGCCGC
Clone-3     601 ATCAACGACAGCGGCGTGGACGCCAAGGCCATCCTGAGCGCCCGCCTGAGCAAGAGCCGC
WT-Cas9     601 ATCAACGCCAGCGGCGTGGACGCCAAGGCCATCCTGAGCGCCCGCCTGAGCAAGAGCCGC
eSpCas9     601 ATCAACGCCAGCGGCGTGGACGCCAAGGCCATCCTGAGCGCCCGCCTGAGCAAGAGCCGC
Cas9-HF1    601 ATCAACGCCAGCGGCGTGGACGCCAAGGCCATCCTGAGCGCCCGCCTGAGCAAGAGCCGC
evo-Cas9    601 ATCAACGCCAGCGGCGTGGACGCCAAGGCCATCCTGAGCGCCCGCCTGAGCAAGAGCCGC
Hypa-Cas9   601 ATCAACGCCAGCGGCGTGGACGCCAAGGCCATCCTGAGCGCCCGCCTGAGCAAGAGCCGC
xCas9-3.7   601 ATCAACGCCAGCGGCGTGGACGCCAAGGCCATCCTGAGCGCCCGCCTGAGCAAGAGCCGC

Clone-1     661 CGCCTGGAGAACCTGATCGCCCAGCTGCCCGGCGAGAAGAAGAACGGCCTGTTCGGCAAC
Clone-2     661 CGCCTGGAGAACCTGATCGCCCAGCTGCCCGGCGAGAAGAAGAACGGCCTGTTCGGCAAC
Clone-3     661 CGCCTGGAGAACCTGATCGCCCAGCTGCCCGGCGAGAAGAAGAACGGCCTGTTCGGCAAC
WT-Cas9     661 CGCCTGGAGAACCTGATCGCCCAGCTGCCCGGCGAGAAGAAGAACGGCCTGTTCGGCAAC
eSpCas9     661 CGCCTGGAGAACCTGATCGCCCAGCTGCCCGGCGAGAAGAAGAACGGCCTGTTCGGCAAC
Cas9-HF1    661 CGCCTGGAGAACCTGATCGCCCAGCTGCCCGGCGAGAAGAAGAACGGCCTGTTCGGCAAC
evo-Cas9    661 CGCCTGGAGAACCTGATCGCCCAGCTGCCCGGCGAGAAGAAGAACGGCCTGTTCGGCAAC
Hypa-Cas9   661 CGCCTGGAGAACCTGATCGCCCAGCTGCCCGGCGAGAAGAAGAACGGCCTGTTCGGCAAC
xCas9-3.7   661 CGCCTGGAGAACCTGATCGCCCAGCTGCCCGGCGAGAAGAAGAACGGCCTGTTCGGCAAC
```

```
Clone-1    721 CTGATCGCCCTGAGCCTGGGCCTGACCCCCAACTTCAAGAGCAACTTCGACCTGGCCGAG
Clone-2    721 CTGATCGCCCTGAGCCTGGGCCTGACCCCCAACTTCAAGAGCAACTTCGACCTGGCCGAG
Clone-3    721 CTGATCGCCCTGAGCCTGGGCCTGACCCCCAACTTCAAGAGCAACTTCGACCTGGCCGAG
WT-Cas9    721 CTGATCGCCCTGAGCCTGGGCCTGACCCCCAACTTCAAGAGCAACTTCGACCTGGCCGAG
eSpCas9    721 CTGATCGCCCTGAGCCTGGGCCTGACCCCCAACTTCAAGAGCAACTTCGACCTGGCCGAG
Cas9-HF1   721 CTGATCGCCCTGAGCCTGGGCCTGACCCCCAACTTCAAGAGCAACTTCGACCTGGCCGAG
evo-Cas9   721 CTGATCGCCCTGAGCCTGGGCCTGACCCCCAACTTCAAGAGCAACTTCGACCTGGCCGAG
Hypa-Cas9  721 CTGATCGCCCTGAGCCTGGGCCTGACCCCCAACTTCAAGAGCAACTTCGACCTGGCCGAG
xCas9-3.7  721 CTGATCGCCCTGAGCCTGGGCCTGACCCCCAACTTCAAGAGCAACTTCGACCTGGCCGAG

Clone-1    781 GACGCCAAGCTGCAGCTGAGCAAGGACACCTACGACGACGACCTGGACAACCTGCTGGCC
Clone-2    781 GACGCCAAGCTGCAGCTGAGCAAGGACACCTACGACGACGACCTGGACAACCTGCTGGCC
Clone-3    781 GACGCCAAGCTGCAGCTGAGCAAGGACACCTACGACGACGACCTGGACCACCTGCTGGCT
WT-Cas9    781 GACGCCAAGCTGCAGCTGAGCAAGGACACCTACGACGACGACCTGGACAACCTGCTGGCC
eSpCas9    781 GACGCCAAGCTGCAGCTGAGCAAGGACACCTACGACGACGACCTGGACAACCTGCTGGCC
Cas9-HF1   781 GACGCCAAGCTGCAGCTGAGCAAGGACACCTACGACGACGACCTGGACAACCTGCTGGCC
evo-Cas9   781 GACGCCAAGCTGCAGCTGAGCAAGGACACCTACGACGACGACCTGGACAACCTGCTGGCC
Hypa-Cas9  781 GACGCCAAGCTGCAGCTGAGCAAGGACACCTACGACGACGACCTGGACAACCTGCTGGCC
xCas9-3.7  781 GACACCAAGCTGCAGCTGAGCAAGGACACCTACGACGACGACCTGGACAACCTGCTGGCC

Clone-1    841 CAGATCGGCGACCAGTACGCCGACCTGTTCCTGGCCGCCAAGAACCTGAGCGACGCCATC
Clone-2    841 CAGATCGGCGACCAGTACGCCGACCTGTTCCTGGCCGCCAAGAACCTGAGCGACGCCATC
Clone-3    841 CAGATCGGCGACCAGTACGCCGACCTGTTCCTGGCCGCCAAGAACCTGAGCGACGCCATC
WT-Cas9    841 CAGATCGGCGACCAGTACGCCGACCTGTTCCTGGCCGCCAAGAACCTGAGCGACGCCATC
eSpCas9    841 CAGATCGGCGACCAGTACGCCGACCTGTTCCTGGCCGCCAAGAACCTGAGCGACGCCATC
Cas9-HF1   841 CAGATCGGCGACCAGTACGCCGACCTGTTCCTGGCCGCCAAGAACCTGAGCGACGCCATC
evo-Cas9   841 CAGATCGGCGACCAGTACGCCGACCTGTTCCTGGCCGCCAAGAACCTGAGCGACGCCATC
Hypa-Cas9  841 CAGATCGGCGACCAGTACGCCGACCTGTTCCTGGCCGCCAAGAACCTGAGCGACGCCATC
xCas9-3.7  841 CAGATCGGCGACCAGTACGCCGACCTGTTCCTGGCCGCCAAGAACCTGAGCGACGCCATC

Clone-1    901 CTGCTGAGCGACATCCTGCGCGTGAACACCGAGATCACCAAGGCCCCCCTGAGCGCCAGC
Clone-2    901 CTGCTGAGCGACATCCTGCGCGTGAACACCGAGATCACCAAGGCCCCCCTGAGCGCCAGC
Clone-3    901 CTGCTGAGCGACATCCTGCGCGTGAACACCGAGATCACCAAGGCCCCCCTGAGCGCCAGC
WT-Cas9    901 CTGCTGAGCGACATCCTGCGCGTGAACACCGAGATCACCAAGGCCCCCCTGAGCGCCAGC
eSpCas9    901 CTGCTGAGCGACATCCTGCGCGTGAACACCGAGATCACCAAGGCCCCCCTGAGCGCCAGC
Cas9-HF1   901 CTGCTGAGCGACATCCTGCGCGTGAACACCGAGATCACCAAGGCCCCCCTGAGCGCCAGC
evo-Cas9   901 CTGCTGAGCGACATCCTGCGCGTGAACACCGAGATCACCAAGGCCCCCCTGAGCGCCAGC
Hypa-Cas9  901 CTGCTGAGCGACATCCTGCGCGTGAACACCGAGATCACCAAGGCCCCCCTGAGCGCCAGC
xCas9-3.7  901 CTGCTGAGCGACATCCTGCGCGTGAACACCGAGATCACCAAGGCCCCCCTGAGCGCCAGC

Clone-1    961 ATGATCAAGCGCTACGACGAGCACCACCAGGACCTGACCCTGCTGAAGGCCCTGGTGCGC
Clone-2    961 ATGATCAAGCGCTACGACGAGCACCACCAGGACCTGACCCTGCTGAAGGCCCTGGTGCGC
Clone-3    961 ATGATCAAGCGCTACGACGAGCACCACCAGGACCTGACCCTGCTGAAGGCCCTGGTGCGC
WT-Cas9    961 ATGATCAAGCGCTACGACGAGCACCACCAGGACCTGACCCTGCTGAAGGCCCTGGTGCGC
eSpCas9    961 ATGATCAAGCGCTACGACGAGCACCACCAGGACCTGACCCTGCTGAAGGCCCTGGTGCGC
Cas9-HF1   961 ATGATCAAGCGCTACGACGAGCACCACCAGGACCTGACCCTGCTGAAGGCCCTGGTGCGC
evo-Cas9   961 ATGATCAAGCGCTACGACGAGCACCACCAGGACCTGACCCTGCTGAAGGCCCTGGTGCGC
Hypa-Cas9  961 ATGATCAAGCGCTACGACGAGCACCACCAGGACCTGACCCTGCTGAAGGCCCTGGTGCGC
xCas9-3.7  961 ATGATCAAGCTGTACGACGAGCACCACCAGGACCTGACCCTGCTGAAGGCCCTGGTGCGC

Clone-1   1021 CAGCAGCTGCCCGAGAAGTACAAGGAGATCTTCTTCGACCAGAGCAAGAACGGCTACGCC
Clone-2   1021 CAGCAGCTGCCCGAGAAGTACAAGGAGATCTTCTTCGACCAGAGCAAGAACGGCTACGCC
Clone-3   1021 CAGCAGCTGCCCGAGAAGTACAAGGAGATCTTCTTCGACCAGAGCAAGAACGGCTACGCC
WT-Cas9   1021 CAGCAGCTGCCCGAGAAGTACAAGGAGATCTTCTTCGACCAGAGCAAGAACGGCTACGCC
eSpCas9   1021 CAGCAGCTGCCCGAGAAGTACAAGGAGATCTTCTTCGACCAGAGCAAGAACGGCTACGCC
Cas9-HF1  1021 CAGCAGCTGCCCGAGAAGTACAAGGAGATCTTCTTCGACCAGAGCAAGAACGGCTACGCC
evo-Cas9  1021 CAGCAGCTGCCCGAGAAGTACAAGGAGATCTTCTTCGACCAGAGCAAGAACGGCTACGCC
Hypa-Cas9 1021 CAGCAGCTGCCCGAGAAGTACAAGGAGATCTTCTTCGACCAGAGCAAGAACGGCTACGCC
xCas9-3.7 1021 CAGCAGCTGCCCGAGAAGTACAAGGAGATCTTCTTCGACCAGAGCAAGAACGGCTACGCC
```

```
Clone-1    1081 GGCTACATCGACGGCGGCGCCAGCCAGGAGGAGTTCTACAAGTTCATCAAGCCCATCCTG
Clone-2    1081 GGCTACATCGACGGCGGCGCCAGCCAGGAGGAGTTCTACAAGTTCATCAAGCCCATCCTG
Clone-3    1081 GGCTACATCGACGGCAGCGCCAGCCAGGAGGAGTTCTACAAGTTCATCAAGCCCATCCTG
WT-Cas9    1081 GGCTACATCGACGGCGGCGCCAGCCAGGAGGAGTTCTACAAGTTCATCAAGCCCATCCTG
eSpCas9    1081 GGCTACATCGACGGCGGCGCCAGCCAGGAGGAGTTCTACAAGTTCATCAAGCCCATCCTG
Cas9-HF1   1081 GGCTACATCGACGGCGGCGCCAGCCAGGAGGAGTTCTACAAGTTCATCAAGCCCATCCTG
evo-Cas9   1081 GGCTACATCGACGGCGGCGCCAGCCAGGAGGAGTTCTACAAGTTCATCAAGCCCATCCTG
Hypa-Cas9  1081 GGCTACATCGACGGCGGCGCCAGCCAGGAGGAGTTCTACAAGTTCATCAAGCCCATCCTG
xCas9-3.7  1081 GGCTACATCGACGGCGGCGCCAGCCAGGAGGAGTTCTACAAGTTCATCAAGCCCATCCTG

Clone-1    1141 GAGAAGATGGACGGCACCGAGGAGCTGCTGGTGAAGCTGAACCGCGAGGACCTGCTGCGC
Clone-2    1141 GAGAAGATGGACGGCACCGAGGAGCTGCTGGTGAAGCTGAACCGCGAGGACCTGCTGCGC
Clone-3    1141 GAGAAGATGGACGGCACCGAGGAGCTGCTGGTGAAGCTGAACCGCGAGGACCTGCTGCGC
WT-Cas9    1141 GAGAAGATGGACGGCACCGAGGAGCTGCTGGTGAAGCTGAACCGCGAGGACCTGCTGCGC
eSpCas9    1141 GAGAAGATGGACGGCACCGAGGAGCTGCTGGTGAAGCTGAACCGCGAGGACCTGCTGCGC
Cas9-HF1   1141 GAGAAGATGGACGGCACCGAGGAGCTGCTGGTGAAGCTGAACCGCGAGGACCTGCTGCGC
evo-Cas9   1141 GAGAAGATGGACGGCACCGAGGAGCTGCTGGTGAAGCTGAACCGCGAGGACCTGCTGCGC
Hypa-Cas9  1141 GAGAAGATGGACGGCACCGAGGAGCTGCTGGTGAAGCTGAACCGCGAGGACCTGCTGCGC
xCas9-3.7  1141 GAGAAGATGGACGGCACCGAGGAGCTGCTGGTGAAGCTGAACCGCGAGGACCTGCTGCGC

Clone-1    1201 AAGCAGCGCACCTTCGACAACGGCAGCATCCCCCACCAGATCCACCTGGGCGAGCTGCAC
Clone-2    1201 AAGCAGCGCACATTCGACAATGGCAGCATCCCCCACCAGATCCACCTGGGCGAGCTGCAC
Clone-3    1201 AAGCAGCGCACCTTCGACAACGGCAGCATCCCCCACCAGATCCACCTGGGCGAGCTGCAC
WT-Cas9    1201 AAGCAGCGCACCTTCGACAACGGCAGCATCCCCCACCAGATCCACCTGGGCGAGCTGCAC
eSpCas9    1201 AAGCAGCGCACCTTCGACAACGGCAGCATCCCCCACCAGATCCACCTGGGCGAGCTGCAC
Cas9-HF1   1201 AAGCAGCGCACCTTCGACAACGGCAGCATCCCCCACCAGATCCACCTGGGCGAGCTGCAC
evo-Cas9   1201 AAGCAGCGCACCTTCGACAACGGCAGCATCCCCCACCAGATCCACCTGGGCGAGCTGCAC
Hypa-Cas9  1201 AAGCAGCGCACCTTCGACAACGGCAGCATCCCCCACCAGATCCACCTGGGCGAGCTGCAC
xCas9-3.7  1201 AAGCAGCGCACCTTCGACAACGGCATCATCCCCCACCAGATCCACCTGGGCGAGCTGCAC

Clone-1    1261 GCCATCCTGCGCCGCCAGGAGGACTTCTACCCCTTCCTGAAGGACAACCGCGAGAAGATC
Clone-2    1261 GCCATCCTGCGCCGCCAGGAGGACTTCTACCCCTTCCTGAAGGACAACCGCGAGAAGATC
Clone-3    1261 GCCATCCTGCGCCGCCAGGAGGACTTCTACCCCTTCCTGAAGGACAACCGCGAGAAGATC
WT-Cas9    1261 GCCATCCTGCGCCGCCAGGAGGACTTCTACCCCTTCCTGAAGGACAACCGCGAGAAGATC
eSpCas9    1261 GCCATCCTGCGCCGCCAGGAGGACTTCTACCCCTTCCTGAAGGACAACCGCGAGAAGATC
Cas9-HF1   1261 GCCATCCTGCGCCGCCAGGAGGACTTCTACCCCTTCCTGAAGGACAACCGCGAGAAGATC
evo-Cas9   1261 GCCATCCTGCGCCGCCAGGAGGACTTCTACCCCTTCCTGAAGGACAACCGCGAGAAGATC
Hypa-Cas9  1261 GCCATCCTGCGCCGCCAGGAGGACTTCTACCCCTTCCTGAAGGACAACCGCGAGAAGATC
xCas9-3.7  1261 GCCATCCTGCGCCGCCAGGAGGACTTCTACCCCTTCCTGAAGGACAACCGCGAGAAGATC

Clone-1    1321 GAGAAGATCCTGACCTTCCGCATCCCCTACTACGTGGGCCCCCTGGCCCGCGGCAACAGC
Clone-2    1321 GAGAAGATCCTGACCTTCCGCATCCCCTACTACGTGGGCCCCCTGGCCCGCGGCAACAGC
Clone-3    1321 GAGAAGATCCTGACCTTCCGCATCCCCTACTACGTGGGCCCCCTGGCCCGCGGCAACAGC
WT-Cas9    1321 GAGAAGATCCTGACCTTCCGCATCCCCTACTACGTGGGCCCCCTGGCCCGCGGCAACAGC
eSpCas9    1321 GAGAAGATCCTGACCTTCCGCATCCCCTACTACGTGGGCCCCCTGGCCCGCGGCAACAGC
Cas9-HF1   1321 GAGAAGATCCTGACCTTCCGCATCCCCTACTACGTGGGCCCCCTGGCCCGCGGCAACAGC
evo-Cas9   1321 GAGAAGATCCTGACCTTCCGCATCCCCTACTACGTGGGCCCCCTGGCCCGCGGCAACAGC
Hypa-Cas9  1321 GAGAAGATCCTGACCTTCCGCATCCCCTACTACGTGGGCCCCCTGGCCCGCGGCAACAGC
xCas9-3.7  1321 GAGAAGATCCTGACCTTCCGCATCCCCTACTACGTGGGCCCCCTGGCCCGCGGCAACAGC

Clone-1    1381 CGCTTCGCCTGGATGACCCGCAAGAGCGAGGAGACCATCACCCCCTGGAACTTCGAGGAG
Clone-2    1381 CGCTTCGCCTGGATGACCCGCAAGAGCGAGGAGACCATCACCCCCTGGAACTTCGAGGAG
Clone-3    1381 CGCTTCGCCTGGATGACCCGCAAGAGCGAGGAGACCATCACCCCCTGGAACTTCGAGGAG
WT-Cas9    1381 CGCTTCGCCTGGATGACCCGCAAGAGCGAGGAGACCATCACCCCCTGGAACTTCGAGGAG
eSpCas9    1381 CGCTTCGCCTGGATGACCCGCAAGAGCGAGGAGACCATCACCCCCTGGAACTTCGAGGAG
Cas9-HF1   1381 CGCTTCGCCTGGATGACCCGCAAGAGCGAGGAGACCATCACCCCCTGGAACTTCGAGGAG
evo-Cas9   1381 CGCTTCGCCTGGATGACCCGCAAGAGCGAGGAGACCATCACCCCCTGGAACTTCGAGGAG
Hypa-Cas9  1381 CGCTTCGCCTGGATGACCCGCAAGAGCGAGGAGACCATCACCCCCTGGAACTTCGAGGAG
xCas9-3.7  1381 CGCTTCGCCTGGATGACCCGCAAGAGCGAGGAGACCATCACCCCCTGGAACTTCGAGAAG
```

```
Clone-1   1441 GTGGTGGACAAGGGCGCCAGCGCCCAGAGCTTCATCGAGCGCATGACCAACTTCGACAAG
Clone-2   1441 GTGGTGGACAAGGGCGCCAGCGCCCAGAGCTTCATCGAGCGCATGACCAACTTCGACAAG
Clone-3   1441 GTGGTGGACAAGGGCGCCAGCGCCCAGAGCTTCATCGAGCGCATGACCAACTTCGACAAG
WT-Cas9   1441 GTGGTGGACAAGGGCGCCAGCGCCCAGAGCTTCATCGAGCGCATGACCAACTTCGACAAG
eSpCas9   1441 GTGGTGGACAAGGGCGCCAGCGCCCAGAGCTTCATCGAGCGCATGACCAACTTCGACAAG
Cas9-HF1  1441 GTGGTGGACAAGGGCGCCAGCGCCCAGAGCTTCATCGAGCGCATGACCGCCTTCGACAAG
evo-Cas9  1441 GTGGTGGACAAGGGCGCCAGCGCCCAGAGCTTCATCGAGCGCGTGACCAACTTCGACAAG
Hypa-Cas9 1441 GTGGTGGACAAGGGCGCCAGCGCCCAGAGCTTCATCGAGCGCATGACCAACTTCGACAAG
xCas9-3.7 1441 GTGGTGGACAAGGGCGCCAGCGCCCAGAGCTTCATCGAGCGCATGACCAACTTCGACAAG

Clone-1   1501 AACCTGCCCAACGAGAAGGTGCTGCCCAAGCACAGCCTGCTGTACGAGTACTTCACCGTG
Clone-2   1501 AACCTGCCCAACGAGAAGGTGCTGCCCAAGCACAGCCTGCTGTACGAGTACTTCACCGTG
Clone-3   1501 AACCTGCCCAACGAGAAGGTGCTGCCCAAGCACAGCCTGCTGTACGAGTACTTCACCGTG
WT-Cas9   1501 AACCTGCCCAACGAGAAGGTGCTGCCCAAGCACAGCCTGCTGTACGAGTACTTCACCGTG
eSpCas9   1501 AACCTGCCCAACGAGAAGGTGCTGCCCAAGCACAGCCTGCTGTACGAGTACTTCACCGTG
Cas9-HF1  1501 AACCTGCCCAACGAGAAGGTGCTGCCCAAGCACAGCCTGCTGTACGAGTACTTCACCGTG
evo-Cas9  1501 AACCTGCCCAACGAGAAGGTGCTGCCCAAGCACAGCCTGCTGAACGAGTACTTCACCGTG
Hypa-Cas9 1501 AACCTGCCCAACGAGAAGGTGCTGCCCAAGCACAGCCTGCTGTACGAGTACTTCACCGTG
xCas9-3.7 1501 AACCTGCCCAACGAGAAGGTGCTGCCCAAGCACAGCCTGCTGTACGAGTACTTCACCGTG

Clone-1   1561 TACAACGAGCTGACCAAGGTGAAGTACGTGACCGAGGGCATGCGCAAGCCCGCCTCCCTG
Clone-2   1561 TACAACGAGCTGACCAAGGTGAAGTACGTGACCGAGGGCATGCGCAAGCCCGCCTTCCTG
Clone-3   1561 TACAACGAGCTGACCAAGGTGAAGTACGTGACCGAGGGCATGCGCAAGCCCGCCTTCCTG
WT-Cas9   1561 TACAACGAGCTGACCAAGGTGAAGTACGTGACCGAGGGCATGCGCAAGCCCGCCTTCCTG
eSpCas9   1561 TACAACGAGCTGACCAAGGTGAAGTACGTGACCGAGGGCATGCGCAAGCCCGCCTTCCTG
Cas9-HF1  1561 TACAACGAGCTGACCAAGGTGAAGTACGTGACCGAGGGCATGCGCAAGCCCGCCTTCCTG
evo-Cas9  1561 TACAACGAGCTGACCAAGGAGAAGTACGTGACCGAGGGCATGCGCAAGCCCGCCTTCCTG
Hypa-Cas9 1561 TACAACGAGCTGACCAAGGTGAAGTACGTGACCGAGGGCATGCGCAAGCCCGCCTTCCTG
xCas9-3.7 1561 TACAACGAGCTGACCAAGGTGAAGTACGTGACCGAGGGCATGCGCAAGCCCGCCTTCCTG

Clone-1   1621 AGCGGCGAGCAGAAGAAGGCCATCGTGGACCTGCTGTTCAAGACCAACCGCAAGGTGACC
Clone-2   1621 AGCGGCGAGCAGAAGAAGGCCATCGTGGACCTGCTGTTCAAGACCAACCGCAAGGTGACC
Clone-3   1621 AGCGGCGAGCAGAAGAAGGCCATCGTGGACCTGCTGTTCAAGACCAACCGCAAGGTGACC
WT-Cas9   1621 AGCGGCGAGCAGAAGAAGGCCATCGTGGACCTGCTGTTCAAGACCAACCGCAAGGTGACC
eSpCas9   1621 AGCGGCGAGCAGAAGAAGGCCATCGTGGACCTGCTGTTCAAGACCAACCGCAAGGTGACC
Cas9-HF1  1621 AGCGGCGAGCAGAAGAAGGCCATCGTGGACCTGCTGTTCAAGACCAACCGCAAGGTGACC
evo-Cas9  1621 AGCGGCGAGCAGAAGAAGGCCATCGTGGACCTGCTGTTCAAGACCAACCGCAAGGTGACC
Hypa-Cas9 1621 AGCGGCGAGCAGAAGAAGGCCATCGTGGACCTGCTGTTCAAGACCAACCGCAAGGTGACC
xCas9-3.7 1621 AGCGGCGACCAGAAGAAGGCCATCGTGGACCTGCTGTTCAAGACCAACCGCAAGGTGACC

Clone-1   1681 GTGAAGCAGCTGAAGGAGGACTACTTCAAGAAGATCGAGTGCTTCGACAGCGTGGAGATC
Clone-2   1681 GTGAAGCAGCTGAAGGAGGACTACTTCAAGAAGATCGAGTGCTTCGACAGCGTGGAGATC
Clone-3   1681 GTGAAGCAGCTGAAGGAGGACTACTTCAAGAAGATCGAGTGCTTCGACAGCGTGGAGATC
WT-Cas9   1681 GTGAAGCAGCTGAAGGAGGACTACTTCAAGAAGATCGAGTGCTTCGACAGCGTGGAGATC
eSpCas9   1681 GTGAAGCAGCTGAAGGAGGACTACTTCAAGAAGATCGAGTGCTTCGACAGCGTGGAGATC
Cas9-HF1  1681 GTGAAGCAGCTGAAGGAGGACTACTTCAAGAAGATCGAGTGCTTCGACAGCGTGGAGATC
evo-Cas9  1681 GTGAAGCAGCTGAAGGAGGACTACTTCAAGAAGATCGAGTGCTTCGACAGCGTGGAGATC
Hypa-Cas9 1681 GTGAAGCAGCTGAAGGAGGACTACTTCAAGAAGATCGAGTGCTTCGACAGCGTGGAGATC
xCas9-3.7 1681 GTGAAGCAGCTGAAGGAGGACTACTTCAAGAAGATCGAGTGCTTCGACAGCGTGGAGATC

Clone-1   1741 AGCGGCGTGGAGGACCGCTTCAACGCCAGCCTGGGCACCTACCACGACCTGCTGAAGATC
Clone-2   1741 AGCGGCGTGGAGGACCGCTTCAACGCCAGCCTGGGCACCTACCACGACCTGCTGAAGATC
Clone-3   1741 AGCGGCGTGGAGGACCGCTTCAACGCCAGCCTGGGCACCTACCACGACCTGCTGAAGATC
WT-Cas9   1741 AGCGGCGTGGAGGACCGCTTCAACGCCAGCCTGGGCACCTACCACGACCTGCTGAAGATC
eSpCas9   1741 AGCGGCGTGGAGGACCGCTTCAACGCCAGCCTGGGCACCTACCACGACCTGCTGAAGATC
Cas9-HF1  1741 AGCGGCGTGGAGGACCGCTTCAACGCCAGCCTGGGCACCTACCACGACCTGCTGAAGATC
evo-Cas9  1741 AGCGGCGTGGAGGACCGCTTCAACGCCAGCCTGGGCACCTACCACGACCTGCTGAAGATC
Hypa-Cas9 1741 AGCGGCGTGGAGGACCGCTTCAACGCCAGCCTGGGCACCTACCACGACCTGCTGAAGATC
xCas9-3.7 1741 AGCGGCGTGGAGGACCGCTTCAACGCCAGCCTGGGCACCTACCACGACCTGCTGAAGATC
```

```
Clone-1   1801 ATCAAGGACAAGGACTTCCTGGACAACGAGGAGAACGAGGACATCCTGGAGGACATCGTG
Clone-2   1801 AACAAGGACAAGGACTTCCTGGACAACGAGGAGAACGAGGACATCCTGGAGGACATCGTG
Clone-3   1801 ATCAAGGACAAGGACTTCCTGGACAACGAGGAGAACGAGGACATCCTGGAGGACATCGTG
WT-Cas9   1801 ATCAAGGACAAGGACTTCCTGGACAACGAGGAGAACGAGGACATCCTGGAGGACATCGTG
eSpCas9   1801 ATCAAGGACAAGGACTTCCTGGACAACGAGGAGAACGAGGACATCCTGGAGGACATCGTG
Cas9-HF1  1801 ATCAAGGACAAGGACTTCCTGGACAACGAGGAGAACGAGGACATCCTGGAGGACATCGTG
evo-Cas9  1801 ATCAAGGACAAGGACTTCCTGGACAACGAGGAGAACGAGGACATCCTGGAGGACATCGTG
Hypa-Cas9 1801 ATCAAGGACAAGGACTTCCTGGACAACGAGGAGAACGAGGACATCCTGGAGGACATCGTG
xCas9-3.7 1801 ATCAAGGACAAGGACTTCCTGGACAACGAGGAGAACGAGGACATCCTGGAGGACATCGTG

Clone-1   1861 CTGACCCTGACCCTGTTCGAGGACCGCGAGATGATCGAGGAGCGCCTGAAGACCTACGCC
Clone-2   1861 CTGACCCTGACCCTGTTCGAGGACCGTGAGATGATCGAGGAGCGCCTGAAGACCTACGCC
Clone-3   1861 TTGACCCTGACCCTGTTCGAGGACCGCGAGATGATCGAGGAGCGCCTGAAGACCTACGCC
WT-Cas9   1861 CTGACCCTGACCCTGTTCGAGGACCGCGAGATGATCGAGGAGCGCCTGAAGACCTACGCC
eSpCas9   1861 CTGACCCTGACCCTGTTCGAGGACCGCGAGATGATCGAGGAGCGCCTGAAGACCTACGCC
Cas9-HF1  1861 CTGACCCTGACCCTGTTCGAGGACCGCGAGATGATCGAGGAGCGCCTGAAGACCTACGCC
evo-Cas9  1861 CTGACCCTGACCCTGTTCGAGGACCGCGAGATGATCGAGGAGCGCCTGAAGACCTACGCC
Hypa-Cas9 1861 CTGACCCTGACCCTGTTCGAGGACCGCGAGATGATCGAGGAGCGCCTGAAGACCTACGCC
xCas9-3.7 1861 CTGACCCTGACCCTGTTCGAGGACCGCGAGATGATCGAGGAGCGCCTGAAGACCTACGCC

Clone-1   1921 CACCTGTTCGACGACAAGGTGATGAAGCAGCTGAAGCGCCGCCGCTACACCGGCTGGGGC
Clone-2   1921 CACCTGTTCGACGACAAGGTGATGAAGCAGCTGAAGCGCCGCCGCTACACCGGCTGGGGC
Clone-3   1921 CACCTGTTCGACGACAAGGTGATGAAGCAGCTGAAGCGCCGCCGCTACACCGGCTGGGGC
WT-Cas9   1921 CACCTGTTCGACGACAAGGTGATGAAGCAGCTGAAGCGCCGCCGCTACACCGGCTGGGGC
eSpCas9   1921 CACCTGTTCGACGACAAGGTGATGAAGCAGCTGAAGCGCCGCCGCTACACCGGCTGGGGC
Cas9-HF1  1921 CACCTGTTCGACGACAAGGTGATGAAGCAGCTGAAGCGCCGCCGCTACACCGGCTGGGGC
evo-Cas9  1921 CACCTGTTCGACGACAAGGTGATGAAGCAGCTGAAGCGCCGCCGCTACACCGGCTGGGGC
Hypa-Cas9 1921 CACCTGTTCGACGACAAGGTGATGAAGCAGCTGAAGCGCCGCCGCTACACCGGCTGGGGC
xCas9-3.7 1921 CACCTGTTCGACGACAAGGTGATGAAGCAGCTGAAGCGCCGCCGCTACACCGGCTGGGGC

Clone-1   1981 CGCCTGAGCCGCAAGCTTATCAACGGCATCCGCGACAAGCAGAGCGGCAAGACCATCCTG
Clone-2   1981 CGCCTGAGCCGCAAGCTTATCAACGGCATCCGCGACAAGCAGAGCGGCAAGACCATCCTG
Clone-3   1981 CGCCTGAGCCGCAAGCTTATCAACGGCATCCGCGACAAGCAGAGCGGCAAGACCATCCTG
WT-Cas9   1981 CGCCTGAGCCGCAAGCTTATCAACGGCATCCGCGACAAGCAGAGCGGCAAGACCATCCTG
eSpCas9   1981 CGCCTGAGCCGCAAGCTTATCAACGGCATCCGCGACAAGCAGAGCGGCAAGACCATCCTG
Cas9-HF1  1981 GCCCTGAGCCGCAAGCTTATCAACGGCATCCGCGACAAGCAGAGCGGCAAGACCATCCTG
evo-Cas9  1981 CAGCTGAGCCGCAAGCTTATCAACGGCATCCGCGACAAGCAGAGCGGCAAGACCATCCTG
Hypa-Cas9 1981 CGCCTGAGCCGCAAGCTTATCAACGGCATCCGCGACAAGCAGAGCGGCAAGACCATCCTG
xCas9-3.7 1981 CGCCTGAGCCGCAAGCTTATCAACGGCATCCGCGACAAGCAGAGCGGCAAGACCATCCTG

Clone-1   2041 GACTTCCTGAAGAGCGACGGCTTCGCCAACCGCAACTTCATGCAGCTGATCCACGACGAC
Clone-2   2041 GACTTCCTGAAGAGCGACGGCTTCGCCAACCGCAACTTCATGCAGCTGATCCACGACGAC
Clone-3   2041 GACTTCCTGAAGAGCGACGGCTTCGCCAACCGCAACTTCATGCAGCTGATCCACGACGAC
WT-Cas9   2041 GACTTCCTGAAGAGCGACGGCTTCGCCAACCGCAACTTCATGCAGCTGATCCACGACGAC
eSpCas9   2041 GACTTCCTGAAGAGCGACGGCTTCGCCAACCGCAACTTCATGCAGCTGATCCACGACGAC
Cas9-HF1  2041 GACTTCCTGAAGAGCGACGGCTTCGCCAACCGCAACTTCATGGCGCTGATCCACGACGAC
evo-Cas9  2041 GACTTCCTGAAGAGCGACGGCTTCGCCAACCGCAACTTCATGCAGCTGATCCACGACGAC
Hypa-Cas9 2041 GACTTCCTGAAGAGCGACGGCTTCGCCAACCGCGCCTTCGCGGCGCTGATCGCCGACGAC
xCas9-3.7 2041 GACTTCCTGAAGAGCGACGGCTTCGCCAACCGCAACTTCATCCAGCTGATCCACGACGAC

Clone-1   2101 AGCCTGACCTTCAAGGAGGACATCCAGAAGGCCCAGGTGAGCGGCCAGGGCGACAGCCTG
Clone-2   2101 AGCCTGACCTTCAAGGAGGACATCCAGAAGGCCCAGGTGAGCGGCCAGGGCGACAGCCTG
Clone-3   2101 AGCCTGACCTTCAAGGAGGACATCCAGAAGGCCCAGGTGAGCGGCCAGGGCGACAGCCTG
WT-Cas9   2101 AGCCTGACCTTCAAGGAGGACATCCAGAAGGCCCAGGTGAGCGGCCAGGGCGACAGCCTG
eSpCas9   2101 AGCCTGACCTTCAAGGAGGACATCCAGAAGGCCCAGGTGAGCGGCCAGGGCGACAGCCTG
Cas9-HF1  2101 AGCCTGACCTTCAAGGAGGACATCCAGAAGGCCCAGGTGAGCGGCCAGGGCGACAGCCTG
evo-Cas9  2101 AGCCTGACCTTCAAGGAGGACATCCAGAAGGCCCAGGTGAGCGGCCAGGGCGACAGCCTG
Hypa-Cas9 2101 AGCCTGACCTTCAAGGAGGACATCCAGAAGGCCCAGGTGAGCGGCCAGGGCGACAGCCTG
xCas9-3.7 2101 AGCCTGACCTTCAAGGAGGACATCCAGAAGGCCCAGGTGAGCGGCCAGGGCGACAGCCTG
```

```
Clone-1    2161 CACGAGCACATCGCCAACCTGGCCGGCAGCCCCGCCATCAAGAAGGGCATCCTGCAGACC
Clone-2    2161 CACGAGCACATCGCCAACCTGGCCGGCAGCCCCGCCATCAAGAAGGGCATCCTGCAGACC
Clone-3    2161 CACGAGCACATCGCCAACCTGGCCGGCAGCCCCGCCATCAAGAAGGGCATCCTGCAGACC
WT-Cas9    2161 CACGAGCACATCGCCAACCTGGCCGGCAGCCCCGCCATCAAGAAGGGCATCCTGCAGACC
eSpCas9    2161 CACGAGCACATCGCCAACCTGGCCGGCAGCCCCGCCATCAAGAAGGGCATCCTGCAGACC
Cas9-HF1   2161 CACGAGCACATCGCCAACCTGGCCGGCAGCCCCGCCATCAAGAAGGGCATCCTGCAGACC
evo-Cas9   2161 CACGAGCACATCGCCAACCTGGCCGGCAGCCCCGCCATCAAGAAGGGCATCCTGCAGACC
Hypa-Cas9  2161 CACGAGCACATCGCCAACCTGGCCGGCAGCCCCGCCATCAAGAAGGGCATCCTGCAGACC
xCas9-3.7  2161 CACGAGCACATCGCCAACCTGGCCGGCAGCCCCGCCATCAAGAAGGGCATCCTGCAGACC

Clone-1    2221 GTGAAGGTGGTGGACGAGCTGGTGAAGGTGATGGGCCGCCACAAGCCCGAGAACATCGTG
Clone-2    2221 GTGAAGGTGGTGGACGAGCTGGTGAAGGTGATGGGCCGCCACAAGCCCGAGAACATCGTG
Clone-3    2221 GTGAAGGTGGTGGACGAGCTGGTGAAGGTGATGGGCCGCCACAAGCCCGAGAACATCGTG
WT-Cas9    2221 GTGAAGGTGGTGGACGAGCTGGTGAAGGTGATGGGCCGCCACAAGCCCGAGAACATCGTG
eSpCas9    2221 GTGAAGGTGGTGGACGAGCTGGTGAAGGTGATGGGCCGCCACAAGCCCGAGAACATCGTG
Cas9-HF1   2221 GTGAAGGTGGTGGACGAGCTGGTGAAGGTGATGGGCCGCCACAAGCCCGAGAACATCGTG
evo-Cas9   2221 GTGAAGGTGGTGGACGAGCTGGTGAAGGTGATGGGCCGCCACAAGCCCGAGAACATCGTG
Hypa-Cas9  2221 GTGAAGGTGGTGGACGAGCTGGTGAAGGTGATGGGCCGCCACAAGCCCGAGAACATCGTG
xCas9-3.7  2221 GTGAAGGTGGTGGACGAGCTGGTGAAGGTGATGGGCCGCCACAAGCCCGAGAACATCGTG

Clone-1    2281 ATCGAGATAGCCCGCGAGAACCAGACCACCCAGAAGGGCCAGAAGAACAGCCGCGAGCGC
Clone-2    2281 ATCGAGATGGCCCGCGAGAACCAGACCACCCAGAAGGGCCAGAAGAACAGCCGCGAGCGC
Clone-3    2281 ATCGAGATAGCCCGCGAGAACCAGACCACCCAGAAGGGCCAGAAGAACAGCCGCGAGCGC
WT-Cas9    2281 ATCGAGATGGCCCGCGAGAACCAGACCACCCAGAAGGGCCAGAAGAACAGCCGCGAGCGC
eSpCas9    2281 ATCGAGATGGCCCGCGAGAACCAGACCACCCAGAAGGGCCAGAAGAACAGCCGCGAGCGC
Cas9-HF1   2281 ATCGAGATGGCCCGCGAGAACCAGACCACCCAGAAGGGCCAGAAGAACAGCCGCGAGCGC
evo-Cas9   2281 ATCGAGATGGCCCGCGAGAACCAGACCACCCAGAAGGGCCAGAAGAACAGCCGCGAGCGC
Hypa-Cas9  2281 ATCGAGATGGCCCGCGAGAACCAGACCACCCAGAAGGGCCAGAAGAACAGCCGCGAGCGC
xCas9-3.7  2281 ATCGAGATGGCCCGCGAGAACCAGACCACCCAGAAGGGCCAGAAGAACAGCCGCGAGCGC

Clone-1    2341 ATGAAGCGCATCGAGGAGGGCATCAAGGAGCTGGGCAGCCAGATCCTGAAGGAGCACCCC
Clone-2    2341 ATGAAGCGCATCGAGGAGGGCATCAAGGAGCTGGGCAGCCAGATCCTGAAGGAGCACCCC
Clone-3    2341 ATGAAGCGCATCGAGGAGGGCATCAAGGAGCTGGGCAGCCAGATCCTGAAGGAGCACCCC
WT-Cas9    2341 ATGAAGCGCATCGAGGAGGGCATCAAGGAGCTGGGCAGCCAGATCCTGAAGGAGCACCCC
eSpCas9    2341 ATGAAGCGCATCGAGGAGGGCATCAAGGAGCTGGGCAGCCAGATCCTGAAGGAGCACCCC
Cas9-HF1   2341 ATGAAGCGCATCGAGGAGGGCATCAAGGAGCTGGGCAGCCAGATCCTGAAGGAGCACCCC
evo-Cas9   2341 ATGAAGCGCATCGAGGAGGGCATCAAGGAGCTGGGCAGCCAGATCCTGAAGGAGCACCCC
Hypa-Cas9  2341 ATGAAGCGCATCGAGGAGGGCATCAAGGAGCTGGGCAGCCAGATCCTGAAGGAGCACCCC
xCas9-3.7  2341 ATGAAGCGCATCGAGGAGGGCATCAAGGAGCTGGGCAGCCAGATCCTGAAGGAGCACCCC

Clone-1    2401 GTGGAGAACACCCAGCTGCAGAACGAGAAGCTGTACCTGTACTACCTGCAGAACGGCCGC
Clone-2    2401 GTGGAGAACACCCAGCTGCAGAACGAGAAGCTGTACCTGTACTACCTGCAGAACGGCCGC
Clone-3    2401 GTGGAGAACACCCAGCTGCAGAACGAGAAGCTGTACCTGTACTACCTGCAGAACGGCCGC
WT-Cas9    2401 GTGGAGAACACCCAGCTGCAGAACGAGAAGCTGTACCTGTACTACCTGCAGAACGGCCGC
eSpCas9    2401 GTGGAGAACACCCAGCTGCAGAACGAGAAGCTGTACCTGTACTACCTGCAGAACGGCCGC
Cas9-HF1   2401 GTGGAGAACACCCAGCTGCAGAACGAGAAGCTGTACCTGTACTACCTGCAGAACGGCCGC
evo-Cas9   2401 GTGGAGAACACCCAGCTGCAGAACGAGAAGCTGTACCTGTACTACCTGCAGAACGGCCGC
Hypa-Cas9  2401 GTGGAGAACACCCAGCTGCAGAACGAGAAGCTGTACCTGTACTACCTGCAGAACGGCCGC
xCas9-3.7  2401 GTGGAGAACACCCAGCTGCAGAACGAGAAGCTGTACCTGTACTACCTGCAGAACGGCCGC

Clone-1    2461 GACATGTACGTGGACCAGGAGCTGGACATCAACCGCCTGAGCGACTACGACGTGGACCAC
Clone-2    2461 GACATGTACGTGGACCAGGAGCTGGACATCAACCGCCTGAGCGACTACGACGTGGACCAC
Clone-3    2461 GACATGTACGTGGACCAGGAGCTGGACATCAACCGCCTGAGCGACTACGACGTGGACCAC
WT-Cas9    2461 GACATGTACGTGGACCAGGAGCTGGACATCAACCGCCTGAGCGACTACGACGTGGACCAC
eSpCas9    2461 GACATGTACGTGGACCAGGAGCTGGACATCAACCGCCTGAGCGACTACGACGTGGACCAC
Cas9-HF1   2461 GACATGTACGTGGACCAGGAGCTGGACATCAACCGCCTGAGCGACTACGACGTGGACCAC
evo-Cas9   2461 GACATGTACGTGGACCAGGAGCTGGACATCAACCGCCTGAGCGACTACGACGTGGACCAC
Hypa-Cas9  2461 GACATGTACGTGGACCAGGAGCTGGACATCAACCGCCTGAGCGACTACGACGTGGACCAC
xCas9-3.7  2461 GACATGTACGTGGACCAGGAGCTGGACATCAACCGCCTGAGCGACTACGACGTGGACCAC
```

```
Clone-1   2521 ATCGTGCCCCAGAGCTTCCTGAAGGACGACAGCATCGACAACAAGGTGCTGACCCGCAGC
Clone-2   2521 ATCGTGCCCCAGAGCTTCCTGAAGGACGACAGCATCGACAACAAGGTGCTGACCCGCAGC
Clone-3   2521 ATCGTGCCCCAGAGCTTCCTGAAGGACGACAGCATCGACAACAAGGTGCTGACCCGCAGC
WT-Cas9   2521 ATCGTGCCCCAGAGCTTCCTGAAGGACGACAGCATCGACAACAAGGTGCTGACCCGCAGC
eSpCas9   2521 ATCGTGCCCCAGAGCTTCCTGGCGGACGACAGCATCGACAACAAGGTGCTGACCCGCAGC
Cas9-HF1  2521 ATCGTGCCCCAGAGCTTCCTGAAGGACGACAGCATCGACAACAAGGTGCTGACCCGCAGC
evo-Cas9  2521 ATCGTGCCCCAGAGCTTCCTGAAGGACGACAGCATCGACAACAAGGTGCTGACCCGCAGC
Hypa-Cas9 2521 ATCGTGCCCCAGAGCTTCCTGAAGGACGACAGCATCGACAACAAGGTGCTGACCCGCAGC
xCas9-3.7 2521 ATCGTGCCCCAGAGCTTCCTGAAGGACGACAGCATCGACAACAAGGTGCTGACCCGCAGC

Clone-1   2581 GACAAGAACCGCGGCAAGAGCGACAACGTGCCCAGCGAGGAGGTGGTGAAGAAGATGAAG
Clone-2   2581 GACAAGAACCGCGGCAAGAGCGACAACGTGCCCAGCGAGGAGGTGGTGAAGAAGATGAAG
Clone-3   2581 GACAAGAACCGCGGCAAGAGCGACAACGTGCCCAGCGAGGAGGTGGTGAAGAAGATGAAG
WT-Cas9   2581 GACAAGAACCGCGGCAAGAGCGACAACGTGCCCAGCGAGGAGGTGGTGAAGAAGATGAAG
eSpCas9   2581 GACAAGAACCGCGGCAAGAGCGACAACGTGCCCAGCGAGGAGGTGGTGAAGAAGATGAAG
Cas9-HF1  2581 GACAAGAACCGCGGCAAGAGCGACAACGTGCCCAGCGAGGAGGTGGTGAAGAAGATGAAG
evo-Cas9  2581 GACAAGAACCGCGGCAAGAGCGACAACGTGCCCAGCGAGGAGGTGGTGAAGAAGATGAAG
Hypa-Cas9 2581 GACAAGAACCGCGGCAAGAGCGACAACGTGCCCAGCGAGGAGGTGGTGAAGAAGATGAAG
xCas9-3.7 2581 GACAAGAACCGCGGCAAGAGCGACAACGTGCCCAGCGAGGAGGTGGTGAAGAAGATGAAG

Clone-1   2641 AACTACTGGCGCCAGCTGCTGAACGCCAATCTGATCACCCAGCGCAAGTTCGACAACCTG
Clone-2   2641 AACTACTGGCGCCAGCTGCTGAACGCCAAGCTGATCACCCAGCGCAAGTTCGACAACCTG
Clone-3   2641 AACTACTGGCGCCAGCTGCTGAACGCCAAGCTGATCACCCAGCGCAAGTTCGACAACCTG
WT-Cas9   2641 AACTACTGGCGCCAGCTGCTGAACGCCAAGCTGATCACCCAGCGCAAGTTCGACAACCTG
eSpCas9   2641 AACTACTGGCGCCAGCTGCTGAACGCCAAGCTGATCACCCAGCGCAAGTTCGACAACCTG
Cas9-HF1  2641 AACTACTGGCGCCAGCTGCTGAACGCCAAGCTGATCACCCAGCGCAAGTTCGACAACCTG
evo-Cas9  2641 AACTACTGGCGCCAGCTGCTGAACGCCAAGCTGATCACCCAGCGCAAGTTCGACAACCTG
Hypa-Cas9 2641 AACTACTGGCGCCAGCTGCTGAACGCCAAGCTGATCACCCAGCGCAAGTTCGACAACCTG
xCas9-3.7 2641 AACTACTGGCGCCAGCTGCTGAACGCCAAGCTGATCACCCAGCGCAAGTTCGACAACCTG

Clone-1   2701 ACCAAGGCCGAGCGCGGCGGCCTGAGCGAGCTGGACAAGGCCGGCTTCATCAAGCGCCAG
Clone-2   2701 ACCAAGGCCGAGCGCGGCGGCCTGAGCGAGCTGGACAAGGCCGGCTTCATCAAGCGCCAG
Clone-3   2701 ACCAAGGCCGAGCGCGGCGGCCTGAGCGAGCTGGACAAGGCCGGCTTCATCAAGCGCCAG
WT-Cas9   2701 ACCAAGGCCGAGCGCGGCGGCCTGAGCGAGCTGGACAAGGCCGGCTTCATCAAGCGCCAG
eSpCas9   2701 ACCAAGGCCGAGCGCGGCGGCCTGAGCGAGCTGGACAAGGCCGGCTTCATCAAGCGCCAG
Cas9-HF1  2701 ACCAAGGCCGAGCGCGGCGGCCTGAGCGAGCTGGACAAGGCCGGCTTCATCAAGCGCCAG
evo-Cas9  2701 ACCAAGGCCGAGCGCGGCGGCCTGAGCGAGCTGGACAAGGCCGGCTTCATCAAGCGCCAG
Hypa-Cas9 2701 ACCAAGGCCGAGCGCGGCGGCCTGAGCGAGCTGGACAAGGCCGGCTTCATCAAGCGCCAG
xCas9-3.7 2701 ACCAAGGCCGAGCGCGGCGGCCTGAGCGAGCTGGACAAGGCCGGCTTCATCAAGCGCCAG

Clone-1   2761 CTGGTGGAGACCCGCCAGATCACCAAGCACGTGGCCCAGATCCTGGACAGCCGCATGAAC
Clone-2   2761 CTGGTGGAGACCCGCCAGATCACCAAGCACGTGGCCCAGATCCTGGACAGCCGCATGAAC
Clone-3   2761 CTGGTGGAGACCCGCCAGATCACCAAGCACGTGGCCCAGATCCTGGACAGCCGCATGAAC
WT-Cas9   2761 CTGGTGGAGACCCGCCAGATCACCAAGCACGTGGCCCAGATCCTGGACAGCCGCATGAAC
eSpCas9   2761 CTGGTGGAGACCCGCCAGATCACCAAGCACGTGGCCCAGATCCTGGACAGCCGCATGAAC
Cas9-HF1  2761 CTGGTGGAGACCCGCGCGATCACCAAGCACGTGGCCCAGATCCTGGACAGCCGCATGAAC
evo-Cas9  2761 CTGGTGGAGACCCGCCAGATCACCAAGCACGTGGCCCAGATCCTGGACAGCCGCATGAAC
Hypa-Cas9 2761 CTGGTGGAGACCCGCCAGATCACCAAGCACGTGGCCCAGATCCTGGACAGCCGCATGAAC
xCas9-3.7 2761 CTGGTGGAGACCCGCCAGATCACCAAGCACGTGGCCCAGATCCTGGACAGCCGCATGAAC

Clone-1   2821 ACCAAGTACGACGAGAACGACAAGCTGATCCGCGAGGTGAAGGTGATCACCCTGAAGAGC
Clone-2   2821 ACCAAGTACGACGAGAACGACAAGCTGATCCGCGAGGTGAAGGTGATCACCCTGAAGAGC
Clone-3   2821 ACCAAGTACGACGAGAACGACAAGCTGATCCGCGAGGTGAAGGTGATCACCCTGAAGAGC
WT-Cas9   2821 ACCAAGTACGACGAGAACGACAAGCTGATCCGCGAGGTGAAGGTGATCACCCTGAAGAGC
eSpCas9   2821 ACCAAGTACGACGAGAACGACAAGCTGATCCGCGAGGTGAAGGTGATCACCCTGAAGAGC
Cas9-HF1  2821 ACCAAGTACGACGAGAACGACAAGCTGATCCGCGAGGTGAAGGTGATCACCCTGAAGAGC
evo-Cas9  2821 ACCAAGTACGACGAGAACGACAAGCTGATCCGCGAGGTGAAGGTGATCACCCTGAAGAGC
Hypa-Cas9 2821 ACCAAGTACGACGAGAACGACAAGCTGATCCGCGAGGTGAAGGTGATCACCCTGAAGAGC
xCas9-3.7 2821 ACCAAGTACGACGAGAACGACAAGCTGATCCGCGAGGTGAAGGTGATCACCCTGAAGAGC
```

```
Clone-1   2881 AAGCTGGTGAGCGACTTCCGCAAGGACTTCCAGTTCTACAAGGTGCGCGAGATCAACAAC
Clone-2   2881 AAGCTGGTGAGCTACTTCCGCAAGGACTTCCAGTTCTACAAGGTGCGCGAGATCAACAAC
Clone-3   2881 AAGCTGGTGAGCGACTTCCGCAAGGACTTCCAGTTCTACAAGGTGCGCGAGATCAACAAC
WT-Cas9   2881 AAGCTGGTGAGCGACTTCCGCAAGGACTTCCAGTTCTACAAGGTGCGCGAGATCAACAAC
eSpCas9   2881 AAGCTGGTGAGCGACTTCCGCAAGGACTTCCAGTTCTACAAGGTGCGCGAGATCAACAAC
Cas9-HF1  2881 AAGCTGGTGAGCGACTTCCGCAAGGACTTCCAGTTCTACAAGGTGCGCGAGATCAACAAC
evo-Cas9  2881 AAGCTGGTGAGCGACTTCCGCAAGGACTTCCAGTTCTACAAGGTGCGCGAGATCAACAAC
Hypa-Cas9 2881 AAGCTGGTGAGCGACTTCCGCAAGGACTTCCAGTTCTACAAGGTGCGCGAGATCAACAAC
xCas9-3.7 2881 AAGCTGGTGAGCGACTTCCGCAAGGACTTCCAGTTCTACAAGGTGCGCGAGATCAACAAC

Clone-1   2941 TACCACCACGCCCACGACGCCTACCTGAACGCCGTGGTGGGCACCGCCCTGATCAAGAAG
Clone-2   2941 TACCACCACGCCCACGACGCCTACCTGAACGCCGTGGTGGGCACCGCCCTGATCAAGAAG
Clone-3   2941 TACCACCACGCCCACGACGCCTACCTGAACGCCGTGGTGGGCACCGCCCTGATCAAGAAG
WT-Cas9   2941 TACCACCACGCCCACGACGCCTACCTGAACGCCGTGGTGGGCACCGCCCTGATCAAGAAG
eSpCas9   2941 TACCACCACGCCCACGACGCCTACCTGAACGCCGTGGTGGGCACCGCCCTGATCAAGAAG
Cas9-HF1  2941 TACCACCACGCCCACGACGCCTACCTGAACGCCGTGGTGGGCACCGCCCTGATCAAGAAG
evo-Cas9  2941 TACCACCACGCCCACGACGCCTACCTGAACGCCGTGGTGGGCACCGCCCTGATCAAGAAG
Hypa-Cas9 2941 TACCACCACGCCCACGACGCCTACCTGAACGCCGTGGTGGGCACCGCCCTGATCAAGAAG
xCas9-3.7 2941 TACCACCACGCCCACGACGCCTACCTGAACGCCGTGGTGGGCACCGCCCTGATCAAGAAG

Clone-1   3001 TACCCCAAGCTGGAGAGCGAGTTCGTGTACGGCGACTACAAGGTATACGACGTGCGCAAG
Clone-2   3001 TACCCCAAGCTGGAGAGCGAGTTCGTGTACGGCGACTACAAGGTGTACGACGTGCGCAAG
Clone-3   3001 TACCCCAAGCTGGAGAGCGAGTTCGTGTACGGCGACTACAAGGTGTACGACGTGCGCAAG
WT-Cas9   3001 TACCCCAAGCTGGAGAGCGAGTTCGTGTACGGCGACTACAAGGTGTACGACGTGCGCAAG
eSpCas9   3001 TACCCCGCGCTGGAGAGCGAGTTCGTGTACGGCGACTACAAGGTGTACGACGTGCGCAAG
Cas9-HF1  3001 TACCCCAAGCTGGAGAGCGAGTTCGTGTACGGCGACTACAAGGTGTACGACGTGCGCAAG
evo-Cas9  3001 TACCCCAAGCTGGAGAGCGAGTTCGTGTACGGCGACTACAAGGTGTACGACGTGCGCAAG
Hypa-Cas9 3001 TACCCCAAGCTGGAGAGCGAGTTCGTGTACGGCGACTACAAGGTGTACGACGTGCGCAAG
xCas9-3.7 3001 TACCCCAAGCTGGAGAGCGAGTTCGTGTACGGCGACTACAAGGTGTACGACGTGCGCAAG

Clone-1   3061 ATGATCGCTAAGAGCGAGCAGGAGATCGGCAAGGCCACCGCCAAGTACTTCTTCTACAGC
Clone-2   3061 ATGATCGCCAAGAGCGAGCAGGAGATCGGCAAGGCCACCGCCAAGTACTTCTTCTACAGC
Clone-3   3061 ATGATCGCCAAGAGCGAGCAGGAGATCGGCAAGGCCACCGCCAAGTACTTCTACTACAGC
WT-Cas9   3061 ATGATCGCCAAGAGCGAGCAGGAGATCGGCAAGGCCACCGCCAAGTACTTCTTCTACAGC
eSpCas9   3061 ATGATCGCCAAGAGCGAGCAGGAGATCGGCAAGGCCACCGCCAAGTACTTCTTCTACAGC
Cas9-HF1  3061 ATGATCGCCAAGAGCGAGCAGGAGATCGGCAAGGCCACCGCCAAGTACTTCTTCTACAGC
evo-Cas9  3061 ATGATCGCCAAGAGCGAGCAGGAGATCGGCAAGGCCACCGCCAAGTACTTCTTCTACAGC
Hypa-Cas9 3061 ATGATCGCCAAGAGCGAGCAGGAGATCGGCAAGGCCACCGCCAAGTACTTCTTCTACAGC
xCas9-3.7 3061 ATGATCGCCAAGAGCGAGCAGGAGATCGGCAAGGCCACCGCCAAGTACTTCTTCTACAGC

Clone-1   3121 AACATCATGAACTTCTTCAAGACCGAGATCACCCTGGCCAACGGCGAGATCCGCAAGCGC
Clone-2   3121 AACATCATGAACTTCTTCAAGACCGAGATCACCCTGGCCAACGGCGAGATCCGCAAGCGC
Clone-3   3121 AACATCATGAACTTCTTCAAGACCGAGATCACCCTGGCCAACGGCGAGATCCGCAAGCGC
WT-Cas9   3121 AACATCATGAACTTCTTCAAGACCGAGATCACCCTGGCCAACGGCGAGATCCGCAAGCGC
eSpCas9   3121 AACATCATGAACTTCTTCAAGACCGAGATCACCCTGGCCAACGGCGAGATCCGCAAGGCC
Cas9-HF1  3121 AACATCATGAACTTCTTCAAGACCGAGATCACCCTGGCCAACGGCGAGATCCGCAAGCGC
evo-Cas9  3121 AACATCATGAACTTCTTCAAGACCGAGATCACCCTGGCCAACGGCGAGATCCGCAAGCGC
Hypa-Cas9 3121 AACATCATGAACTTCTTCAAGACCGAGATCACCCTGGCCAACGGCGAGATCCGCAAGCGC
xCas9-3.7 3121 AACATCATGAACTTCTTCAAGACCGAGATCACCCTGGCCAACGGCGAGATCCGCAAGCGC

Clone-1   3181 CCCCTGATCGAGACCAACGGCGAGACCGGCGAGATCGTGTGGGACAAGGGCCGCGACTTC
Clone-2   3181 CCCCTGATCGAGACCAACGGCGAGACCGGCGAGATCGTGTGGGACAAGGGCCGCGACTTC
Clone-3   3181 CCCCTGATCGAGACCAACGGCGAGACCGGCGAGATCGTGTGGGACAAGGGCCGCGACTTC
WT-Cas9   3181 CCCCTGATCGAGACCAACGGCGAGACCGGCGAGATCGTGTGGGACAAGGGCCGCGACTTC
eSpCas9   3181 CCCCTGATCGAGACCAACGGCGAGACCGGCGAGATCGTGTGGGACAAGGGCCGCGACTTC
Cas9-HF1  3181 CCCCTGATCGAGACCAACGGCGAGACCGGCGAGATCGTGTGGGACAAGGGCCGCGACTTC
evo-Cas9  3181 CCCCTGATCGAGACCAACGGCGAGACCGGCGAGATCGTGTGGGACAAGGGCCGCGACTTC
Hypa-Cas9 3181 CCCCTGATCGAGACCAACGGCGAGACCGGCGAGATCGTGTGGGACAAGGGCCGCGACTTC
xCas9-3.7 3181 CCCCTGATCGAGACCAACGGCGAGACCGGCGAGATCGTGTGGGACAAGGGCCGCGACTTC
```

```
Clone-1    3241 GCCACCGTGCGCAAGGTGCTGAGCATGCCCCAGGTGAACATCGTGAAGAAGACCGAGGTG
Clone-2    3241 GCCACCGTGCGCAAGGTGCTGAGCATGCCCCAGGTGAACATCGTGAAGAAGACCGAGGTG
Clone-3    3241 GCCACCGTGCGCAAGGTGCTGAGCATGCCCCAGGTGAACATCGTGAAGAAGACCGAGGTG
WT-Cas9    3241 GCCACCGTGCGCAAGGTGCTGAGCATGCCCCAGGTGAACATCGTGAAGAAGACCGAGGTG
eSpCas9    3241 GCCACCGTGCGCAAGGTGCTGAGCATGCCCCAGGTGAACATCGTGAAGAAGACCGAGGTG
Cas9-HF1   3241 GCCACCGTGCGCAAGGTGCTGAGCATGCCCCAGGTGAACATCGTGAAGAAGACCGAGGTG
evo-Cas9   3241 GCCACCGTGCGCAAGGTGCTGAGCATGCCCCAGGTGAACATCGTGAAGAAGACCGAGGTG
Hypa-Cas9  3241 GCCACCGTGCGCAAGGTGCTGAGCATGCCCCAGGTGAACATCGTGAAGAAGACCGAGGTG
xCas9-3.7  3241 GCCACCGTGCGCAAGGTGCTGAGCATGCCCCAGGTGAACATCGTGAAGAAGACCGAGGTG

Clone-1    3301 CAGACCGGCGGCTTCAGCAAGGAGAGCATCCTGCCCAAGCGCAACAGCGACAAGCTGATC
Clone-2    3301 CAGACCGGCGGCTTCAGCAAGGAGAGCATCCTGCCCAAGCGCAACAGCGACAAGCTGATC
Clone-3    3301 CAGCCCGGCGGCTTCAGCAAGGAGAGCATTCTGCCCAAGCGCAACAGCGACAAGCTGATC
WT-Cas9    3301 CAGACCGGCGGCTTCAGCAAGGAGAGCATCCTGCCCAAGCGCAACAGCGACAAGCTGATC
eSpCas9    3301 CAGACCGGCGGCTTCAGCAAGGAGAGCATCCTGCCCAAGCGCAACAGCGACAAGCTGATC
Cas9-HF1   3301 CAGACCGGCGGCTTCAGCAAGGAGAGCATCCTGCCCAAGCGCAACAGCGACAAGCTGATC
evo-Cas9   3301 CAGACCGGCGGCTTCAGCAAGGAGAGCATCCTGCCCAAGCGCAACAGCGACAAGCTGATC
Hypa-Cas9  3301 CAGACCGGCGGCTTCAGCAAGGAGAGCATCCTGCCCAAGCGCAACAGCGACAAGCTGATC
xCas9-3.7  3301 CAGACCGGCGGCTTCAGCAAGGAGAGCATCCTGCCCAAGCGCAACAGCGACAAGCTGATC

Clone-1    3361 GCCCGCAAGAAGGACTGGGACCCCAAGAAGTACGGCGGCTTCGACAGCCCCACCGTGGCC
Clone-2    3361 GCCCGCAAGAAGGACTGGGACCCCAAGAAGTACGGCGGCTTCGACAGCCCCACCGTGGCC
Clone-3    3361 GCCCGCAAGAAGGACTGGGAACCCAAGAAGTACGGCGGCTTCGACAGCCCCACCGTGGCC
WT-Cas9    3361 GCCCGCAAGAAGGACTGGGACCCCAAGAAGTACGGCGGCTTCGACAGCCCCACCGTGGCC
eSpCas9    3361 GCCCGCAAGAAGGACTGGGACCCCAAGAAGTACGGCGGCTTCGACAGCCCCACCGTGGCC
Cas9-HF1   3361 GCCCGCAAGAAGGACTGGGACCCCAAGAAGTACGGCGGCTTCGACAGCCCCACCGTGGCC
evo-Cas9   3361 GCCCGCAAGAAGGACTGGGACCCCAAGAAGTACGGCGGCTTCGACAGCCCCACCGTGGCC
Hypa-Cas9  3361 GCCCGCAAGAAGGACTGGGACCCCAAGAAGTACGGCGGCTTCGACAGCCCCACCGTGGCC
xCas9-3.7  3361 GCCCGCAAGAAGGACTGGGACCCCAAGAAGTACGGCGGCTTCGACAGCCCCACCGTGGCC

Clone-1    3421 TACAGCGTGCTGGTGGTGGCCAAGGTGGAGAAGGGCAAGAGCAAGAAGCTGAAGAGCGTG
Clone-2    3421 TACAGCGTGCTGGTGGTGGCCAAGGTGGAGAAGGGCAAGAGCAAGAAGCTGAAGAGCGTG
Clone-3    3421 TACAGCGTGCTGGTGGTGGCCAAGGTGGAGAAGGGCAAGAGCAAGAAGCTGAAGAGCGTG
WT-Cas9    3421 TACAGCGTGCTGGTGGTGGCCAAGGTGGAGAAGGGCAAGAGCAAGAAGCTGAAGAGCGTG
eSpCas9    3421 TACAGCGTGCTGGTGGTGGCCAAGGTGGAGAAGGGCAAGAGCAAGAAGCTGAAGAGCGTG
Cas9-HF1   3421 TACAGCGTGCTGGTGGTGGCCAAGGTGGAGAAGGGCAAGAGCAAGAAGCTGAAGAGCGTG
evo-Cas9   3421 TACAGCGTGCTGGTGGTGGCCAAGGTGGAGAAGGGCAAGAGCAAGAAGCTGAAGAGCGTG
Hypa-Cas9  3421 TACAGCGTGCTGGTGGTGGCCAAGGTGGAGAAGGGCAAGAGCAAGAAGCTGAAGAGCGTG
xCas9-3.7  3421 TACAGCGTGCTGGTGGTGGCCAAGGTGGAGAAGGGCAAGAGCAAGAAGCTGAAGAGCGTG

Clone-1    3481 AAGGAGCTGCTGGGCATCACCATCATGGAGCGCAGCAGCTTCGAGAAGAACCCCATCGAC
Clone-2    3481 AAGGAGCTGCTGGGCATCACCATCATGGAGCGCAGCAGCTTCGAGAAGAACCCCATCGAC
Clone-3    3481 AAGGAGCTGCTGGGCATCACCATCATGGAGCGCAGCAGCTTCGAGAAGAACCCCATCGAC
WT-Cas9    3481 AAGGAGCTGCTGGGCATCACCATCATGGAGCGCAGCAGCTTCGAGAAGAACCCCATCGAC
eSpCas9    3481 AAGGAGCTGCTGGGCATCACCATCATGGAGCGCAGCAGCTTCGAGAAGAACCCCATCGAC
Cas9-HF1   3481 AAGGAGCTGCTGGGCATCACCATCATGGAGCGCAGCAGCTTCGAGAAGAACCCCATCGAC
evo-Cas9   3481 AAGGAGCTGCTGGGCATCACCATCATGGAGCGCAGCAGCTTCGAGAAGAACCCCATCGAC
Hypa-Cas9  3481 AAGGAGCTGCTGGGCATCACCATCATGGAGCGCAGCAGCTTCGAGAAGAACCCCATCGAC
xCas9-3.7  3481 AAGGAGCTGCTGGGCATCACCATCATGGAGCGCAGCAGCTTCGAGAAGAACCCCATCGAC

Clone-1    3541 TTCCTGGAGGCCAAGGGCTACAAGGAGGTGAAGAAGGACCTGATCATCAAGCTGCCCAAG
Clone-2    3541 TTCCTGGAGGCCAAGGGCTACAAGGAGGTGAAGAAGGACCTGATCATCAAGCTGCCCAAG
Clone-3    3541 TTCCTGGAGGCCAAGGGCTACAAGGAGGTGAAGAAGGACCTGATCATCAAGCTGCCCAAG
WT-Cas9    3541 TTCCTGGAGGCCAAGGGCTACAAGGAGGTGAAGAAGGACCTGATCATCAAGCTGCCCAAG
eSpCas9    3541 TTCCTGGAGGCCAAGGGCTACAAGGAGGTGAAGAAGGACCTGATCATCAAGCTGCCCAAG
Cas9-HF1   3541 TTCCTGGAGGCCAAGGGCTACAAGGAGGTGAAGAAGGACCTGATCATCAAGCTGCCCAAG
evo-Cas9   3541 TTCCTGGAGGCCAAGGGCTACAAGGAGGTGAAGAAGGACCTGATCATCAAGCTGCCCAAG
Hypa-Cas9  3541 TTCCTGGAGGCCAAGGGCTACAAGGAGGTGAAGAAGGACCTGATCATCAAGCTGCCCAAG
xCas9-3.7  3541 TTCCTGGAGGCCAAGGGCTACAAGGAGGTGAAGAAGGACCTGATCATCAAGCTGCCCAAG
```

```
Clone-1    3601 TACAGCCTGTTCGAGCTGGAGAACGGCCGCAAGCGCATGCTGGCCAGCGCCGGCGAGCTG
Clone-2    3601 TACAGCCTGTTCGAGCTGGAGAACGGCCGCAAGCGCATGCTGGCCAGCGCCGGCGAGCTG
Clone-3    3601 TACAGCCTGTTCGAGCTGGAGAACGGCCGCAAGCGCATGCTGGCCAGCGCCGGCGAGCTG
WT-Cas9    3601 TACAGCCTGTTCGAGCTGGAGAACGGCCGCAAGCGCATGCTGGCCAGCGCCGGCGAGCTG
eSpCas9    3601 TACAGCCTGTTCGAGCTGGAGAACGGCCGCAAGCGCATGCTGGCCAGCGCCGGCGAGCTG
Cas9-HF1   3601 TACAGCCTGTTCGAGCTGGAGAACGGCCGCAAGCGCATGCTGGCCAGCGCCGGCGAGCTG
evo-Cas9   3601 TACAGCCTGTTCGAGCTGGAGAACGGCCGCAAGCGCATGCTGGCCAGCGCCGGCGAGCTG
Hypa-Cas9  3601 TACAGCCTGTTCGAGCTGGAGAACGGCCGCAAGCGCATGCTGGCCAGCGCCGGCGAGCTG
xCas9-3.7  3601 TACAGCCTGTTCGAGCTGGAGAACGGCCGCAAGCGCATGCTGGCCAGCGCCGGCGTGCTG

Clone-1    3661 CAGAAGGGCAACGAGCTGGCCCTGCCCAGCAAGTACGTGAACTTCCTGTACCTGGCCAGC
Clone-2    3661 CAGAAGGGCAACGAGCTGGCCCTGCCCAGCAAGTACGTGAACTTCCTGTACCTGGCCAGC
Clone-3    3661 CAGAAGGGCAACGAGCTGGCCCTGCCCAGCAAGTACGTGAACTTCCTGTACCTGGCCAGC
WT-Cas9    3661 CAGAAGGGCAACGAGCTGGCCCTGCCCAGCAAGTACGTGAACTTCCTGTACCTGGCCAGC
eSpCas9    3661 CAGAAGGGCAACGAGCTGGCCCTGCCCAGCAAGTACGTGAACTTCCTGTACCTGGCCAGC
Cas9-HF1   3661 CAGAAGGGCAACGAGCTGGCCCTGCCCAGCAAGTACGTGAACTTCCTGTACCTGGCCAGC
evo-Cas9   3661 CAGAAGGGCAACGAGCTGGCCCTGCCCAGCAAGTACGTGAACTTCCTGTACCTGGCCAGC
Hypa-Cas9  3661 CAGAAGGGCAACGAGCTGGCCCTGCCCAGCAAGTACGTGAACTTCCTGTACCTGGCCAGC
xCas9-3.7  3661 CAGAAGGGCAACGAGCTGGCCCTGCCCAGCAAGTACGTGAACTTCCTGTACCTGGCCAGC

Clone-1    3721 CACTACGAGAAGCTGAAGGGCAGCCCCGAGGACAACGAGCAGAAGCAGCTGTTCGTGGAG
Clone-2    3721 CACTACGAGAAGCTGAAGGGCAGCCCCGAGGACAACGAGCAGAAGCAGCTGTTCGTGGAG
Clone-3    3721 CACTACGAGAAGCTGAAGGGCAGCCCCGAGGACAACGAGCAGAAGCAGCTGTTCGTGGAG
WT-Cas9    3721 CACTACGAGAAGCTGAAGGGCAGCCCCGAGGACAACGAGCAGAAGCAGCTGTTCGTGGAG
eSpCas9    3721 CACTACGAGAAGCTGAAGGGCAGCCCCGAGGACAACGAGCAGAAGCAGCTGTTCGTGGAG
Cas9-HF1   3721 CACTACGAGAAGCTGAAGGGCAGCCCCGAGGACAACGAGCAGAAGCAGCTGTTCGTGGAG
evo-Cas9   3721 CACTACGAGAAGCTGAAGGGCAGCCCCGAGGACAACGAGCAGAAGCAGCTGTTCGTGGAG
Hypa-Cas9  3721 CACTACGAGAAGCTGAAGGGCAGCCCCGAGGACAACGAGCAGAAGCAGCTGTTCGTGGAG
xCas9-3.7  3721 CACTACGAGAAGCTGAAGGGCAGCCCCGAGGACAACGAGCAGAAGCAGCTGTTCGTGGAG

Clone-1    3781 CAGCACAAGCACTACCTGGACGAGATCATCGAGCAGATCAGCGAGTTCAGCAAGCGCGTG
Clone-2    3781 CAGCACAAGCACTACCTGGACGAGATCATCGAGCAGATCAGCGAGTTCAGCAAGCGCGTG
Clone-3    3781 CAGCACAAGCACTACCTGGACGAGATCATCGAGCAGATCAGCGAGTTCAGCAAGCGCGTG
WT-Cas9    3781 CAGCACAAGCACTACCTGGACGAGATCATCGAGCAGATCAGCGAGTTCAGCAAGCGCGTG
eSpCas9    3781 CAGCACAAGCACTACCTGGACGAGATCATCGAGCAGATCAGCGAGTTCAGCAAGCGCGTG
Cas9-HF1   3781 CAGCACAAGCACTACCTGGACGAGATCATCGAGCAGATCAGCGAGTTCAGCAAGCGCGTG
evo-Cas9   3781 CAGCACAAGCACTACCTGGACGAGATCATCGAGCAGATCAGCGAGTTCAGCAAGCGCGTG
Hypa-Cas9  3781 CAGCACAAGCACTACCTGGACGAGATCATCGAGCAGATCAGCGAGTTCAGCAAGCGCGTG
xCas9-3.7  3781 CAGCACAAGCACTACCTGGACGAGATCATCGAGCAGATCAGCGAGTTCAGCAAGCGCGTG

Clone-1    3841 ATCCTGGCCGACGCCAACCTGGACAAGGTGCTGAGCGCCTACAACAAGCACCGCGACAAG
Clone-2    3841 ATCCTGGCCGACGCCAACCTGGACAAGGTGCTGAGCGCCTACAACAAGCACCGCGACAAG
Clone-3    3841 ATCCTGGCCGACGCCAACCTGGACAAGGTGCTGAGCGCCTACAACAAGCACCGCGACAAG
WT-Cas9    3841 ATCCTGGCCGACGCCAACCTGGACAAGGTGCTGAGCGCCTACAACAAGCACCGCGACAAG
eSpCas9    3841 ATCCTGGCCGACGCCAACCTGGACAAGGTGCTGAGCGCCTACAACAAGCACCGCGACAAG
Cas9-HF1   3841 ATCCTGGCCGACGCCAACCTGGACAAGGTGCTGAGCGCCTACAACAAGCACCGCGACAAG
evo-Cas9   3841 ATCCTGGCCGACGCCAACCTGGACAAGGTGCTGAGCGCCTACAACAAGCACCGCGACAAG
Hypa-Cas9  3841 ATCCTGGCCGACGCCAACCTGGACAAGGTGCTGAGCGCCTACAACAAGCACCGCGACAAG
xCas9-3.7  3841 ATCCTGGCCGACGCCAACCTGGACAAGGTGCTGAGCGCCTACAACAAGCACCGCGACAAG

Clone-1    3901 CCCATCCGCGAGCAGGCCGAGAACATCATCCACCTGTTCACCCTGACCAACCTGGGCGCC
Clone-2    3901 CCCATCCGCGAGCAGGCCGAGAACATCATCCACCTGTTCACCCTGACCAACCTGGGCGCC
Clone-3    3901 CCCATCCGCGAGCAGGCCGAGAACATCATCCACCTGTTCACCCTGACCAACCTGGGCGCC
WT-Cas9    3901 CCCATCCGCGAGCAGGCCGAGAACATCATCCACCTGTTCACCCTGACCAACCTGGGCGCC
eSpCas9    3901 CCCATCCGCGAGCAGGCCGAGAACATCATCCACCTGTTCACCCTGACCAACCTGGGCGCC
Cas9-HF1   3901 CCCATCCGCGAGCAGGCCGAGAACATCATCCACCTGTTCACCCTGACCAACCTGGGCGCC
evo-Cas9   3901 CCCATCCGCGAGCAGGCCGAGAACATCATCCACCTGTTCACCCTGACCAACCTGGGCGCC
Hypa-Cas9  3901 CCCATCCGCGAGCAGGCCGAGAACATCATCCACCTGTTCACCCTGACCAACCTGGGCGCC
xCas9-3.7  3901 CCCATCCGCGAGCAGGCCGAGAACATCATCCACCTGTTCACCCTGACCAACCTGGGCGCC
```

```
Clone-1    3961 CCCGCCGCCTTCAAGTACTTCGACACCACCATCGACCGCAAGCGCTACACCAGCACCAAG
Clone-2    3961 CCCGCCGCCTTCAAGTACTTCGACACCACCATCGACCGCAAGCGCTACACCAGCACCAAG
Clone-3    3961 CCCGCCGCCTTCAAGTACTTCGACACCACCATCGACCGCAAGCGCTACACCAGCACCAAG
WT-Cas9    3961 CCCGCCGCCTTCAAGTACTTCGACACCACCATCGACCGCAAGCGCTACACCAGCACCAAG
eSpCas9    3961 CCCGCCGCCTTCAAGTACTTCGACACCACCATCGACCGCAAGCGCTACACCAGCACCAAG
Cas9-HF1   3961 CCCGCCGCCTTCAAGTACTTCGACACCACCATCGACCGCAAGCGCTACACCAGCACCAAG
evo-Cas9   3961 CCCGCCGCCTTCAAGTACTTCGACACCACCATCGACCGCAAGCGCTACACCAGCACCAAG
Hypa-Cas9  3961 CCCGCCGCCTTCAAGTACTTCGACACCACCATCGACCGCAAGCGCTACACCAGCACCAAG
xCas9-3.7  3961 CCCGCCGCCTTCAAGTACTTCGACACCACCATCGACCGCAAGCGCTACACCAGCACCAAG

Clone-1    4021 GAGGTGCTGGACGCCACCCTGATCCACCAGAGCATCACCGGTCTGTACGAGACCCGCATC
Clone-2    4021 GAGGTGCTGGACGCCACCCTGATCCACCAGAGCATCACCGGTCTGTACGAGACCCGCATC
Clone-3    4021 GAGGTGCTGGACGCCACCCTGATCCACCAGAGCATCACCGGTCTGTACGAGACCCGCATC
WT-Cas9    4021 GAGGTGCTGGACGCCACCCTGATCCACCAGAGCATCACCGGTCTGTACGAGACCCGCATC
eSpCas9    4021 GAGGTGCTGGACGCCACCCTGATCCACCAGAGCATCACCGGTCTGTACGAGACCCGCATC
Cas9-HF1   4021 GAGGTGCTGGACGCCACCCTGATCCACCAGAGCATCACCGGTCTGTACGAGACCCGCATC
evo-Cas9   4021 GAGGTGCTGGACGCCACCCTGATCCACCAGAGCATCACCGGTCTGTACGAGACCCGCATC
Hypa-Cas9  4021 GAGGTGCTGGACGCCACCCTGATCCACCAGAGCATCACCGGTCTGTACGAGACCCGCATC
xCas9-3.7  4021 GAGGTGCTGGACGCCACCCTGATCCACCAGAGCATCACCGGTCTGTACGAGACCCGCATC

Clone-1    4081 GACCTGAGCCAGCTGGGCGGCGACGGCGGCTCCGGACCTCCAAAGAAAAAGAGAAAAGTA
Clone-2    4081 GACCTGAGCCAGCTGGGCGGCGACGGCGGCTCCGGACCTCCAAAGAAAAAGAGAAAAGTA
Clone-3    4081 GACCTGAGCCAGCTGGGCGGCGACGGCGGCTCCGGACCTCCAAAGAAAAAGAGAAAAGTA
WT-Cas9    4081 GACCTGAGCCAGCTGGGCGGCGACGGCGGCTCCGGACCTCCAAAGAAAAAGAGAAAAGTA
eSpCas9    4081 GACCTGAGCCAGCTGGGCGGCGACGGCGGCTCCGGACCTCCAAAGAAAAAGAGAAAAGTA
Cas9-HF1   4081 GACCTGAGCCAGCTGGGCGGCGACGGCGGCTCCGGACCTCCAAAGAAAAAGAGAAAAGTA
evo-Cas9   4081 GACCTGAGCCAGCTGGGCGGCGACGGCGGCTCCGGACCTCCAAAGAAAAAGAGAAAAGTA
Hypa-Cas9  4081 GACCTGAGCCAGCTGGGCGGCGACGGCGGCTCCGGACCTCCAAAGAAAAAGAGAAAAGTA
xCas9-3.7  4081 GACCTGAGCCAGCTGGGCGGCGACGGCGGCTCCGGACCTCCAAAGAAAAAGAGAAAAGTA

Clone-1    4141 TACCCCTACGACGTGCCCGACTACGCC
Clone-2    4141 TACCCCTACGACGTGCCCGACTACGCC
Clone-3    4141 TACCCCTACGACGTGCCCGACTACGCC
WT-Cas9    4141 TACCCCTACGACGTGCCCGACTACGCC
eSpCas9    4141 TACCCCTACGACGTGCCCGACTACGCC
Cas9-HF1   4141 TACCCCTACGACGTGCCCGACTACGCC
evo-Cas9   4141 TACCCCTACGACGTGCCCGACTACGCC
Hypa-Cas9  4141 TACCCCTACGACGTGCCCGACTACGCC
xCas9-3.7  4141 TACCCCTACGACGTGCCCGACTACGCC
```
